# Health-related quality of life in Myalgic Encephalomyelitis/Chronic Fatigue Syndrome and Post COVID-19 Condition: a systematic review

**DOI:** 10.1186/s12967-025-06131-z

**Published:** 2025-03-13

**Authors:** Breanna Weigel, Maira Inderyas, Natalie Eaton-Fitch, Kiran Thapaliya, Sonya Marshall-Gradisnik

**Affiliations:** 1 National Centre for Neuroimmunology and Emerging Diseases, Griffith University, 1 Parklands Drive, Southport, Gold Coast, QLD 4222 Australia; 2 Consortium Health International for Myalgic Encephalomyelitis, Griffith University, Gold Coast, QLD 4222 Australia; 3 School of Pharmacy and Medical Sciences, Griffith University, Gold Coast, QLD 4222 Australia

**Keywords:** Myalgic Encephalomyelitis/Chronic Fatigue Syndrome, Post COVID-19 Condition, Post-Acute Sequelae of COVID-19, Long COVID, Health-related quality of life

## Abstract

**Purpose:**

Myalgic Encephalomyelitis/Chronic Fatigue Syndrome (ME/CFS) and Post COVID-19 Condition (PCC) are debilitating, chronic multi-systemic illnesses that require multidisciplinary care. However, people with ME/CFS (pwME/CFS) and people with PCC (pwPCC) are often precluded from accessing necessary disability and social support services. These unmet care needs exacerbate the existing illness burdens experienced by pwME/CFS and pwPCC. To deliver appropriate care and optimise health outcomes for pwME/CFS and pwPCC, the development of evidence-based healthcare policies that recognise the disabling impacts of these illnesses must be prioritised. This systematic review summarises the health-related quality of life (HRQoL) of pwME/CFS and pwPCC when compared with healthy controls (HCs) to elucidate the impacts of these illnesses and guide healthcare policy reform.

**Methods:**

CINAHL, Embase, MEDLINE, PubMed, PsycINFO and the Web of Science Core Collection were systematically searched from 1st January 2003 to 23rd July 2024. Eligible publications included observational studies capturing quantitative HRQoL data among pwME/CFS or pwPCC when compared with HCs. The use of validated patient-reported outcome measures (PROMs) was mandatory. Eligible studies were also required to employ the most stringent diagnostic criteria currently available, including the Canadian Consensus Criteria or International Consensus Criteria for ME/CFS and the World Health Organization case definition for PCC (PROSPERO ID: CRD42024501309).

**Results:**

This review captured 16 studies, including eight studies among pwME/CFS, seven studies among pwPCC and one study among both illness cohorts. Most participants were female and middle-aged. All pwPCC had experienced prolonged COVID-19 symptoms for at least three months. When compared with HCs, all HRQoL domains were significantly impaired among pwME/CFS and pwPCC. Both illnesses had a salient impact on physical health, including pain and ability to perform daily and work activities. While direct comparisons between pwME/CFS and pwPCC were limited by inconsistencies in the PROMs employed, comparable impact trends across HRQoL domain scores were observed.

**Conclusion:**

ME/CFS and PCC have similar, profound impacts on HRQoL that warrant access to multidisciplinary disability and social support services. Future research must harmonise HRQoL data collection and prioritise longitudinal investigations among pwME/CFS and pwPCC to characterise PCC subgroups (including those fulfilling ME/CFS criteria) and predictors of prognosis.

**Supplementary Information:**

The online version contains supplementary material available at 10.1186/s12967-025-06131-z.

## Introduction

Myalgic Encephalomyelitis/Chronic Fatigue Syndrome (ME/CFS) is a debilitating chronic multi-systemic illness that affects approximately 0.89% of the global population [1]. The clinical presentation of ME/CFS is hallmarked by post-exertional malaise, which is defined as the exacerbation of symptoms following any physical, mental or emotional exertion [2–5]. Other typical ME/CFS symptoms include cognitive dysfunction, muscle and joint pain, disruptions to and unrefreshing sleep, flu-like symptoms and autonomic dysfunction (including impairments in thermoregulation, gastrointestinal upset, respiratory difficulty and cardiovascular issues) [2–5]. Although the aetiology of ME/CFS remains incompletely defined, infectious illness precedes approximately three-quarters of cases prior to onset [3, 6–11].

The extensive symptom burden of ME/CFS has disabling impacts on people who live with the condition [12–14]. Consequently, people with ME/CFS (pwME/CFS) are limited from engaging in their pre-morbid daily, social and working life and may be house- or bed-bound [2, 3, 8, 15]. While recent advancements characterising the pathophysiology of ME/CFS have identified nascent pharmacotherapeutic targets [16–18], there does not currently exist a cure for the condition [3–5]. Additionally, as recovery is reported in less than 10% of cases, the prognosis for most pwME/CFS is life-long illness [3, 4, 6].

ME/CFS symptomatology is mirrored in Post COVID-19 Condition (PCC) — also known as “Long COVID” or “Post-Acute Sequelae of COVID-19” [3, 8, 13, 19–21]. PCC is characterised by symptoms that are persistent or new in onset following Severe Acute Respiratory Syndrome Coronavirus 2 (SARS-CoV-2) infection [21–24]. The prevalence of PCC among COVID-19 survivors is approximately 10 to 30% [25, 26]. People with PCC (pwPCC) similarly face multi-systemic symptoms and a reduced ability to perform typical life activities [13, 21, 27]. The long-term prognosis of PCC is unclear and, like ME/CFS, the condition currently remains incurable [21, 22].

Shared disruptions to physiological functioning and overlapping genetic changes have also been documented among pwME/CFS and pwPCC [16, 21, 28–30]. It is therefore anticipated that there is a common aetiopathogenesis between ME/CFS and at least a subtype of PCC [3, 9, 19–21, 29]. This is further supported by the two conditions’ remarkable clinical similarities, shared post-infectious onset and absence of other pathologies to explain symptoms [3, 9, 19–21]. While investigations into the exact pathomechanisms underpinning these conditions are ongoing, considering pwME/CFS and pwPCC collectively is nevertheless beneficial from a public health perspective in the interim. Importantly, epidemiological studies have identified a comparable presentation of hallmark ME/CFS symptoms (such post-exertional malaise and cognitive dysfunction) among pwPCC and between 40% and 60% of pwPCC fulfil ME/CFS case criteria [13, 31, 32]. This signifies the potential benefit of shared management approaches for the two conditions [3, 21, 33]. Consequently, the development of care pathways may be accelerated for PCC, despite the condition’s novelty, via guidance from existing management approaches for ME/CFS [3, 9, 19–21]. Additionally, research advancements in PCC treatments may also have relevance for pwME/CFS [3, 9, 19–21].

Currently, treating ME/CFS and PCC largely involves the management of symptoms, as well as mitigating the associated challenges of living with these illnesses via assistance from disability and social support services [3, 4, 21, 22, 34, 35]. However, many pwME/CFS and pwPCC do not receive such supports due to poor service accessibility, inappropriate eligibility requirements and extensive costs [15, 36–38]. This lack of necessary care compounds existing illness burdens, leading to further deteriorations in health and restrictions on one’s ability to participate in life and the community [15, 34, 39]. These care inequities among pwME/CFS and pwPCC are driven by insufficient recognition of the disabling nature of these illnesses in healthcare policies that dictate access to support services [36–38].

The present systematic review therefore serves to elucidate the pervasive impacts of ME/CFS and PCC on people who live with these conditions to inform and guide healthcare policy reform, as well as future research investigating HRQoL among these illness cohorts. Existing literature examining health-related quality of life (HRQoL) among pwME/CFS and pwPCC when compared with healthy controls (HCs) are consolidated and analysed herein. HRQoL impact patterns among pwME/CFS and pwPCC are also compared. Specifically, this review is guided by the following research questions: (a) In which domains is HRQoL significantly compromised among pwME/CFS and pwPCC when compared with HCs? and (b) Do pwME/CFS and pwPCC experience the same impairments in HRQoL?

In the context of the present review, pwME/CFS and pwPCC are defined as those fulfilling the most stringent corresponding diagnostic criteria available. Hence, all participants with ME/CFS meet the Canadian Consensus Criteria (CCC) [40] or International Consensus Criteria (ICC) [2] and all participants with PCC have an illness duration of at least 12 weeks consistent with the World Health Organization (WHO) case definition [23]. Although other PCC case definitions are similarly characterised by a minimum illness duration threshold of 12 weeks [22, 24], the WHO case definition additionally requires symptoms to have a significant impact on functioning [23]. Current PCC case definitions are broad with low specificity and, hence, likely capture a collection of post-COVID-19 sequelae beyond chronic multi-systemic illness [9, 21, 41]. In the continuing absence of an objective, confirmatory test to identify PCC cases, the most stringent existing case criteria (being the WHO case definition) was preferred to minimise the potential for the results observed to be explained by other medical conditions.

## Methods

This review was synthesised in conformity with the 2020 Preferred Reporting Items for Systematic Reviews and Meta-Analyses (PRISMA) statement [42] (S1 Table, Additional file 1). The review protocol has been registered with and is accessible via the International Prospective Register of Systematic Reviews (PROSPERO ID: CRD42024501309).

### Information sources

Database searches were performed by two independent reviewers (BW and MI) on 23rd July 2024. Cumulative Index to Nursing and Allied Health Literature (CINAHL, EBSCOHost), Embase (Elsevier), MEDLINE (Ovid), PubMed, PsycINFO (Ovid) and Web of Science Core Collection were searched for records published from 2003 (the publication year of the ME/CFS case criteria of interest) to the time at which the search was performed. Manual backward and forward citation searching was performed by scanning the reference and cited-by lists of eligible publications using Google Scholar and Web of Science.

### Search strategy

The terms “Chronic Fatigue Syndrome”, “Long COVID” and “Health-Related Quality of Life” were searched in the following controlled vocabulary databases: CINAHL subject headings, Emtree, Medical Subject Headings (MeSH) and PsycINFO thesaurus. All keywords and synonyms captured by the controlled vocabulary terms were then compiled to determine the illness- and HRQoL-specific search terms. Manual citation searching revealed relevant publications that were excluded from preliminary searches, as terms relating to “function” and “wellbeing” were employed to refer to HRQoL. Consequently, the HRQoL-specified search terms were expanded in the final searches to include these terms. Each database (except Web of Science Core Collection) was searched using controlled vocabulary and free-text terms combined with the Boolean operator “OR”. Equivalent subjects were inactivated for CINAHL and MEDLINE and mapping was inactivated for Embase and PsycINFO. The illness- and HRQoL-specific queries were combined with the Boolean operator “AND” to return the final search results. The complete search strategy for each database is provided in S2 Table, Additional file 1.

### Selection process and eligibility criteria

The records returned from the database searches were exported to EndNote 20 (Clarivate, Philadelphia, Pennsylvania) [43] for storage and screening. EndNote’s “Find Duplicates” feature was used to identify matches based on author name, publication year, publication title and DOI. All resulting records were then manually screened for residual duplicates as an additional quality assurance measure. The remaining records were screened according to the following title and abstract screening criteria: (1) original, observational research (excluding case reports, modelling or in vitro studies and cost-effectiveness analyses) that is not associated with a therapeutic or rehabilitative intervention; (2) written in English or with an English translation; (3) at least one comparator group that is comprised of pwME/CFS or pwPCC who are identified using at least one of the illness-specific keywords in S2 Table, Additional file 1 or with reference to one of the published case criteria of interest; (4) adult participants not belonging to a group of special interest based on health status (such as people with an existing comorbidity or those who have experienced a specific medical procedure) or demographics (such as nurses, shift workers or carers); (5) collection of quantitative HRQoL data using a generalised HRQoL patient-reported outcome measure (PROM) that is not specific to a particular condition, component of HRQoL or body system, as identified through the use of at least one of the HRQoL-specific keywords in S2 Table, Additional file 1 or mention of a validated HRQoL PROM; and (6) collection of control HRQoL data.

Publications fulfilling all the title and abstract screening criteria were subjected to full-text screening. To be considered eligible for inclusion, records were required to: (1) be a complete research article (excluding conference abstracts and preprints) with the full English text retrievable via institutional access; (2) have acquired ethical approval and informed consent from all participants; (3) capture study participants fulfilling the corresponding case criteria of interest: (a) pwME/CFS meeting the CCC or ICC or (b) pwPCC experiencing post-COVID-19 symptoms for at least 12 weeks consistent with the WHO case definition; (4) include a control dataset of HRQoL values that is clearly identified as either (a) population norms, (b) HCs without a history of SARS-CoV-2 infection or (c) participants’ pre-morbid health status data; (5) not include study participants under the age of 18 years; (6) report HRQoL as primary outcome data collected via at least one validated PROM; and (7) report at least the *p*-value resulting from comparisons of HRQoL scores.

Records that did not meet these eligibility criteria were excluded from the review. Limitations were not imposed on the included publications by study setting or, for longitudinal studies, the time between the collection of baseline and follow-up data.

### Data collection process, data items and effect measures

The complete data of interest included: (1) first author; (2) year of publication; (3) study design; (4) study location; (5) recruitment methods and, for studies among pwPCC: (a) the SARS-CoV-2 variant or variants of interest and (b) the percentage of the PCC cohort that had been hospitalised for acute COVID-19 illness; (6) comparator groups and the number of participants; (7) criteria employed to define each comparator group; (8) mean age (in years) of each comparator group; (9) mean illness duration (in years) of each comparator group (except HCs); (10) distribution of females or women per comparator group; (11) PROM or PROMs employed to collect HRQoL data; and (12) HRQoL data and *p*-values (measure of effect). This data was extracted from the eligible publications, tabulated and analysed in narrative synthesis.

### Quality assessment

The quality of the design and methods of the included studies’ was assessed using the internationally recognised and validated Joanna Briggs Institute (JBI) quality assessment tool for analytical cross-sectional studies (2020 version) [44]. For each of the JBI checklist items, the potential responses were yes, no and unclear. In the context of the present review, Items Three and Four correspond to the employment of the CCC or ICC to identify pwME/CFS and the use of criteria consistent with the WHO case definition to identify pwPCC. Item Seven refers to the employment of validated PROMs to capture HRQoL data. All eligible publications therefore fulfilled Items Three, Four and Seven. Appraisal of the reporting of statistical methods and results (Item Eight) was informed by the Statistical Analyses and Methods in the Published Literature guidelines [45].

## Results

**Fig. 1:**
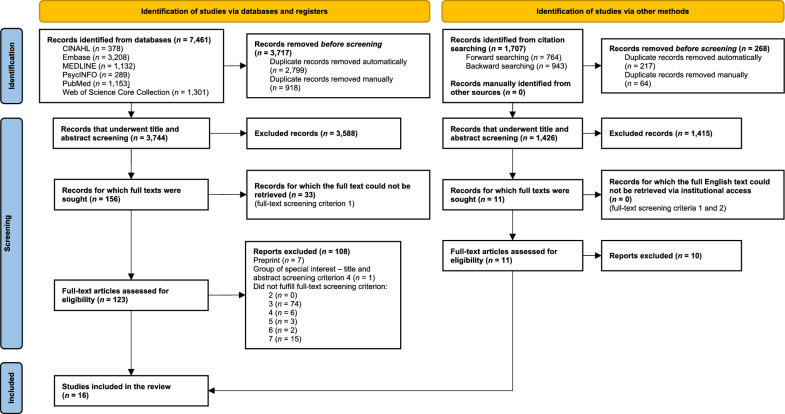
PRISMA 2020 flow diagram depicting the record screening process. Figure generated in Microsoft Word and retrieved from Page et al*.* [42]. Abbreviations: *CINAHL* Cumulative Index for Nursing and Allied Health Literature

Database searches yielded a combined total of 7461 records (Fig. 1). Upon exporting the records to EndNote 20, 2799 duplicates were automatically removed using EndNote’s “Find Duplicates” feature. Manual screening for duplicates identified a further 918 matches, resulting in a total of 3717 duplicates removed. Hence, 3744 records were eligible for title and abstract screening. The abstracts of two records could not be retrieved via institutional access and five records had been redacted. Of the remaining 3737 records, 3581 did not fulfil the title and abstract screening criteria. Thus, 156 records proceeded to full-text screening.

Of the 156 records screened, an English translation of the full text was not available for two records. Seven records were preprints and 31 records were conference abstracts. Another record was excluded as only pwPCC aged over 65 years were included. Of the remaining 115 records for which the full text was available and written in English, 100 were ineligible. The criteria employed to identify participants with ME/CFS or PCC were unclear or were not consistent with the case criteria of interest for 74 records (eligibility criterion three). Six records did not include at least one comparator group of pwME/CFS or pwPCC and a control dataset consisting of HCs, population norms or pre-morbid health status data (eligibility criterion four). The study population included people aged under 18 years for three records (eligibility criterion five). HRQoL data was not collected via a validated PROM for two records (eligibility criterion six). Neither the level of significance nor the *p*-value of statistical tests comparing HRQoL data between the study cohorts were reported for 15 records (eligibility criterion seven).

Forward and backward citation searching of the 15 eligible articles identified an additional publication suitable for inclusion [46]. Therefore, a total of 16 publications are captured in the present review [12, 13, 46–59].

### Study and participant characteristics

This review included eight studies among pwME/CFS [12, 46–52], seven studies among pwPCC [53–59] and one study among both pwME/CFS and pwPCC [13]. Ethical approval and informed consent had been acquired in all the included publications. The study information (data items 1 to 5, 7, and 11) and participant characteristics (data items 6 and 8 to 10) are summarised in Table 1. All data extracted from the eligible publications are provided in Table 2.

**Table 1:** Summary statistics of the study and participant characteristics of the eligible publications

	Studies among pwME/CFS (*n* = 8)	Studies among pwPCC (*n* = 7)	Studies among pwME/CFS and pwPCC (*n* = 1)
Publication year (*n* (%), studies)			
2003–2009	0 (0.0)	0 (0.0)	0 (0.0)
2010–2019	6 (75.0)	0 (0.0)	0 (0.0)
2020–2024	2 (25.0)	7 (100.0)	1 (100.0)
Study design (*n* (%), studies)			
Cross-sectional	7 (87.5)	6 (85.7)	1 (100.0)
Prospective panel	1 (12.5)	2 (28.6)	0 (0.0)
Study location (*n* (%), studies)			
Americas	3 (37.5)	1 (14.3)	0 (0.0)
Africa	0 (0.0)	0 (0.0)	0 (0.0)
Asia	1 (12.5)	1 (14.3)	0 (0.0)
Europe	4 (50.0)	4 (57.1)	0 (0.0)
Mediterranean and Middle East	0 (0.0)	0 (0.0)	0 (0.0)
Oceania	1 (12.5)	1 (14.3)	1 (100.0)
International or cross-cultural	0 (0.0)	0 (0.0)	0 (0.0)
Recruitment methods (*n* (%), studies)			
Convenience sample	8 (100.0)	5 (71.4)	1 (100.0)
Total population sample	0 (0.0)	2 (28.6)	0 (0.0)
Hospitalisation status for acute COVID-19 illness			
Non-hospitalised participants (*n* (%), studies)	NA	2 (28.6)	0 (0.0)
Hospitalised participants (*n* (%), studies)	NA	1 (14.3)	0 (0.0)
Non-hospitalised and hospitalised participants (*n* (%), studies)	NA	3 (42.9)	0 (0.0)
Hospitalised (*x̄* (min–max), % participants)^a^	NA	17.8 (3.0–43.8)	NA
Missing (*n* (%), studies)	NA	1 (14.3)	1 (100.0)
SARS-CoV-2 variant/s of interest (*n* (%), studies)			
Alpha	NA	0 (0.0)	0 (0.0)
Delta	NA	0 (0.0)	0 (0.0)
Omicron	NA	1 (14.3)	0 (0.0)
Combination	NA	0 (0.0)	0 (0.0)
Missing	NA	6 (85.7)	1 (100.0)
Comparator groups (*n* (%), studies)			
HCs			
Non-infected	NA	5 (71.4)	0 (0.0)
“Healthy”	8 (100.0)	2 (28.6)	1 (100.0)
PwME/CFS or pwPCC per study (*M* (min–max), *n* participants)	30 (10–87)	80 (33–450)	61 (NA) pwME/CFS 31 (NA) pwPCC
Missing (*n* (%), studies)	0 (0.0)	0 (0.0)	0 (0.0)
ME/CFS case definition/s employed (*n* (%), studies)			
CCC	6 (75.0)	NA	1 (100.0)
ICC	2 (25.0)	NA	1 (100.0)
PCC case definition/s employed (*n* (%), studies)			
WHO	NA	5 (71.4)	1 (100.0)
Persistent symptoms for at least 12 weeks	NA	1 (14.3)	0 (0.0)
Persistent symptoms at follow-up	NA	1 (14.3)	0 (0.0)
Age^a^			
Means (*x̄* (min–max), years)	47.56 (36.02–53)^b^	46.28 (35–53.24)^c^	0 (0.0)
Medians (*M* (min–max), years)	30.0 (NA)^d^	37 (NA)^e^	42.00 (NA) pwME/CFS 47.00 (NA) pwPCC
Missing (*n* (%), studies)	0 (0.0)	0 (0.0)	0 (0.0)
Illness duration^a^			
Means (*x̄* (min–max), years)	14.56 (7.47–21)^f^	1.15 (0.73–2.00)^g^	0 (0.0)
Medians (*M* (min–max), years)	7.0 (NA)^h^	0.78 (0.67–0.89)^i^	10.00 (NA) pwME/CFS 0.33 (NA) pwPCC
Missing (*n* (%), studies)	2 (25.0)	3 (42.9)	0 (0.0)
Females/women (*x̄* (min–max), % participants)^a^	81.2 (51.1–100.0)	63.2 (43.3–81.8)	78.7 (NA) pwME/CFS 64.5 (NA) pwPCC
Missing (*n* (%), studies)	0 (0.0)	0 (0.0)	0 (0.0)

HRQoL PROM/s employed (*n* (%), studies)			
EQ-5D-3L and EQ-VAS	0 (0.0)	1 (14.3)	0 (0.0)
EQ-5D-5L and EQ-VAS	0 (0.0)	3 (42.9)	0 (0.0)
Dr Bell’s CFIDS Disability Scale	0 (0.0)	0 (0.0)	1 (100.0)
Karnofsky Performance Status Index	2 (25.0)	0 (0.0)	0 (0.0)
RAND-36	1 (12.5)	0 (0.0)	0 (0.0)
SF-12	0 (0.0)	2 (28.6)	0 (0.0)
SF-36	6 (75.0)	1 (14.3)	1 (100.0)
Sheehan Disability Scale	0 (0.0)	1 (14.3)	0 (0.0)
WHODAS 2.0	1 (12.5)	1 (14.3)	1 (100.0)
WHOQOL-BREF	0 (0.0)	1 (14.3)	0 (0.0)

*CCC* Canadian Consensus Criteria, *CFIDS* Chronic Fatigue and Immune Dysfunction, *EQ-5D-3L* EuroQol 5-Dimension 3-Level questionnaire, *EQ-5D-5L* EuroQol 5-Dimension 5-Level questionnaire, *EQ-VAS* EuroQol Visual Analogue Scale, *HC* Healthy control, *ICC* International Consensus Criteria, *M* Median, *ME/CFS* Myalgic Encephalomyelitis/Chronic Fatigue Syndrome, *NA* Not applicable, *NR* Not reported, *PCC* Post COVID-19 Condition, *PwME/CFS* People with Myalgic Encephalomyelitis/Chronic Fatigue Syndrome, *PwPCC* People with Post COVID-19 Condition, *SARS-CoV-2* Severe Acute Respiratory Syndrome Coronavirus-2, *SF-12* 12-Item Short-Form Health Survey, *SF-36* 36-Item Short-Form Health Survey, *WHO* World Health Organization, *WHODAS 2.0* World Health Organization Disability Assessment Schedule version 2.0

^a^Values are reported to the number of decimal points provided in the corresponding publication. Where applicable, statistics have been generated based on values converted to years for studies providing data in days or weeks

^b^Data available for seven studies [12, 46–50, 52]

^c^Data available for five studies [53–57]

^d^Data available for one study [51]

^e^Data available for one study [59]

^f^Data available for five studies [12, 46–48, 50]

^g^Data available for two studies [53, 55]

^h^Data available for one study [51]

^i^Data available for two studies [56, 59]

**Table 2:** Complete study and participant characteristics extracted from the eligible publications

Reference	Year	Study design	Study location	Recruitment methods (% hospitalised, if applicable)	Comparator groups (*n*)	Diagnostic criteria	Mean age in years (*x̄* (*s*))	Mean illness duration in years (*x̄* (*s*))	Females/women (%)	HRQoL PROM/s employed
Ariza et al. [53]	2024	Cross-sectional	Spain	Convenience sample (43.8% hospitalised)	M-pwPCC (*n* = 207)	WHO case definition and a history of mild acute COVID-19 illness	47.55 (9.54)^c^ M < H M < ICU	1.01 (0.56)^b,c^ M > H M > ICU	79.7^c^ M > H M > ICU	EQ-5D-3L and EQ-VAS Spanish version (2018) [62] WHODAS 2.0 (2010) [66] WHOQOL-BREF Spanish version (2020) [87]
					H-pwPCC (*n* = 80)	WHO case definition and a history of hospitalisation for acute COVID-19 illness	53.24 (8.68)^c^ H > HCs H > M	0.84 (0.42)^b,c^ H < M	50.0^c^ H < HCs H < ICU	
					ICU-pwPCC (*n* = 81)	WHO case definition and a history of ICU admission for acute COVID-19 illness	52.82 (8.47)^c^ ICU > HCs ICU > M	0.73 (0.32)^b,c^ ICU < M	46.9^c^ ICU < HCs ICU < M	
					Non-infected HCs (*n* = 124)	NA	46.78 (10.03)^c^ HCs < H HCs < ICU	NA	75.0^c^ HCs > H HCs > ICU	
Cai et al*.* [54]	2023	Cross-sectional and prospective panel	China	Total population sample (100.0% hospitalised)	PwPCC (*n* = 450)	WHO case definition following SARS-CoV-2 Omicron infection	42.32 (12.67)^c^ PCC < HCs	NR	43.3^c^ PCC > HCs	EQ-5D-5L and EQ-VAS (version unclear)
					Non-infected HCs (*n* = 979)	NA	43.51 (10.70)^c^ HCs > PCC	NA	36.3^c^ HCs < PCC	
Calvache-Mateo et al*.* [55]	2023	Cross-sectional	Spain	Convenience sample (0.0% hospitalised)	PwPCC (*n* = 69)	WHO case definition	44.99 (2.79)	2.00 (0.22)^d^	75.26	EQ-5D-5L and EQ-VAS (version unclear)
					Non-infected HCs (*n* = 67)	NA	44.67 (3.11)	NA	75.51	
Cambras et al*.* [47]	2018	Prospective panel	Spain	Convenience sample	PwME/CFS (*n* = 10/10^e^)	CCC	50.6 (1.39)	8.4 (2.6)	100.0	SF-36 Spanish version (1995) [88]
					HCs (*n* = 10/8^e^)	NA	48.7 (4.13)	NA	100.0	
Chang et al*.* [48]	2021	Cross-sectional	United States	Convenience sample	PwME/CFS (*n* = 20)	ICC^f^	47.4 (11.6)	14.5 (11.8)	65.0	SF-36 (version unclear) and Karnofsky Performance Status Index [60]
					HCs (*n* = 10)	NA	46.8 (9.2)	NA	60.0	

De Sousa et al*.* [56]	2022	Cross-sectional	Brazil	Convenience sample (0.0% hospitalised)	PwPCC (*n* = 40)	Persistent symptoms for a minimum of 12 weeks	35 (7.2)	0.67 (0.42–1)^a,b^	60.0	SF-36 Brazilian Portuguese version (1999) [89]
					Non-infected HCs (*n* = 40)	NA	34 (9.7)	NA	57.5	
De Vega et al. [49]	2017	Cross-sectional	United States	Convenience sample	PwME/CFS (*n* = 49)	CCC	49.4 (13.3)^g^	NR	100.0	RAND-36 version 1.0 (1993) [90]
					HCs (*n* = 25)	NA	51.1 (13.5)^g^	NA	100.0	
Espinar-Herranz et al. [57]	2023	Cross-sectional	Spain	Convenience sample (hospitalisation data not provided)	PwPCC (*n* = 57)	WHO case definition	48.06 (8.22)	NR	70.2^c^ PCC > HCs	SF-12 Spanish version (1998) [91]
					HCs (*n* = 29)	NA	44.22 (13.19)	NA	27.6^c^ HCs < PCC	
Johnston et al*.* [12]	2014	Cross-sectional	Australia	Convenience sample	PwME/CFS (*n* = 22)	ICC	49.3 (13.2)	19.0 (10.2)	95	Ware-36/SF-36 version 1 (1992) [92] WHODAS 2.0 (2010) [66]
					HCs (*n* = 30)	NA	49.7 (10.9)	NA	66	
Maroti et al*.* [50]	2018	Cross-sectional	Sweden	Convenience sample	PwME/CFS (*n* = 37–38^h^)	CCC	42.74 (11.54)	7.47 (5.70)^b^	78.9	SF-36 Swedish version (1998) [93]
					HCs (*n* = 30)	NA	44.47 (11.56)	NA	70.0	
Naviaux et al. [46]	2016	Cross-sectional	United States	Convenience sample	Females with ME/CFS (*n* = 23)	CCC	52 (11.99)^g^	17 (11.03)^g^	100.0	Karnofsky Performance Status Index [60]
					Males with ME/CFS (*n* = 22)	CCC	53 (13.13)^g^	21 (14.07)^g^	0.0	
					Female HCs (*n* = 21)	NA	48 (12.83)^g^	NA	100.0	
					Male HCs (*n* = 18)	NA	53 (14.85)^g^	NA	0.0	
Nehme et al*.* [58]	2022	Prospective panel	Switzerland	Total population sample (6.5% hospitalised)	PwPCC (*n* = 148)	Persistent symptoms at seven- and 15- months follow-up	NR	NR	61.4	Ware-12/SF-12 version 1 (1996) [94] Sheehan Disability Scale [65]
					Non-infected HCs (*n* = 616)	NA	NR	NA	69.5	

Ryabkova et al. [51]	2023	Cross-sectional	Russia	Convenience sample	PwME/CFS (*n* = 11)	CCC	30.0 (27.0–45.0)^a^	7.0 (6.0–10.5)^a^	81.8	Ware-36/SF-36 version 1 (1992) [92]
					HCs (*n* = 11)	NA	33.0 (27.0–49.0)^a^	NA	72.7	
Seeley et al*.* [59]	2023	Cross-sectional	Australia	Convenience sample (3.0% hospitalised)	PwPCC (*n* = 33)	WHO case definition	37 (15)^a^	0.89 (0.77)^a,j^	81.8	EQ-5D-5L and EQ-VAS (version unclear)
					HCs (*n* = 33)^i^	NA	28 (23)^a^	NA	81.8	
Strand et al*.* [52]	2019	Cross-sectional	Norway	Convenience sample	PwME/CFS (*n* = 87)	CCC	36.02 (11.60)^c^	NR	80.5	Ware-36/SF-36 version 1 (1992) [92]
					HCs (*n* = 94)	NA	28.64 (7.30)	NA	70	
Weigel et al. [13]	2024	Cross-sectional	Australia	Convenience sample (hospitalisation data not provided)	PwME/CFS (*n* = 61)	CCC and ICC	42.00 (30.15–52.20)^c^ ME/CFS < PCC	10.00 (5.00–18.00)^c^ ME/CFS > PCC	78.7	SF-36 version 2 (2000) [95] WHODAS 2.0 (2010) [66]
					PwPCC (*n* = 31)	WHO case definition	47.00 (41.00–54.00)^c^ PCC > ME/CFS PCC > HCs	0.33 (0.25–0.60)^c^ PCC < ME/CFS	64.5	
					HCs (*n* = 54)^i^	NA	42.50 (25.85–52.00)^c^ HCs < PCC	NA	68.5	

*CCC* Canadian Consensus Criteria, *EQ-5D-3L* EuroQol 5-Dimension 3-Level questionnaire, *EQ-5D-5L* EuroQol 5-Dimension 5-Level questionnaire, *EQ-VAS* EuroQol Visual Analogue Scale, *HC* Healthy control, *HRQoL* Health-related quality of life, *ICC* International Consensus Criteria, *ICU* Intensive care unit, *ME/CFS* Myalgic Encephalomyelitis/Chronic Fatigue Syndrome, *NA* Not applicable, *NR* Not reported, *PROM* Patient-reported outcome measure, *PwME/CFS* People with Myalgic Encephalomyelitis/Chronic Fatigue Syndrome, *PwPCC* People with Post COVID-19 Condition, *SARS-CoV-2* Severe Acute Respiratory Syndrome Coronavirus-2, *SF-12* 12-Item Short-Form Health Survey, *SF-36* 36-Item Short-Form Health Survey, *WHO* World Health Organization, *WHODAS 2.0* World Health Organization Disability Assessment Schedule version 2.0

^a^*M* and quartile 1 to quartile 3 (*Q1–Q3*) or interquartile range

^b^Values extracted from the text have been converted from months to years

^c^Significantly different when compared with comparator group or groups

^d^Values extracted from the text have been converted from weeks to years

^e^Values at baseline/follow-up time points

^f^Participants from this study cohorts were people with “severe” and “very severe” ME/CFS. To be considered eligible for this study, participants were required (in addition to fulfilling the ICC) to return an SF-36 Physical Functioning score of less than 70, return a Karnofksy Performance Status Index score of less than 70, and spend at least 14 h per day in a reclined position

^g^Values extracted from the text have been converted from standard error to standard deviation

^h^The number of pwME/CFS in this publication was provided as a range

^i^HCs were defined as “healthy”; however, their history of acute COVID-19 illness was not specified

^j^Values extracted from the text have been converted from days to years

Most studies among pwME/CFS (*n* = 6, 75.0% [12, 46, 47, 49, 50, 52]) were published between 2014 and 2020. Only three of the included studies [13, 48, 51] provided HRQoL data among pwME/CFS in the pandemic era. All studies among pwPCC (*n* = 7, 100.0% [53–59]) were published from 2022 onwards. The included studies were largely cross-sectional in nature [12, 13, 46, 48–59]. Three studies had a longitudinal study design (*n* = 1, 12.5% study among pwME/CFS [47]; *n* = 2, 28.6% studies among pwPCC [54, 58]). However, only Cambras et al. [47] provided comparisons of HRQoL data with HCs over time.

Europe was the most common study location [47, 50–53, 55, 57, 58]. The remaining studies were based in Australia (*n* = 1, 12.5% study among pwME/CFS [12]; *n* = 1, 14.3% study among pwPCC [59]; *n* = 1, 100.0% study among pwME/CFS and pwPCC [13]), the United States (*n* = 3, 37.5% studies among pwME/CFS [46, 48, 49]), Brazil (*n* = 1, 14.3% study among pwPCC [56]) and China (*n* = 1, 14.3% study among pwPCC [54]).

Participants were recruited via convenience sampling in all studies among pwME/CFS (*n* = 8, 100.0% [12, 46–52]). Five studies among pwPCC (71.4% [53, 55–57, 59]) similarly consisted of convenience samples. The remaining two studies among pwPCC (28.6% [54, 58]) employed total population sampling of all laboratory-confirmed COVID-19 cases recorded by testing centres at local hospitals. Studies among pwPCC most commonly consisted of both hospitalised and non-hospitalised COVID-19 survivors (*n* = 3, 42.9% [53, 58, 59]). The proportion of participants in these studies who had previously been hospitalised ranged from 3.0% to 43.8% [53, 58, 59]. None of the participants had been hospitalised for acute COVID-19 illness in two studies (28.6% [55, 56]). At the time of data collection, Cai et al*.* [54] reported the dominant SARS-CoV-2 variant to be Omicron. None of the other studies capturing pwPCC as a comparator group [13, 53, 55–59] reported the infecting variants.

All studies compared pwME/CFS or pwPCC with HCs and none used population norms or pre-illness heath status data. HCs were identified as “healthy” participants in three studies capturing pwPCC as a comparator group [13, 57, 59]; however, the HCs’ history of SARS-CoV-2 infection was not specified. All other studies among pwPCC (*n* = 5/7, 71.4% [53–56, 58]) confirmed that HCs had no known history of acute COVID-19 illness. The number of ME/CFS and PCC participants per study ranged from 10 to 87 (median (*M*) = 37) and 33 to 450 (*M* = 75), respectively. Maroti et al. [50] provided the number of ME/CFS participants as a range. Hence, up to *n* = 343 pwME/CFS and *n* = 1,196 pwPCC are captured within this review.

The CCC was employed to ascertain ME/CFS cases in most studies [13, 46, 47, 49–52]. Three studies confirmed ME/CFS status with the ICC [12, 13, 48]. In addition to meeting the ICC, pwME/CFS were required to score less than 70 in the 36-Item Short-Form Health Survey (SF-36) Physical Functioning domain, return a Karnofsky Performance Status Index [60] score of less than 70 and spend at least 14 h per day in a reclined position in the study authored by Chang et al. [48]. PwPCC either directly fulfilled or had an illness presentation consistent with the WHO case definition in all studies [13, 53–59]. Additional eligibility criteria were applied by Ariza et al. [53] to categorise pwPCC into three subgroups based on the severity of their acute COVID-19 illness, including pwPCC who: (a) experienced mild illness (M-pwPCC); (b) were hospitalised (H-pwPCC); and (c) were admitted to the intensive care unit (ICU-pwPCC).

Sociodemographic characteristics were largely similar between the comparator groups across the included studies. Participants were typically middle-aged adults. The mean ages of the pwME/CFS and pwPCC ranged from 36.02 to 53 years (*x̄* = 47.56 years) and 35 to 53.24 years (*x̄* = 46.28 years), respectively. Medians of age were similarly within the middle-age bracket for pwME/CFS and pwPCC, ranging from 30.0 to 42.00 years (*M* = 36 years) and 37 to 47.00 years (*M* = 42 years), respectively. HCs were significantly younger than pwME/CFS in one study [52] and pwPCC in two studies [13, 53]. H-PwPCC and ICU-pwPCC were also significantly older than HCs and M-pwPCC in the study authored by Ariza et al. [53]. PwPCC were significantly older than pwME/CFS in the study authored by Weigel et al. [13]. Cai et al. [54] reported a significantly younger cohort of pwPCC when compared with HCs.

Among pwME/CFS, mean illness duration ranged from 7.47 to 21 years (*x̄* = 14.56 years). Ryabkova et al. [51] and Weigel et al. [13] reported median illness durations of 7.0 and 10.00 years (*M* = 8.5 years), respectively. Mean and median illness durations among pwPCC ranged from 0.73 to 2.00 years (*x̄* = 1.15 years) [53, 55] and 0.33 to 0.89 years (*M* = 0.63 years) [13, 56, 59], respectively. Ariza et al. [53] reported a significantly longer mean illness duration among M-pwPCC (*x̄* = 1.01 years) than H-pwPCC (*x̄* = 0.84 years) and ICU-pwPCC (*x̄* = 0.73 years).

On average, 81.2% and 63.2% of the pwME/CFS and pwPCC, respectively, were female or women. The distribution of females or women ranged from 51.1% to 100.0% and 43.3% to 81.8% among pwME/CFS and pwPCC, respectively. The PCC study cohort consisted of significantly more females or women when compared with HCs in two studies [54, 57]. Additionally, Ariza et al. [53] reported significantly more female HCs and M-pwPCC than H-pwPCC and ICU-pwPCC.

### HRQoL

#### PROMs

Eleven different PROMs were employed by the included studies. The most frequently employed PROMs were surveys derived from the Medical Outcomes Study (MOS) [61] — including the RAND-36, SF-36 and 12-Item Short-Form Health Survey (SF-12) [12, 13, 47–52, 56–58]. All but one of the nine studies capturing pwME/CFS as a comparator group employed MOS questionnaires [12, 13, 46–52]. Of these, one study [49] employed the RAND-36 and seven studies used the SF-36 [12, 13, 47, 48, 50–52]. MOS questionnaires were also used in three studies among pwPCC [56–58]. However, EuroQol questionnaires [62, 63] – including the EuroQol 5-Dimension 3-Level questionnaire (EQ-5D-3L), the EuroQol 5-Dimension 5-Level questionnaire (ED-5D-5L) and the EuroQol Visual Analogue Scale (EQ-VAS) – were the most frequently distributed PROMs among pwPCC and were used in four studies [53–55, 59].

HRQoL data was also captured via the Dr Bell’s Chronic Fatigue and Immune Dysfunction Syndrome (CFIDS) Disability Scale [64] (*n* = 1, 100.0% study among pwME/CFS and pwPCC [13]), Karnofsky Performance Status Index [60] (*n* = 1, 12.5% study among pwME/CFS [46]; *n* = 1, 100.0% study among pwME/CFS and pwPCC [13]), Sheehan Disability Scale [65] (*n* = 1, 14.3% study among pwPCC [58]), World Health Organization Disability Assessment Schedule version 2.0 (WHODAS 2.0) [66] (*n* = 1, 12.5% study among pwME/CFS [12]; *n* = 1, 14.3% study among pwPCC [53]; *n* = 1, 100.0% study among pwME/CFS and pwPCC [13]) and WHOQOL-BREF [67] (*n* = 1, 100.0% study among pwPCC [53]). Complete values and significance are provided for all publications reporting PROM scores in S3 to S10 Tables, Additional file 2.

#### PwME/CFS

##### Overall health status and longitudinal changes

Significantly poorer perceptions of overall health status were observed among pwME/CFS. Karnofsky Performance Status Index scores were significantly impaired when compared with HCs and ranged from 30% to 62% [46, 48]. Similarly, Weigel et al. [13] observed a significantly lower median Dr Bell’s CFIDS Disability Scale score of 40.0% among pwME/CFS when compared with HCs. As the only study to provide longitudinal comparisons of HRQoL with HCs, Cambras et al*.* [47] reported significantly reduced Global SF-36 scores among pwME/CFS at both the baseline and follow-up time points.

Cambras et al*.* [47] also observed sustained reductions in HRQoL among pwME/CFS when compared with HCs over time. Physical Functioning, Role Physical, Bodily Pain, General Health, Vitality and Social Functioning scores were significantly lower among pwME/CFS when compared with HCs at both the baseline and follow-up time points. Role Emotional scores were consistently comparable between pwME/CFS and HCs. Mental Health scores were significantly impaired among pwME/CFS at baseline but lost significance at follow-up. Omnibus analyses revealed no significant changes between the scores returned by pwME/CFS or HCs at the baseline and follow-up time points.

##### MOS questionnaires

Physical Functioning, Role Physical, Bodily Pain, General Health, Vitality and Social Functioning scores were significantly compromised among pwME/CFS in all studies comparing MOS questionnaire scores with HCs [12, 13, 47–52]. Despite being significantly younger than the HC cohort, pwME/CFS in the study authored by Strand et al. [52] returned comparable scores and patterns of illness impact across the HRQoL domains when compared with the other included studies. The most substantial impacts among pwME/CFS were consistently observed in Role Physical. Mean and median Role Physical scores ranged from 0.0 to 7.7 (*x̄* = 2.58) [12, 47–49, 52] and 0.0 to 18.75 (*M* = 9.38) [13, 51], respectively. Vitality (also known as Energy/Fatigue) scores were also considerably impaired among pwME/CFS, with means and medians ranging from 7.0 to 23.33 (*x̄* = 15.54) [12, 47–49, 52] and 6.25 to 10.0 (*M* = 8.13) [13, 51], respectively. Similar impacts were observed in Social Functioning and General Health. Chang et al*.* [48] reported a considerably low mean Social Functioning score of 4.4, which may be due to this study cohort experiencing severe illness. Mean Social Functioning scores otherwise ranged from 19.2 to 33.0 (*x̄* = 23.05) [12, 47, 49, 52] and both Ryabkova et al. [51] and Weigel et al. [13] returned a median Social Functioning score of 25.0. For General Health, mean and median scores ranged from 16.5 to 28.32 (*x̄* = 23.55) [12, 47–49, 52] and 25.00 to 30.0 (*M* = 27.5) [13, 51], respectively.

Scores typically ranged between approximately 30 and 50 for Physical Functioning (*x̄* = 27.76 [12, 47–49, 52] and *M* = 37.5 [13, 51], respectively) and Bodily Pain (*x̄* = 34.39 [12, 47–49, 52] and* M* = 59.5 [13, 51], respectively). Chang et al. [48] again observed a notably low mean Physical Functioning score of 13.3, likely due to the study cohort’s greater illness severity. Cambras et al. [47] also reported considerably lower mean Physical Functioning and Bodily Pain scores of 15.0 to 16.0 and 14.0 to 20.2, respectively, when compared with the other studies. Contrastingly, Ryabkova et al. [51] documented a substantially high median Bodily Pain score of 74.0, which may be due to the exclusion of pwME/CFS with comorbid fibromyalgia. This exclusion criterion may have selected for an illness presentation of ME/CFS characterised by less bodily pain and therefore higher Bodily Pain scores. Importantly, both studies authored by Cambras et al. [47] and Ryabkova et al. [51] had considerably small sample sizes of *n* = 10 and *n* = 11 pwME/CFS, respectively.

Role Emotional and Mental Health (also known as Emotional Wellbeing) were consistently among the highest of all the domain scores for pwME/CFS. Mean and median scores typically ranged between approximately 50 and 70 for Role Emotional (*x̄* = 62.61 [12, 47–49, 52] and *M* = 37.5 [13, 51]) and Mental Health (*x̄* = 61.21 [12, 47–49, 52] and *M* = 50.0 [13, 51], respectively). However, there were discrepancies in significance between pwME/CFS and HCs for these domains. Ryabkova et al. [51] reported significantly lower scores among pwME/CFS than HCs in both Role Emotional and Mental Health. In another three studies [12, 13, 48] — composed entirely or largely of pwME/CFS fulfilling the ICC — Role Emotional scores were significantly poorer when compared with HCs. Yet, scores in this domain were comparable between pwME/CFS and HCs in the remaining four studies [47, 49, 50, 52]. Fewer disparities were observed for Mental Health scores. Although Cambras et al. [47] reported a loss in significance at follow-up and Maroti et al. [50] documented no significant differences between pwME/CFS and HCs, Mental Health scores were significantly impaired among pwME/CFS when compared with HCs in most studies [12, 13, 48, 49, 51, 52].

Physical and Mental Component Summary scores were only reported by Maroti et al. [50]. While descriptive statistics were not provided, Maroti et al*.* [50] observed a significantly reduced Physical Component Summary score among pwME/CFS but no significant difference in the Mental Component Summary score.

##### WHODAS 2.0

All WHODAS 2.0 domains were significantly impaired among pwME/CFS when compared with HCs [12, 13]. The greatest impacts were recorded in Life Activities and Participation, with mean and median scores ranging from 50 to 60 and 60 to 80, respectively [12, 13]. Mean and median Cognition, Mobility and Getting Along scores ranged from approximately 40 to 50 [12, 13]. Self-Care was the least impacted of the six WHODAS 2.0 domains. Still, Self-Care scores were significantly elevated among pwME/CFS (*x̄* = 22.2, *M* = 30.00 [12, 13]) when compared with HCs.

#### PwPCC

##### Overall health status and longitudinal changes

Perceptions of overall health status were consistently poorer among pwPCC when compared with HCs. PwPCC returned a significantly impaired median Dr Bell’s CFIDS Disability Scale score of 40.0% [13]. Mean EQ-VAS scores among pwPCC ranged from 42.05 to 76.01 (*x̄* = 62.60) and were significantly lower than those of HCs in all studies [53–55, 59]. Similarly, Ariza et al. [53] reported significantly compromised EQ-5D-3L index values among pwPCC, with means ranging from 0.677 to 0.787. Mean Global WHODAS 2.0 scores also indicated significantly greater impairment among pwPCC when compared with HCs and ranged from 26.54 to 31.98 [53]. Surprisingly, the Global SF-12 score reported by Espinar-Herranz et al. [57] was not significantly lower when compared with HCs. However, the mean score returned by the HCs in this study [57] was below 40%, corresponding to “fair” health status.

Nehme et al. [58] observed impairments in the Global domain of the Sheehan Disability Scale among 95.6% of pwPCC (adjusted). However, the possible scores for this domain range from 0 to 30 [65] and the threshold for impairment was not defined by the authors [58]. Nehme et al. [58] also documented loss of productivity within the last week due to impairments in functioning among 46.9% of pwPCC (adjusted). Yet, it was unclear whether pwPCC differed significantly from HCs, as this study [58] included four comparator groups and only omnibus statistical test results were provided.

Although the present review captured two longitudinal studies among pwPCC, neither Cai et al. [54] nor Nehme et al. [58] compared the HRQoL of pwPCC with that of HCs over time. Of the 450 pwPCC examined by Cai et al. [54] at baseline, a subset of 105 participants were followed up at 12-months post-infection. However, this subset captured those who had recovered from PCC and no stratified analyses of HRQoL among recovered pwPCC compared with those with persistent PCC were reported. Additionally, while Nehme et al. [58] examined recovered COVID-19 survivors, recovered pwPCC and those with persistent PCC at seven- and 15-months post-infection, HRQoL data was only collected and compared at the final time point.

##### EuroQol questionnaires

All EQ-5D-5L domains were significantly impacted among pwPCC when compared with HCs [54, 55, 59]. Mean domain scores were only provided by Calvache-Mateo et al. [55]. Pain/Discomfort and Usual Activities returned the highest mean scores (*x̄* = 3.43 and *x̄* = 3.24, respectively). In the study authored by Seeley et al. [59], all pwPCC experienced at least slight problems in Usual Activities. Additionally, Seeley et al. [59] observed at least slight Pain/Discomfort impairments in 88% of pwPCC. Mobility and Self-Care were among the least impacted domains in the studies authored by Calvache-Mateo et al. [55] and Seeley et al. [59]. Cai et al. [54] observed the greatest impacts in Anxiety/Depression. Importantly, Cai et al. [54] only analysed HRQoL data from a total population sample of hospitalised COVID-19 survivors, whereas the study populations examined by Calvache-Mateo et al. [55] and Seeley et al. [59] were convenience samples consisting entirely or largely of pwPCC who had not been hospitalised. Nevertheless, the proportions of pwPCC experiencing limitations in Usual Activities, Mobility and Self-Care reported by Cai et al. [54] (being 3.24%, 2.36% and 1.80%, respectively) were notably low. However, the possible scores for these domains range from 1 to 5 [63] and the threshold for impairment in this study [54] was not defined.

##### MOS questionnaires

SF-36 data among pwPCC was limited and significance between pwPCC and HCs varied considerably across the included studies providing domain scores [13, 56]. Nehme et al. [58] provided the Physical and Mental Component Summary scores for the SF-12; however, significance between pwPCC and HCs was not specified. De Sousa et al. [56] reported notably higher scores in all SF-36 domains when compared with those observed by Weigel et al. [13]. While the pwPCC in the study authored by Weigel et al. [13] were significantly older than the HCs, age was controlled in the HRQoL analyses. Hence, the lower domain scores in this study can likely explained by over half of the PCC cohort fulfilling ME/CFS case criteria. De Sousa et al. [56] also observed a higher burden on Bodily Pain and General Health relative to the other domains—a finding that was not reproduced by Weigel et al. [13]. Nevertheless, all physical health domains were significantly impaired among pwPCC when compared with HCs in both studies. Vitality and Social Functioning were among the lowest scoring domains. However, while Weigel et al. [13] observed significantly lower scores in these domains among pwPCC than HCs, this finding was not mirrored by De Sousa et al. [56]. Role Emotional and Mental Health scores were among the least impacted domains but remained significantly impaired when compared with HCs in both studies (except for Mental Health in the study authored by De Sousa et al. [56]).

##### WHODAS 2.0

All WHODAS 2.0 domains were significantly impaired among pwPCC when compared with HCs [13, 53]. Life Activities and Participation were the most impacted domains. Mean Life Activities and Participation scores among M-pwPCC, H-pwPCC and ICU-pwPCC ranged from 27.69 to 38.08 (*x̄* = 31.55) and 28.94 to 34.28 (*x̄* = 31.05), respectively [53]. Weigel et al. [13] observed median Life Activities and Participation scores of 70.00 and 50.00, respectively. For Cognition, Mobility and Getting Along, mean scores ranged from 10 to 30 [53] and median scores ranged from 20 to 40 [13]. HCs returned comparable Getting Along scores with H-pwPCC and ICU-pwPCC, as well as comparable Self-Care scores with ICU-pwPCC [53]. Self-Care was the least impacted domain, with mean and median scores between approximately 0 and 10 [13, 53].

##### WHOQOL-BREF

Ariza et al. [53] reported significant impairments in the Physical domain of the WHOQOL-BREF among all PCC cohorts. The Physical domain was the most impacted, with mean scores of approximately 50 [53]. Mean scores for the remaining WHOQOL-BREF domains ranged from approximately 50 to 70. Psychological scores were significantly poorer among M-pwPCC and H-pwPCC when compared with HCs [53]. Environmental scores were lower than HCs for M-pwPCC only [53]. These findings may be explained by M-pwPCC and H-pwPCC having a significantly longer illness duration when compared with ICU-pwPCC. The trends observed did not appear to be associated with the significantly different age and sex distributions across the subgroups. Interestingly, all PCC cohorts returned comparable Social Relationships scores with HCs [53].

### PwME/CFS v pwPCC

Weigel et al. [13] reported no significant differences in the Dr Bell’s CFIDS Disability Scale, SF-36 or WHODAS 2.0 scores between pwME/CFS and pwPCC. The poorest HRQoL scores were observed in Role Physical, Vitality and Social Functioning for both cohorts [13]. Similarly, pwPCC and pwME/CFS returned the greatest functional impairments in Life Activities and Participation [13]. Role Emotional, Mental Health and Self-Care were the least impacted domains [13]. Although pwPCC were significantly older than pwME/CFS in this study, the comparable HRQoL domain scores between these two cohorts are likely due to over half of the PCC cohort fulfilling ME/CFS criteria. Nehme et al. [58] identified the presence of CFS among pwPCC and HCs but the case criteria were not specified and no stratified analyses for pwPCC fulfilling ME/CFS criteria were provided. Hence, it was not possible to compare the HRQoL scores returned by those with CFS and pwPCC without CFS captured in the study authored by Nehme et al. [58].

### Quality assessment

The quality assessment results for each study are presented in S11 Table, Additional file 1, with detailed justifications in S12 Table, Additional file 1. Ambiguity in the inclusion criteria (particularly regarding the presence of comorbidities), poor identification and mitigation of potential confounding factors, and limited information about the statistical methods used were the primary issues compromising study quality.

Differences in the characteristics of the participants and non-participants were not identified by any of the included studies. Only four studies employed stringent exclusion criteria regarding comorbidity [12, 13, 47, 57]. Specific comorbidities, typically neurological, autoimmune and psychiatric conditions, were excluded in five studies [49, 51, 53, 56, 59]. No information was provided on the participants’ comorbidity status in three studies [46, 48, 52]. Maroti et al. [50] did not exclude pwME/CFS who had comorbid conditions that were managed and not primarily responsible for their symptoms. Participants with comorbidity (including HCs) were captured within the study cohorts of the remaining five studies – all of which were among pwPCC [53–55, 58, 59]. Smoking was exclusionary in one study [47] but, in most studies [12, 13, 46, 48–52, 54, 56, 57, 59], eligibility based on smoking status was unclear. Current smokers were included in three studies among pwPCC [53, 55, 58].

All but three studies [12, 48, 49, 51, 52, 54, 56, 57, 59] did not adjust for confounders in HRQoL comparisons. Almost all of the included studies [12, 46–59] did not report the results of assumptions tests relevant to the statistical methods chosen. Nehme et al. [58] provided omnibus but not post-hoc test results for the study cohorts, limiting data analysis in the present review. Finally, most studies [12, 46, 48–52, 54–57, 59] did not report whether *p*-values had been adjusted for multiple comparisons.

## Discussion

This systematic review aimed to summarise and critically appraise the existing literature comparing HRQoL among pwME/CFS and pwPCC with HCs and analyse the patterns of HRQoL impact among these two illness cohorts. Importantly, this review consolidated HRQoL data collected via validated PROMs among ME/CFS and PCC cohorts meeting the most stringent diagnostic criteria currently available. To the authors’ knowledge, this is the first systematic review to capture and compare HRQoL among both pwME/CFS and pwPCC in tandem. The present review is also the first to systematically collate quantitative HRQoL data among pwPCC meeting the WHO case definition [23].

All aspects of HRQoL were consistently compromised among pwME/CFS and pwPCC when compared with HCs. PwME/CFS and pwPCC repeatedly returned significantly poorer scores than HCs in the Dr Bell’s CFIDS Disability Scale, Karnofsky Performance Status Index, EQ-VAS, EQ-5D-3L index and the Global SF-36 and WHODAS 2.0 domains, indicating worsened overall health status. Impaired physical health among pwME/CFS and pwPCC was evidenced through significant reductions in the Pain/Discomfort domain of the EuroQol questionnaires, the Mobility domains of the EuroQol questionnaires and the WHODAS 2.0, the Physical Functioning, Role Physical, Bodily Pain and General Health SF-36 domains, and the Physical Component Summary scores of the SF-36 and SF-12. Compromised scores were also returned by both illness cohorts in domains assessing one’s ability to perform typical daily activities, including the Usual Activities domain of the EuroQol questionnaires, SF-36 Vitality, and the Life Activities and Participation domains of the WHODAS 2.0. While direct comparisons between pwME/CFS and pwPCC were only available in one study [13], no statistically significant differences were observed in any measure of HRQoL. Hence, the findings of the present review foreground that pwME/CFS and pwPCC experience a comparable, profound level of disability despite the latter cohort having a considerably shorter illness duration. This recapitulates the overlaps in the lived experiences among pwME/CFS and pwPCC documented in qualitative studies [15, 34, 35, 68–70].

The poorest scores were returned for physical health domains (such as SF-36 Role Physical and the Pain/Discomfort domain of the EuroQol questionnaires) and domains assessing one’s ability to perform typical daily activities (including the Usual Activities domain of the EuroQol questionnaires, SF-36 Vitality, and the Life Activities and Participation domains of the WHODAS 2.0). Jason et al. [71] similarly documented substantial reductions in Role Physical and Vitality in a review of SF-36 scores among pwME/CFS. Both ME/CFS and PCC are associated with numerous, disabling symptoms, including cognitive dysfunction, extensive physical discomfort due to bodily pain, autonomic dysfunction, thermostatic disturbances and feelings of complete exhaustion [5, 7, 21, 35, 70, 72, 73]. Limitations on physical functioning are further compounded as symptoms are exacerbated upon exertion [5, 7, 21, 72, 73]. Symptom flares due to post-exertional malaise may result in prolonged periods of being bed-bound and requiring a low-stimulation environment with minimal time sitting upright [3, 5, 7, 72, 73]. Hence, the illness presentation of ME/CFS and PCC poses a considerable barrier to completing physical tasks associated with daily living, as evidenced by the findings of the present review.

While still significantly impaired when compared with HCs, domains corresponding to mental wellbeing and self-care activities tended to return the least impacted scores among both pwME/CFS and pwPCC. However, there were some discrepancies in mental health impacts across the included studies and the PROMs employed. As reliability statistics for the PROMs employed were only provided by Weigel et al. [13], it is not possible to definitively confirm whether reporting disparities may explain the apparent reductions in impact on the mental health domains across the included studies. However, the internal consistency statistics reported by Weigel et al. [13] confirm that the Mental Health and Role Emotional domains of the SF-36v2 returned sufficient reliability. Additionally, all items captured within the PROMs across the included studies, regardless of their assessment of physical or mental health, have been sufficiently validated in fulfilment of the present review’s eligibility criteria. As all corresponding items must be completed to calculate the domain scores, these could not have been biased by missing data. Hence, the reduced impairments in mental health domains when compared with physical health domains is a valid finding of the present review. This finding suggests that, while mental health is impacted among pwME/CFS and pwPCC, the primary source of disability for people living with these illnesses is physical.

Living with a chronic illnesses such as ME/CFS or PCC is associated with reductions in mental wellbeing due to loss of lifestyle, independence and identity, as well as uncertainty about the future of one’s health [15, 35, 68, 69]. As a result, anxiety and depression may occur as secondary comorbidities among people who acquire ME/CFS or PCC [3, 5, 23, 74]. Importantly, the substantial limitations on physical health observed in the present review reiterate that poor mental health outcomes among pwME/CFS and pwPCC are circumstantial and are not causative of either condition. This is supported by the extensive published evidence confirming that the pathology of these conditions is biological and not psychogenic in nature. Objective measures have identified disruptions to physiological processes, including impaired calcium ion channel function, endothelial dysfunction, coagulopathy, volumetric brain changes, small fibre neuropathy and mitochondrial dysfunction [16, 21, 29].

Although comparable patterns in illness impact were observed among pwME/CFS and pwPCC, the inconsistent use of PROMs across the included publications limited direct comparisons of HRQoL scores. EuroQol questionnaires were used exclusively in the studies among pwPCC, whereas MOS questionnaires were employed in all but one of the studies capturing pwME/CFS as a comparator group. The preferential use of EuroQol questionnaires in the studies among pwPCC may be due to the small number of survey items, which may be more suitable for studies with larger sample sizes [62, 63, 75], such as those examined by Ariza et al. [53] and Cai et al. [54]. Additionally, the consistent use of MOS questionnaires among pwME/CFS may be due to the role of the SF-36 domain score thresholds in case criteria for ME/CFS [71, 76, 77]. It is also worth noting that psychometric properties to support the validity of MOS questionnaires such as the SF-36 among PCC cohorts fulfilling the WHO case definition had not been published prior to the reliability statistics reported by Weigel et al. [13] in 2024.

In the only study to capture ME/CFS and PCC cohorts, Weigel et al. [13] reported no significant differences between the two groups in any HRQoL domain. Only three other studies among pwPCC employed PROMs that were used by studies among pwME/CFS. De Sousa et al. [56] documented considerably higher scores in all SF-36 domains among pwPCC when compared with those reported by Weigel et al. [13]. Similarly, although illness impacts relative to other domains were comparable with those observed among pwME/CFS, Ariza et al. [53] reported notably lower scores in all WHODAS 2.0 domains — particularly Life Activities and Participation — among all PCC subsets when compared with Weigel et al. [13]. Additionally, Ariza et al. [53] documented no significant differences between pwPCC and HCs in the Social Relationships domain of the WHOQOL-BREF and comparable WHODAS 2.0 Getting Along scores between HCs, H-pwPCC and ICU-pwPCC. De Sousa et al. [56] similarly reported no significant differences in SF-36 Social Functioning scores between pwPCC and HCs*.* However, these findings were not paralleled by Weigel et al. [13]. While statistical comparisons could not be conducted, the disparate domain scores returned by pwPCC reported by De Sousa et al. [56] and Ariza et al. [53] when compared with Weigel et al. [13] suggest that the HRQoL scores returned by pwPCC in the former two studies may be significantly higher than those of pwME/CFS.

These findings may be explained by illness duration and the poor specificity of existing PCC case criteria. Despite experiencing mild acute COVID-19 illness, M-pwPCC returned more significant differences in HRQoL when compared with HCs and scored the lowest in all HRQoL domains (except WHODAS 2.0 Self-Care) than H-pwPCC and ICU-pwPCC in the study authored by Ariza et al. [53]. A causal relationship between acute COVID-19 illness severity and the likelihood and severity of PCC has not yet been confirmed [21, 25, 78]. However, M-pwPCC had a significantly longer illness duration than the other two PCC cohorts in the study authored by Ariza et al. [53]. Among pwME/CFS, the fluidity of symptom presentation is greatest in the early stages of the illness and the likelihood of spontaneous recovery is highest in the first two years following illness onset [3, 5, 6, 73, 79]. Hence, pwPCC with longer illness durations may be less likely to recover from PCC and more likely to present with ME/CFS-like illness, potentially explaining the worsened HRQoL burdens among those who survived mild acute COVID-19 illness.

Contrastingly, despite having the shortest median illness duration of the PCC cohorts captured by the present review, the HRQoL domain scores returned by pwPCC in the study authored by Weigel et al. [13] had the greatest resemblance to those observed among pwME/CFS. It is important to acknowledge, however, that over half of the PCC cohort examined by Weigel et al. [13] fulfilled ME/CFS criteria. These findings are highlighted not to discount the debilitating burdens faced by pwPCC but rather to reiterate the importance of refining PCC diagnostic criteria to delineate illness subtypes in clinical practice and research. This is further exemplified by the EQ-VAS scores observed among pwPCC by Ariza et al. [53], Cai et al. [54] and Calvache-Mateo et al. [55]. Although all significantly impacted when compared with HCs, mean EQ-VAS scores ranged from 42.05 to 76.01 across the studies [53–55]. Yet, among pwME/CFS, all HRQoL domain scores appeared relatively comparable across the corresponding studies with the few outliers likely explained by illness severity or small sample size.

Reducing the heterogeneity of PCC study populations has marked importance for studies among pwPCC with ME/CFS-like illness. COVID-19 survivors who fulfil the broad, existing PCC case definitions but do not meet ME/CFS criteria may in fact be presenting with a different post-COVID-19 sequela. Including these COVID-19 survivors in PCC study cohorts can underestimate the debilitating symptoms and impacts on HRQoL faced by some pwPCC. This is evidenced by the findings of Kedor et al. [31], who identified that the symptom presentation and HRQoL of pwME/CFS was significantly different when compared with pwPCC without ME/CFS-like illness but not when compared with pwPCC presenting with ME/CFS-like illness. Legler et al. [80] also identified an increasing number of significant differences in symptom presentation and HRQoL over time between pwPCC with and without ME/CFS-like illness. While few significant differences were observed between the two PCC cohorts at the baseline time point, the findings of Legler et al. [80] further posit that PCC prognosis is dependent on the illness’ subtype. Hence, distinguishing PCC subtypes is vital to ensure timely implementation of the appropriate care pathways and interventions in practice, as well as the accurate documentation of illness burdens in research to guide appropriate care.

The shared impact patterns observed among pwME/CFS and pwPCC in this review nevertheless suggest the reproducibility of these illness impact trends using a standardised PROM. Harmonised HRQoL data collection among pwME/CFS and pwPCC should be prioritised in future epidemiological studies to compare the illness severity and burdens of PCC study cohorts with ME/CFS norms. This is essential to determine the generalisability of study results to pwPCC with ME/CFS-like illness. The consistent use of PROMs to monitor HRQoL among these cohorts over time should also be prioritised to identify predictors of illness trajectory among pwPCC, which will be vital to distinguish those at risk of long-term ME/CFS-like illness in clinical practice. Additionally, as many of the included studies provided limited information about the statistical methods chosen, uniform reporting of statistical methods and relevant assumptions would be advantageous in future observational studies.

Longitudinal data among pwME/CFS and pwPCC fulfilling the diagnostic criteria of interest is limited in the existing literature and was provided by only one study among pwME/CFS in the present review. Cambras et al. [47] observed that pwME/CFS maintained their significantly impaired scores when compared with HCs for all SF-36 domains except Role Emotional and Mental Health. These findings indicate that the HRQoL burdens experienced by pwME/CFS are not only profound but also persistent. This has important implications for Australian healthcare policies, which currently do not recognise the protracted impacts on functioning experienced by pwME/CFS [36, 38].

However, no other eligible studies have been published to corroborate the results observed by Cambras et al. [47]. Additionally, despite capturing two prospective panel studies, no longitudinal analyses of HRQoL among pwPCC when compared with HCs were reported by the publications in the present review. Further longitudinal research is paramount to define the potential post-infectious illness trajectories following acute COVID-19 illness, including their duration and progression. This is integral to inform developments in existing PCC case definitions to increase the definitions’ specificity and delineate illness subtypes. Early identification of PCC subtypes via refined diagnostic protocols is essential to ensure study populations in research are representative of the true illness cohorts and that research findings are generalisable. Expanding longitudinal research among such PCC subtypes must subsequently be prioritised to collect accurate, illness-specific prognostic data. This is essential to inform evidence-based healthcare policies and ensure care pathways are tailored to and align with the needs of PCC subtypes, including those with ME/CFS-like illness, to maximise health outcomes for COVID-19 survivors.

### Strengths and limitations

Despite debilitating symptoms and a reduced capacity to work [4, 5, 81–84], the functional impairments associated with ME/CFS and PCC remain poorly recognised in healthcare policies. Reformed healthcare policies that reflect the disabling nature of pwME/CFS and pwPCC are paramount, particularly in the Australian context, to meet the care and support needs of these cohorts [36–38]. Hence, the present review serves to guide healthcare policy reforms by foregrounding the debilitating impacts of ME/CFS and PCC on HRQoL. Importantly, the widespread limitations on HRQoL observed in both cohorts warrant access to multidisciplinary support services that are tailored to the unique illness presentation of ME/CFS and PCC. Additionally, the remarkable similarities in the illness experiences of these two cohorts observed in this review further portend the risk of long-term chronic illness after SARS-CoV-2 infection. This reiterates the continued need for infection control measures to reduce both the acute and chronic disease burden of COVID-19 on healthcare systems, as well as to deliver timely and multidisciplinary support to COVID-19 survivors.

The present review benefitted from the inclusion of multiple databases and detailed search terms, which were piloted and refined following preliminary searches. Additionally, as all PROMs employed were self-administered, none of the HRQoL data analysed herein was collected by proxy. To ensure that this review provided a comprehensive summary of the impacts of ME/CFS and PCC on HRQoL, no exclusionary criteria were imposed on the type of PROM employed except that the instrument must collect generalised or overall HRQoL data and be formally validated. The use of the most stringent diagnostic criteria (which is preferred in the absence of universally-available laboratory tests) to ascertain ME/CFS and PCC cases reduced the potential of selecting for participants with other medical conditions.

It should be noted, however, that the number of studies eligible for inclusion in the present review was small. Moreover, most of these studies were among convenience samples with relatively small cohort sizes. Large-scale patient-reported outcome data is scarce, particularly among pwME/CFS. Additionally, as convenience sampling may introduce volunteer bias, population-based investigations — such as analyses of data linked to illness registries — are required to determine the HRQoL impacts experienced by the true population of pwME/CFS and pwPCC. Furthermore, most studies included in this review were based in Europe and participants were predominantly female and middle-aged. Therefore, the existing literature likely does not reflect the impairments in HRQoL experienced by pwME/CFS and pwPCC internationally or from marginalised populations. Improved collection of HRQoL data among pwME/CFS and pwPCC belonging to these populations must be prioritised to inform nation-specific healthcare policies and tailored approaches to care. Future studies, particularly those among pwPCC, should also control for comorbidity to define the HRQoL limitations directly attributable to these conditions to inform care pathways.

A meta-analysis was not possible in the context of the present review due to the heterogeneity of HRQoL data. However, a future meta-analysis may be warranted to identify PROM thresholds to aid the development of PCC case definitions. Due to the review’s inclusion criteria, studies investigating symptoms or impairments among COVID-19 survivors who were not specifically identified as having PCC were not captured. Nevertheless, the use of PCC-specific terms, as well as requiring pwPCC to have an illness presentation consistent with the WHO case definition, ensured that the impacts on patient-reported outcomes observed were directly attributable to ongoing, post-acute COVID-19-related illness.

Finally, many of the studies in the present review collected HRQoL data as a component of a larger suite of assessments including clinical and laboratory-based tests. While these study designs may introduce volunteer bias, exclusion of these studies would have prohibited a comprehensive examination of HRQoL among pwME/CFS and pwPCC. Additionally, the requirement of HRQoL data to be a primary outcome measure served to mitigate this potential for volunteer bias. It is worth noting, however, that routinely collected HRQoL data reported as a secondary outcome is not captured in the present review but appears consistent with the review’s findings in other clinical and laboratory-based studies [16, 17, 85, 86].

## Conclusion

The present systematic review served to consolidate the existing literature comparing the HRQoL of pwME/CFS and pwPCC with HCs. Consequently, this review sought to highlight and compare the HRQoL burdens faced by pwME/CFS and pwPCC to inform healthcare policies and care pathways. PwME/CFS and pwPCC experience similar, disabling impacts on HRQoL. All HRQoL domains were significantly reduced among pwME/CFS and pwPCC when compared with HCs. Shared impact patterns were observed between the two illness cohorts. Profound impairments were consistently observed in self-perceptions of overall health status, physical health domains and ability to perform daily activities. Although only provided in one study, there were no significant differences in direct comparisons of HRQoL outcomes between pwME/CFS and pwPCC. The findings of the present review emphasise the remarkable overlaps in disability among pwME/CFS and pwPCC, as well as the need for healthcare policy reform to facilitate access to multidisciplinary, person-centred care and support services for both cohorts. Such supports are integral to optimise health outcomes for pwME/CFS and pwPCC in the current absence of a curative therapy. The identification of PCC subtypes via refined diagnostic criteria in clinical practice and longitudinal research is vital to guide appropriate, illness-specific care.

## Supplementary Information

Additional file 1

Additional file 2

## Data Availability

All data synthesised from the eligible publications captured by this systematic review are published in the present manuscript. Table 2 contains all study and participant characteristics extracted from the eligible publications. The extracted PROM data from the included studies are presented in S3 to S10 Tables, Additional file 2. S2 Table, Additional file 1 outlines the complete search strategy for each database.

## Abbreviations

95% CI: 95% confidence interval
ANOVA: Analysis of variance
BMI: Body mass index
C: Consensus
CCC: Canadian Consensus Criteria
CFIDS: Chronic Fatigue and Immune Dysfunction Syndrome
CINAHL: Cumulative Index to Nursing and Allied Health Literature
EQ-5D-3L: EuroQol 5-Dimension 3-Level questionnaire
EQ-5D-5L: EuroQol 5-Dimension 5-Level questionnaire
EQ-VAS: EuroQol Visual Analogue Scale
HC: Healthy control
HRQoL or HRQL: Health-related quality of life
ICC: International Consensus Criteria
ICU: Intensive care unit
JBI: Joanna Briggs Institute
M: Median
MANCOVA: Multivariate analysis of covariance
ME/CFS: Myalgic Encephalomyelitis/Chronic Fatigue Syndrome
MOS: Medical Outcomes Study
NA: Not applicable
NCNED: National Centre for Neuroimmunology and Emerging Diseases
NR: Not reported
PASC: Post-Acute Sequelae of COVID(-19)
PCC: Post COVID-19 Condition
PRISMA: Preferred Reporting Items for Systematic Reviews and Meta-Analyses
PROM: Patient-reported outcome measure
PROSPERO: International Prospective Register of Systematic Reviews
PwME/CFS: People with Myalgic Encephalomyelitis/Chronic Fatigue Syndrome
PwPCC: People with Post COVID-19 Condition
Q1–Q3: Quartile 1 to quartile 3
SARS-CoV-2: Severe Acute Respiratory Syndrome Coronavirus-2
SF-12: 12-Item Short-Form Health Survey
SF-36: 36-Item Short-Form Health Survey
SPSS: Statistical Package for the Social Sciences
WHO: World Health Organization
WHODAS 2.0: World Health Organization Disability Assessment Schedule version 2.0
