# Supplementary material for: Health-related quality of life in Myalgic Encephalomyelitis/Chronic Fatigue Syndrome and Post COVID-19 Condition: a systematic review
Source: J Transl Med. 2025 Mar 13;23:318. doi: 10.1186/s12967-025-06131-z (PMC11905571; doi:10.1186/s12967-025-06131-z)
Supplement: Supplementary file 1 — Additional file 1 [file 12967_2025_6131_MOESM1_ESM.docx]

# Supplementary information tables (Additional file 1)

**Article title:** Health-related quality of life in Myalgic Encephalomyelitis/Chronic Fatigue Syndrome and Post COVID-19 Condition: A systematic review

**Journal name:** Journal of Translational Research

**Author names:** *Breanna Weigel^1,2,3^, Maira Inderyas^1,2^, Natalie Eaton-Fitch^1,2^, Kiran Thapaliya^1,2^ & Sonya Marshall-Gradisnik^1,2^

**Affiliations:**

^1^ National Centre for Neuroimmunology and Emerging Diseases, Griffith University, Gold Coast, Queensland, Australia 4222

^2^ Consortium Health International for Myalgic Encephalomyelitis, Griffith University, Gold Coast, Queensland, Australia 4222

^3^ School of Pharmacy and Medical Sciences, Griffith University, Gold Coast, Queensland, Australia 4222

***Corresponding author:**

Miss Breanna Weigel

Email: [ncned@griffith.edu.au](mailto:ncned@griffith.edu.au)

**S1 Table. PRISMA 2020 checklist.**

| **Section and Topic** | **Item #** | **Checklist item** | **Location where item is reported** |
| --- | --- | --- | --- |
| **TITLE** | | |  |
| Title | 1 | Identify the report as a systematic review. | 1 |
| **ABSTRACT** | | |  |
| Abstract | 2 | See the PRISMA 2020 for Abstracts checklist. | 2 |
| **INTRODUCTION** | | |  |
| Rationale | 3 | Describe the rationale for the review in the context of existing knowledge. | 3 – 5 |
| Objectives | 4 | Provide an explicit statement of the objective(s) or question(s) the review addresses. | 4 – 5 |
| **METHODS** | | |  |
| Eligibility criteria | 5 | Specify the inclusion and exclusion criteria for the review and how studies were grouped for the syntheses. | 6 – 7 |
| Information sources | 6 | Specify all databases, registers, websites, organisations, reference lists and other sources searched or consulted to identify studies. Specify the date when each source was last searched or consulted. | 5 |
| Search strategy | 7 | Present the full search strategies for all databases, registers and websites, including any filters and limits used. | 6 |
| Selection process | 8 | Specify the methods used to decide whether a study met the inclusion criteria of the review, including how many reviewers screened each record and each report retrieved, whether they worked independently, and if applicable, details of automation tools used in the process. | 6 – 7 |
| Data collection process | 9 | Specify the methods used to collect data from reports, including how many reviewers collected data from each report, whether they worked independently, any processes for obtaining or confirming data from study investigators, and if applicable, details of automation tools used in the process. | 7 |
| Data items | 10a | List and define all outcomes for which data were sought. Specify whether all results that were compatible with each outcome domain in each study were sought (e.g. for all measures, time points, analyses), and if not, the methods used to decide which results to collect. | 7 |
|  | 10b | List and define all other variables for which data were sought (e.g. participant and intervention characteristics, funding sources). Describe any assumptions made about any missing or unclear information. | 7 |
| Study risk of bias assessment | 11 | Specify the methods used to assess risk of bias in the included studies, including details of the tool(s) used, how many reviewers assessed each study and whether they worked independently, and if applicable, details of automation tools used in the process. | 8 |
| Effect measures | 12 | Specify for each outcome the effect measure(s) (e.g. risk ratio, mean difference) used in the synthesis or presentation of results. | 7 |

(continued)

**S1 Table. PRISMA 2020 checklist (continued).**

| **Section and Topic** | **Item #** | **Checklist item** | **Location where item is reported** |
| --- | --- | --- | --- |
| Synthesis methods | 13a | Describe the processes used to decide which studies were eligible for each synthesis (e.g. tabulating the study intervention characteristics and comparing against the planned groups for each synthesis (item #5)). | 7 |
|  | 13b | Describe any methods required to prepare the data for presentation or synthesis, such as handling of missing summary statistics, or data conversions. | NA |
|  | 13c | Describe any methods used to tabulate or visually display results of individual studies and syntheses. | NA |
|  | 13d | Describe any methods used to synthesize results and provide a rationale for the choice(s). If meta-analysis was performed, describe the model(s), method(s) to identify the presence and extent of statistical heterogeneity, and software package(s) used. | NA |
|  | 13e | Describe any methods used to explore possible causes of heterogeneity among study results (e.g. subgroup analysis, meta-regression). | NA |
|  | 13f | Describe any sensitivity analyses conducted to assess robustness of the synthesized results. | NA |
| Reporting bias assessment | 14 | Describe any methods used to assess risk of bias due to missing results in a synthesis (arising from reporting biases). | NA |
| Certainty assessment | 15 | Describe any methods used to assess certainty (or confidence) in the body of evidence for an outcome. | NA |
| **RESULTS** | | |  |
| Study selection | 16a | Describe the results of the search and selection process, from the number of records identified in the search to the number of studies included in the review, ideally using a flow diagram. | 8 – 9 |
|  | 16b | Cite studies that might appear to meet the inclusion criteria, but which were excluded, and explain why they were excluded. | NA |
| Study characteristics | 17 | Cite each included study and present its characteristics. | 8 – 11 |
| Risk of bias in studies | 18 | Present assessments of risk of bias for each included study. | S11 Table (page 18) |
| Results of individual studies | 19 | For all outcomes, present, for each study: (a) summary statistics for each group (where appropriate) and (b) an effect estimate and its precision (e.g. confidence/credible interval), ideally using structured tables or plots. | 11 – 17 |
| Results of syntheses | 20a | For each synthesis, briefly summarise the characteristics and risk of bias among contributing studies. | 8 – 18 |
|  | 20b | Present results of all statistical syntheses conducted. If meta-analysis was done, present for each the summary estimate and its precision (e.g. confidence/credible interval) and measures of statistical heterogeneity. If comparing groups, describe the direction of the effect. | NA |
|  | 20c | Present results of all investigations of possible causes of heterogeneity among study results. | NA |
|  | 20d | Present results of all sensitivity analyses conducted to assess the robustness of the synthesized results. | NA |

(continued)

## S1 Table. PRISMA 2020 checklist (continued).

| **Section and Topic** | **Item #** | **Checklist item** | **Location where item is reported** |
| --- | --- | --- | --- |
| Reporting biases | 21 | Present assessments of risk of bias due to missing results (arising from reporting biases) for each synthesis assessed. | 18 |
| Certainty of evidence | 22 | Present assessments of certainty (or confidence) in the body of evidence for each outcome assessed. | NA |
| **DISCUSSION** | | |  |
| Discussion | 23a | Provide a general interpretation of the results in the context of other evidence. | 19 – 23 |
|  | 23b | Discuss any limitations of the evidence included in the review. | 24 |
|  | 23c | Discuss any limitations of the review processes used. | 24 – 25 |
|  | 23d | Discuss implications of the results for practice, policy, and future research. | 21 – 24 |
| **OTHER INFORMATION** | | |  |
| Registration and protocol | 24a | Provide registration information for the review, including register name and registration number, or state that the review was not registered. | 5 |
|  | 24b | Indicate where the review protocol can be accessed, or state that a protocol was not prepared. | NA |
|  | 24c | Describe and explain any amendments to information provided at registration or in the protocol. | NA |
| Support | 25 | Describe sources of financial or non-financial support for the review, and the role of the funders or sponsors in the review. | 34 – 35 |
| Competing interests | 26 | Declare any competing interests of review authors. | 34 |
| Availability of data, code and other materials | 27 | Report which of the following are publicly available and where they can be found: template data collection forms; data extracted from included studies; data used for all analyses; analytic code; any other materials used in the review. | 34 |

Retrieved from Page *et al.* [42]. Abbreviations: *NA* Not applicable; *PRISMA* Preferred Reporting Items for Systematic Reviews and Meta-Analyses.

## S2 Table. Search strategy employed in the systematic searching of each database.

| **Database** | **Keywords** | **Search #** | **Search query** |
| --- | --- | --- | --- |
| **CINAHL** | **ME/CFS** | 1 | Limited to ‘Humans’: (MH “Fatigue Syndrome, Chronic”) |
|  |  | 2 | Limited to ‘Humans’: ((TI “Chronic Fatigue Syndrome” OR AB “Chronic Fatigue Syndrome”) OR (TI “Chronic Fatigue Syndromes” OR AB “Chronic Fatigue Syndromes”) OR (TI “Fatigue Syndrome, Chronic” OR AB “Fatigue Syndrome, Chronic”) OR (TI “Fatigue Syndromes, Chronic” OR AB “Fatigue Syndromes, Chronic”) OR (TI “Syndrome, Chronic Fatigue” OR AB “Syndrome, Chronic Fatigue”) OR (TI “Syndrome of Chronic Fatigue” OR AB “Syndrome of Chronic Fatigue”) OR (TI “Chronic Fatigue Disorder” OR AB “Chronic Fatigue Disorder”) OR (TI “Chronic Fatigue Disorders” OR AB “Chronic Fatigue Disorders”) OR (TI “Fatigue Disorder, Chronic” OR AB “Fatigue Disorder, Chronic”) OR (TI “Fatigue Disorders, Chronic” OR AB “Fatigue Disorders, Chronic”) OR (TI “Disorders, Chronic Fatigue” OR AB “Disorders, Chronic Fatigue”) OR (TI “Chronic Fatigue and Immune Dysfunction Syndrome” OR AB “Chronic Fatigue and Immune Dysfunction Syndrome”) OR (TI “Chronic Fatigue Fibromyalgia Syndrome” OR AB “Chronic Fatigue Fibromyalgia Syndrome”) OR (TI “Chronic Fatigue-Fibromyalgia Syndrome” OR AB “Chronic Fatigue-Fibromyalgia Syndrome”) OR (TI “Chronic Fatigue Fibromyalgia Syndromes” OR AB “Chronic Fatigue Fibromyalgia Syndromes”) OR (TI “Chronic Fatigue-Fibromyalgia Syndromes” OR AB “Chronic Fatigue-Fibromyalgia Syndromes”) OR (TI “Fatigue Fibromyalgia Syndrome, Chronic” OR AB “Fatigue Fibromyalgia Syndrome, Chronic”) OR (TI “Fatigue-Fibromyalgia Syndrome, Chronic” OR AB “Fatigue-Fibromyalgia Syndrome, Chronic”) OR (TI “Fatigue Fibromyalgia Syndromes, Chronic” OR AB “Fatigue Fibromyalgia Syndromes, Chronic”) OR (TI “Fatigue-Fibromyalgia Syndromes, Chronic” OR AB “Fatigue-Fibromyalgia Syndromes, Chronic”) OR (TI “Syndrome, Chronic Fatigue Fibromyalgia” OR AB “Syndrome, Chronic Fatigue Fibromyalgia”) OR (TI “Syndrome, Chronic Fatigue-Fibromyalgia” OR AB “Syndrome, Chronic Fatigue-Fibromyalgia”) OR (TI “Syndromes, Chronic Fatigue Fibromyalgia” OR AB “Syndromes, Chronic Fatigue Fibromyalgia”) OR (TI “Syndromes, Chronic Fatigue-Fibromyalgia” OR AB “Syndromes, Chronic Fatigue-Fibromyalgia”) OR (TI “Myalgic Encephalomyelitis” OR AB “Myalgic Encephalomyelitis”) OR (TI “Encephalomyelitis, Myalgic” OR AB “Encephalomyelitis, Myalgic”) OR (TI “Systemic Exertion Intolerance Disease” OR AB “Systemic Exertion Intolerance Disease”) OR (TI “Postviral Fatigue Syndrome” OR AB “Postviral Fatigue Syndrome”) OR (TI “Postviral Fatigue Syndromes” OR AB “Postviral Fatigue Syndromes”) OR (TI “Post Viral Fatigue Syndrome” OR AB “Post Viral Fatigue Syndrome”) OR (TI “Post Viral Fatigue Syndromes” OR AB “Post Viral Fatigue Syndromes”) OR (TI “Post-Viral Fatigue Syndrome” OR AB “Post-Viral Fatigue Syndrome”) OR (TI “Post-Viral Fatigue Syndromes” OR AB “Post-Viral Fatigue Syndromes”) OR (TI “Fatigue Syndrome, Postviral” OR AB “Fatigue Syndrome, Postviral”) OR (TI “Fatigue Syndromes, Postviral” OR AB “Fatigue Syndromes, Postviral”) OR (TI “Fatigue Syndrome, Post Viral” OR AB “Fatigue Syndrome, Post Viral”) OR (TI “Fatigue Syndromes, Post Viral” OR AB “Fatigue Syndromes, Post Viral”) OR (TI “Fatigue Syndrome, Post-Viral” OR AB “Fatigue Syndrome, Post-Viral”) OR (TI “Fatigue Syndromes, Post-Viral” OR AB “Fatigue Syndromes, Post-Viral”) OR (TI “Syndrome, Postviral Fatigue” OR AB “Syndrome, Postviral Fatigue”) OR (TI “Syndromes, Postviral Fatigue” OR AB “Syndromes, Postviral Fatigue”) OR (TI “Syndrome, Post Viral Fatigue” OR AB “Syndrome, Post Viral Fatigue”) OR (TI “Syndromes, Post Viral Fatigue” OR AB “Syndromes, Post Viral Fatigue”) OR (TI “Syndrome, Post-Viral Fatigue” OR AB “Syndrome, Post-Viral Fatigue”) OR (TI “Syndromes, Post-Viral Fatigue” OR AB “Syndromes, Post-Viral Fatigue”) OR (TI |

(continued)

**S2 Table. Search strategy employed in the systematic searching of each database (continued).**

| **Database** | **Keywords** | **Search #** | **Search query** |
| --- | --- | --- | --- |
|  |  |  | “Infectious Mononucleosis Like Syndrome, Chronic” OR AB “Infectious Mononucleosis Like Syndrome, Chronic”) OR (TI “Infectious Mononucleosis-Like Syndrome, Chronic” OR AB “Infectious Mononucleosis-Like Syndrome, Chronic”) OR (TI “Royal Free Disease” OR AB “Royal Free Disease”) OR (TI “Akureyri Disease” OR AB “Akureyri Disease”) OR (TI “Epidemic Neuromyasthenia” OR AB “Epidemic Neuromyasthenia”) OR (TI “Iceland Disease” OR AB “Iceland Disease”)) |
|  |  | 3 | #1 OR #2 |
|  | **PCC** | 4 | Limited to ‘Humans’: (MH “Post-Acute COVID-19 Syndrome”) |
|  |  | 5 | Limited to ‘Humans’: ((TI “Post COVID Condition” OR AB “Post COVID Condition”) OR (TI “Post COVID Conditions” OR AB “Post COVID Conditions”) OR (TI “Post-COVID Condition” OR AB “Post-COVID Condition”) OR (TI “Post-COVID Conditions” OR AB “Post-COVID Conditions”) OR (TI “Post COVID-19 Condition” OR AB “Post COVID-19 Condition”) OR (TI “Post COVID-19 Conditions” OR AB “Post COVID-19 Conditions”) OR (TI “Post-COVID-19 Condition” OR AB “Post-COVID-19 Condition”) OR (TI “Post-COVID-19 Conditions” OR AB “Post-COVID-19 Conditions”) OR (TI “Post COVID 19 Condition” OR AB “Post COVID 19 Condition”) OR (TI “Post COVID 19 Conditions” OR AB “Post COVID 19 Conditions”) OR (TI “Post-COVID 19 Condition” OR AB “Post-COVID 19 Condition”) OR (TI “Post-COVID 19 Conditions” OR AB “Post-COVID 19 Conditions”) OR (TI “Post COVID19 Condition” OR AB “Post COVID19 Condition”) OR (TI “Post COVID19 Conditions” OR AB “Post COVID19 Conditions”) OR (TI “Post-COVID19 Condition” OR AB “Post-COVID19 Condition”) OR (TI “Post-COVID19 Conditions” OR AB “Post-COVID19 Conditions”) OR (TI “Post COVID Syndrome” OR AB “Post COVID Syndrome”) OR (TI “Post-COVID Syndrome” OR AB “Post-COVID Syndrome”) OR (TI “Post COVID-19 Syndrome” OR AB “Post COVID-19 Syndrome”) OR (TI “Post-COVID-19 Syndrome” OR AB “Post-COVID-19 Syndrome”) OR (TI “Post COVID 19 Syndrome” OR AB “Post COVID 19 Syndrome”) OR (TI “Post-COVID 19 Syndrome” OR AB “Post-COVID 19 Syndrome”) OR (TI “Post COVID19 Syndrome” OR AB “Post COVID19 Syndrome”) OR (TI “Post-COVID19 Syndrome” OR AB “Post-COVID19 Syndrome”) OR (TI “Post COVID Fatigue” OR AB “Post COVID Fatigue”) OR (TI “Post-COVID Fatigue” OR AB “Post-COVID Fatigue”) OR (TI “Post COVID-19 Fatigue” OR AB “Post COVID-19 Fatigue”) OR (TI “Post-COVID-19 Fatigue” OR AB “Post-COVID-19 Fatigue”) OR (TI “Post COVID 19 Fatigue” OR AB “Post COVID 19 Fatigue”) OR (TI “Post-COVID 19 Fatigue” OR AB “Post-COVID 19 Fatigue”) OR (TI “Post COVID19 Fatigue” OR AB “Post COVID19 Fatigue”) OR (TI “Post-COVID19 Fatigue” OR AB “Post-COVID19 Fatigue”) OR (TI “Post COVID Neurological Syndrome” OR AB “Post COVID Neurological Syndrome”) OR (TI “Post-COVID Neurological Syndrome” OR AB “Post-COVID Neurological Syndrome”) OR (TI “Post COVID-19 Neurological Syndrome” OR AB “Post COVID-19 Neurological Syndrome”) OR (TI “Post-COVID-19 Neurological Syndrome” OR AB “Post-COVID-19 Neurological Syndrome”) OR (TI “Post COVID 19 Neurological Syndrome” OR AB “Post COVID 19 Neurological Syndrome”) OR (TI “Post-COVID 19 Neurological Syndrome” OR AB “Post-COVID 19 Neurological Syndrome”) OR (TI “Post COVID19 Neurological Syndrome” OR AB “Post COVID19 Neurological Syndrome”) OR (TI “Post-COVID19 Neurological Syndrome” OR AB “Post-COVID19 Neurological Syndrome”) OR (TI “Post COVID Impairment” OR AB “Post COVID Impairment”) OR (TI “Post-COVID Impairment” OR AB “Post-COVID Impairment”) OR (TI “Post COVID-19 Impairment” OR AB “Post COVID-19 |

(continued)

**S2 Table. Search strategy employed in the systematic searching of each database (continued).**

| **Database** | **Keywords** | **Search #** | **Search query** |
| --- | --- | --- | --- |
|  |  |  | Impairment”) OR (TI “Post-COVID-19 Impairment” OR AB “Post-COVID-19 Impairment”) OR (TI “Post COVID 19 Impairment” OR AB “Post COVID 19 Impairment”) OR (TI “Post-COVID 19 Impairment” OR AB “Post-COVID 19 Impairment”) OR (TI “Post COVID19 Impairment” OR AB “Post COVID19 Impairment”) OR (TI “Post-COVID19 Impairment” OR AB “Post-COVID19 Impairment”) OR (TI “PASC” OR AB “PASC”) OR (TI “Post Acute Sequelae of COVID” OR AB “Post Acute Sequelae of COVID”) OR (TI “Post-Acute Sequelae of COVID” OR AB “Post-Acute Sequelae of COVID”) OR (TI “Post Acute Sequelae of COVID-19” OR AB “Post Acute Sequelae of COVID-19”) OR (TI “Post-Acute Sequelae of COVID-19” OR AB “Post-Acute Sequelae of COVID-19”) OR (TI “Post Acute Sequelae of COVID 19” OR AB “Post Acute Sequelae of COVID 19”) OR (TI “Post-Acute Sequelae of COVID 19” OR AB “Post-Acute Sequelae of COVID 19”) OR (TI “Post Acute Sequelae of COVID19” OR AB “Post Acute Sequelae of COVID19”) OR (TI “Post-Acute Sequelae of COVID19” OR AB “Post-Acute Sequelae of COVID19”) OR (TI “COVID Post Acute Sequelae” OR AB “COVID Post Acute Sequelae”) OR (TI “COVID Post-Acute Sequelae” OR AB “COVID Post-Acute Sequelae”) OR (TI “COVID-19 Post Acute Sequelae” OR AB “COVID-19 Post Acute Sequelae”) OR (TI “COVID-19 Post-Acute Sequelae” OR AB “COVID-19 Post-Acute Sequelae”) OR (TI “COVID 19 Post Acute Sequelae” OR AB “COVID 19 Post Acute Sequelae”) OR (TI “COVID 19 Post-Acute Sequelae” OR AB “COVID 19 Post-Acute Sequelae”) OR (TI “COVID19 Post Acute Sequelae” OR AB “COVID19 Post Acute Sequelae”) OR (TI “COVID19 Post-Acute Sequelae” OR AB “COVID19 Post-Acute Sequelae”) OR (TI “Post Acute Sequelae of SARS-CoV-2” OR AB “Post Acute Sequelae of SARS-CoV-2”) OR (TI “Post-Acute Sequelae of SARS-CoV-2” OR AB “Post-Acute Sequelae of SARS-CoV-2”) OR (TI “Post Acute Sequelae of SARS CoV 2” OR AB “Post Acute Sequelae of SARS CoV 2”) OR (TI “Post-Acute Sequelae of SARS CoV 2” OR AB “Post-Acute Sequelae of SARS CoV 2”) OR (TI “Post Acute COVID Syndrome” OR AB “Post Acute COVID Syndrome”) OR (TI “Post Acute COVID Syndromes” OR AB “Post Acute COVID Syndromes”) OR (TI “Post-Acute COVID Syndrome” OR AB “Post-Acute COVID Syndrome”) OR (TI “Post-Acute COVID Syndromes” OR AB “Post-Acute COVID Syndromes”) OR (TI “Post Acute COVID-19 Syndrome” OR AB “Post Acute COVID-19 Syndrome”) OR (TI “Post Acute COVID-19 Syndromes” OR AB “Post Acute COVID-19 Syndromes”) OR (TI “Post-Acute COVID-19 Syndrome” OR AB “Post-Acute COVID-19 Syndrome”) OR (TI “Post-Acute COVID-19 Syndromes” OR AB “Post-Acute COVID-19 Syndromes”) OR (TI “Post Acute COVID 19 Syndrome” OR AB “Post Acute COVID 19 Syndrome”) OR (TI “Post Acute COVID 19 Syndromes” OR AB “Post Acute COVID 19 Syndromes”) OR (TI “Post-Acute COVID 19 Syndrome” OR AB “Post-Acute COVID 19 Syndrome”) OR (TI “Post-Acute COVID 19 Syndromes” OR AB “Post-Acute COVID 19 Syndromes”) OR (TI “Post Acute COVID19 Syndrome” OR AB “Post Acute COVID19 Syndrome”) OR (TI “Post Acute COVID19 Syndromes” OR AB “Post Acute COVID19 Syndromes”) OR (TI “Post-Acute COVID19 Syndrome” OR AB “Post-Acute COVID19 Syndrome”) OR (TI “Post-Acute COVID19 Syndromes” OR AB “Post-Acute COVID19 Syndromes”) OR (TI “COVID Syndrome, Post Acute” OR AB “COVID Syndrome, Post Acute”) OR (TI “COVID Syndrome, Post-Acute” OR AB “COVID Syndrome, Post-Acute”) OR (TI “COVID-19 Syndrome, Post Acute” OR AB “COVID-19 Syndrome, Post Acute”) OR (TI “COVID-19 Syndrome, Post-Acute” OR AB “COVID-19 Syndrome, Post-Acute”) OR (TI “COVID 19 Syndrome, Post Acute” OR AB “COVID 19 Syndrome, Post Acute”) OR (TI “COVID 19 |

(continued)

**S2 Table. Search strategy employed in the systematic searching of each database (continued).**

| **Database** | **Keywords** | **Search #** | **Search query** |
| --- | --- | --- | --- |
|  |  |  | Syndrome, Post-Acute” OR AB “COVID 19 Syndrome, Post-Acute”) OR (TI “COVID19 Syndrome, Post Acute” OR AB “COVID19 Syndrome, Post Acute”) OR (TI “COVID19 Syndrome, Post-Acute” OR AB “COVID19 Syndrome, Post-Acute”) OR (TI “Post Acute COVID Fatigue” OR AB “Post Acute COVID Fatigue”) OR (TI “Post-Acute COVID Fatigue” OR AB “Post-Acute COVID Fatigue”) OR (TI “Post Acute COVID-19 Fatigue” OR AB “Post Acute COVID-19 Fatigue”) OR (TI “Post-Acute COVID-19 Fatigue” OR AB “Post-Acute COVID-19 Fatigue”) OR (TI “Post Acute COVID 19 Fatigue” OR AB “Post Acute COVID 19 Fatigue”) OR (TI “Post-Acute COVID 19 Fatigue” OR AB “Post-Acute COVID 19 Fatigue”) OR (TI “Post Acute COVID19 Fatigue” OR AB “Post Acute COVID19 Fatigue”) OR (TI “Post-Acute COVID19 Fatigue” OR AB “Post-Acute COVID19 Fatigue”) OR (TI “Post Acute COVID Neurological Syndrome” OR AB “Post Acute COVID Neurological Syndrome”) OR (TI “Post-Acute COVID Neurological Syndrome” OR AB “Post-Acute COVID Neurological Syndrome”) OR (TI “Post Acute COVID-19 Neurological Syndrome” OR AB “Post Acute COVID-19 Neurological Syndrome”) OR (TI “Post-Acute COVID-19 Neurological Syndrome” OR AB “Post-Acute COVID-19 Neurological Syndrome”) OR (TI “Post Acute COVID 19 Neurological Syndrome” OR AB “Post Acute COVID 19 Neurological Syndrome”) OR (TI “Post-Acute COVID 19 Neurological Syndrome” OR AB “Post-Acute COVID 19 Neurological Syndrome”) OR (TI “Post Acute COVID19 Neurological Syndrome” OR AB “Post Acute COVID19 Neurological Syndrome”) OR (TI “Post-Acute COVID19 Neurological Syndrome” OR AB “Post-Acute COVID19 Neurological Syndrome”) OR (TI “Long COVID” OR AB “Long COVID”) OR (TI “Long COVID-19” OR AB “Long COVID-19”) OR (TI “Long COVID 19” OR AB “Long COVID 19”) OR (TI “Long COVID19” OR AB “Long COVID19”) OR (TI “Long Haul COVID” OR AB “Long Haul COVID”) OR (TI “Long-Haul COVID” OR AB “Long-Haul COVID”) OR (TI “Long Haul COVID-19” OR AB “Long Haul COVID-19”) OR (TI “Long-Haul COVID-19” OR AB “Long-Haul COVID-19”) OR (TI “Long Haul COVID 19” OR AB “Long Haul COVID 19”) OR (TI “Long-Haul COVID 19” OR AB “Long-Haul COVID 19”) OR (TI “Long Haul COVID19” OR AB “Long Haul COVID19”) OR (TI “Long-Haul COVID19” OR AB “Long-Haul COVID19”) OR (TI “COVID, Long Haul” OR AB “COVID, Long Haul”) OR (TI “COVID, Long-Haul” OR AB “COVID, Long-Haul”) OR (TI “COVID-19, Long Haul” OR AB “COVID-19, Long Haul”) OR (TI “COVID-19, Long-Haul” OR AB “COVID-19, Long-Haul”) OR (TI “COVID 19, Long Haul” OR AB “COVID 19, Long Haul”) OR (TI “COVID 19, Long-Haul” OR AB “COVID 19, Long-Haul”) OR (TI “COVID19, Long Haul” OR AB “COVID19, Long Haul”) OR (TI “COVID19, Long-Haul” OR AB “COVID19, Long-Haul”) OR (TI “Long Hauler COVID” OR AB “Long Hauler COVID”) OR (TI “Long-Hauler COVID” OR AB “Long-Hauler COVID”) OR (TI “Long Hauler COVID-19” OR AB “Long Hauler COVID-19”) OR (TI “Long-Hauler COVID-19” OR AB “Long-Hauler COVID-19”) OR (TI “Long Hauler COVID 19” OR AB “Long Hauler COVID 19”) OR (TI “Long-Hauler COVID 19” OR AB “Long-Hauler COVID 19”) OR (TI “Long Hauler COVID19” OR AB “Long Hauler COVID19”) OR (TI “Long-Hauler COVID19” OR AB “Long-Hauler COVID19”) OR (TI “COVID Long Hauler” OR AB “COVID Long Hauler”) OR (TI “COVID Long-Hauler” OR AB “COVID Long-Hauler”) OR (TI “COVID-19 Long Hauler” OR AB “COVID-19 Long Hauler”) OR (TI “COVID-19 Long-Hauler” OR AB “COVID-19 Long-Hauler”) OR (TI “COVID 19 Long Hauler” OR AB “COVID 19 Long Hauler”) OR (TI “COVID 19 Long-Hauler” OR AB “COVID 19 Long-Hauler”) OR (TI “COVID19 Long Hauler” OR AB “COVID19 Long |

(continued)

**S2 Table. Search strategy employed in the systematic searching of each database (continued).**

| **Database** | **Keywords** | **Search #** | **Search query** |
| --- | --- | --- | --- |
|  |  |  | Hauler”) OR (TI “COVID19 Long-Hauler” OR AB “COVID19 Long-Hauler”) OR (TI “Chronic COVID” OR AB “Chronic COVID”) OR (TI “Chronic COVID-19” OR AB “Chronic COVID-19”) OR (TI “Chronic COVID 19” OR AB “Chronic COVID 19”) OR (TI “Chronic COVID19” OR AB “Chronic COVID19”) OR (TI “Chronic COVID Syndrome” OR AB “Chronic COVID Syndrome”) OR (TI “Chronic COVID-19 Syndrome” OR AB “Chronic COVID-19 Syndrome”) OR (TI “Chronic COVID 19 Syndrome” OR AB “Chronic COVID 19 Syndrome”) OR (TI “Chronic COVID19 Syndrome” OR AB “Chronic COVID19 Syndrome”)) |
|  |  | 6 | #4 OR #5 |
|  | **HRQoL** | 7 | Limited to ‘Humans’: (MH “Quality of Life”) |
|  |  | 8 | Limited to ‘Humans’: ((TI “Quality of Life” OR AB “Quality of Life”) OR (TI “Life Quality” OR AB “Life Quality”) OR (TI “Health Related Quality of Life” OR AB “Health Related Quality of Life”) OR (TI “Health-Related Quality of Life” OR AB “Health-Related Quality of Life”) OR (TI “HRQL” OR AB “HRQL”) OR (TI “HRQoL” OR AB “HRQoL”) OR (TI “Functional Status” OR AB “Functional Status”) OR (TI “Functional Capacity” OR AB “Functional Capacity”) OR (TI “Wellbeing” OR AB “Wellbeing”) OR (TI “Well Being” OR AB “Well Being”) OR (TI “Well-Being” OR AB “Well-Being”)) |
|  |  | 9 | #7 OR #8 |
|  | **Combined** | 10 | Limited to publication dates between 1^st^ January 2003 and 23^rd^ July 2024: (#3 OR #6) AND #9 |
| **Embase** | **ME/CFS** | 1 | Limited to ‘Humans’: “Chronic Fatigue Syndrome”/exp |
|  |  | 2 | Limited to ‘Humans’: (“Chronic Fatigue Syndrome”:ab,ti OR “Chronic Fatigue Syndromes”:ab,ti OR “Fatigue Syndrome, Chronic”:ab,ti OR “Fatigue Syndromes, Chronic”:ab,ti OR “Syndrome, Chronic Fatigue”:ab,ti OR “Syndrome of Chronic Fatigue”:ab,ti OR “Chronic Fatigue Disorder”:ab,ti OR “Chronic Fatigue Disorders”:ab,ti OR “Fatigue Disorder, Chronic”:ab,ti OR “Fatigue Disorders, Chronic”:ab,ti OR “Disorders, Chronic Fatigue”:ab,ti OR “Chronic Fatigue and Immune Dysfunction Syndrome”:ab,ti OR “Chronic Fatigue Fibromyalgia Syndrome”:ab,ti OR “Chronic Fatigue-Fibromyalgia Syndrome”:ab,ti OR “Chronic Fatigue Fibromyalgia Syndromes”:ab,ti OR “Chronic Fatigue-Fibromyalgia Syndromes”:ab,ti OR “Fatigue Fibromyalgia Syndrome, Chronic”:ab,ti OR “Fatigue-Fibromyalgia Syndrome, Chronic”:ab,ti OR “Fatigue Fibromyalgia Syndromes, Chronic”:ab,ti OR “Fatigue-Fibromyalgia Syndromes, Chronic”:ab,ti OR “Syndrome, Chronic Fatigue Fibromyalgia”:ab,ti OR “Syndrome, Chronic Fatigue-Fibromyalgia”:ab,ti OR “Syndromes, Chronic Fatigue Fibromyalgia”:ab,ti OR “Syndromes, Chronic Fatigue-Fibromyalgia”:ab,ti OR “Myalgic Encephalomyelitis”:ab,ti OR “Encephalomyelitis, Myalgic”:ab,ti OR “Systemic Exertion Intolerance Disease”:ab,ti OR “Postviral Fatigue Syndrome”:ab,ti OR “Postviral Fatigue Syndromes”:ab,ti OR “Post Viral Fatigue Syndrome”:ab,ti OR “Post Viral Fatigue Syndromes”:ab,ti OR “Post-Viral Fatigue Syndrome”:ab,ti OR “Post-Viral Fatigue Syndromes”:ab,ti OR “Fatigue Syndrome, Postviral”:ab,ti OR “Fatigue Syndromes, Postviral”:ab,ti OR “Fatigue Syndrome, Post Viral”:ab,ti OR “Fatigue Syndromes, Post Viral”:ab,ti OR “Fatigue Syndrome, Post-Viral”:ab,ti OR “Fatigue Syndromes, Post-Viral”:ab,ti OR “Syndrome, Postviral Fatigue”:ab,ti OR “Syndromes, Postviral Fatigue”:ab,ti OR “Syndrome, Post Viral Fatigue”:ab,ti OR “Syndromes, Post Viral Fatigue”:ab,ti OR “Syndrome, Post-Viral Fatigue”:ab,ti OR “Syndromes, Post-Viral Fatigue”:ab,ti OR “Infectious Mononucleosis Like Syndrome, Chronic”:ab,ti OR |

(continued)

**S2 Table. Search strategy employed in the systematic searching of each database (continued).**

| **Database** | **Keywords** | **Search #** | **Search query** |
| --- | --- | --- | --- |
|  |  |  | “Infectious Mononucleosis-Like Syndrome, Chronic”:ab,ti OR “Royal Free Disease”:ab,ti OR “Akureyri Disease”:ab,ti OR “Epidemic Neuromyasthenia”:ab,ti OR “Iceland Disease”:ab,ti) |
|  |  | 3 | #1 OR #2 |
|  | **PCC** | 4 | Limited to ‘Humans’: “Long COVID”/exp |
|  |  | 5 | Limited to ‘Humans’: (“Post COVID Condition”:ab,ti OR “Post COVID Conditions”:ab,ti OR “Post-COVID Condition”:ab,ti OR “Post-COVID Conditions”:ab,ti OR “Post COVID-19 Condition”:ab,ti OR “Post COVID-19 Conditions”:ab,ti OR “Post-COVID-19 Condition”:ab,ti OR “Post-COVID-19 Conditions”:ab,ti OR “Post COVID 19 Condition”:ab,ti OR “Post COVID 19 Conditions”:ab,ti OR “Post-COVID 19 Condition”:ab,ti OR “Post-COVID 19 Conditions”:ab,ti OR “Post COVID19 Condition”:ab,ti OR “Post COVID19 Conditions”:ab,ti OR “Post-COVID19 Condition”:ab,ti OR “Post-COVID19 Conditions”:ab,ti OR “Post COVID Syndrome”:ab,ti OR “Post-COVID Syndrome”:ab,ti OR “Post COVID-19 Syndrome”:ab,ti OR “Post-COVID-19 Syndrome”:ab,ti OR “Post COVID 19 Syndrome”:ab,ti OR “Post-COVID 19 Syndrome”:ab,ti OR “Post COVID19 Syndrome”:ab,ti OR “Post-COVID19 Syndrome”:ab,ti OR “Post COVID Fatigue”:ab,ti OR “Post-COVID Fatigue”:ab,ti OR “Post COVID-19 Fatigue”:ab,ti OR “Post-COVID-19 Fatigue”:ab,ti OR “Post COVID 19 Fatigue”:ab,ti OR “Post-COVID 19 Fatigue”:ab,ti OR “Post COVID19 Fatigue”:ab,ti OR “Post-COVID19 Fatigue”:ab,ti OR “Post COVID Neurological Syndrome”:ab,ti OR “Post-COVID Neurological Syndrome”:ab,ti OR “Post COVID-19 Neurological Syndrome”:ab,ti OR “Post-COVID-19 Neurological Syndrome”:ab,ti OR “Post COVID 19 Neurological Syndrome”:ab,ti OR “Post-COVID 19 Neurological Syndrome”:ab,ti OR “Post COVID19 Neurological Syndrome”:ab,ti OR “Post-COVID19 Neurological Syndrome”:ab,ti OR “Post COVID Impairment”:ab,ti OR “Post-COVID Impairment”:ab,ti OR “Post COVID-19 Impairment”:ab,ti OR “Post-COVID-19 Impairment”:ab,ti OR “Post COVID 19 Impairment”:ab,ti OR “Post-COVID 19 Impairment”:ab,ti OR “Post COVID19 Impairment”:ab,ti OR “Post-COVID19 Impairment”:ab,ti OR “PASC”:ab,ti OR “Post Acute Sequelae of COVID”:ab,ti OR “Post-Acute Sequelae of COVID”:ab,ti OR “Post Acute Sequelae of COVID-19”:ab,ti OR “Post-Acute Sequelae of COVID-19”:ab,ti OR “Post Acute Sequelae of COVID 19”:ab,ti OR “Post-Acute Sequelae of COVID 19”:ab,ti OR “Post Acute Sequelae of COVID19”:ab,ti OR “Post-Acute Sequelae of COVID19”:ab,ti OR “COVID Post Acute Sequelae”:ab,ti OR “COVID Post-Acute Sequelae”:ab,ti OR “COVID-19 Post Acute Sequelae”:ab,ti OR “COVID-19 Post-Acute Sequelae”:ab,ti OR “COVID 19 Post Acute Sequelae”:ab,ti OR “COVID 19 Post-Acute Sequelae”:ab,ti OR “COVID19 Post Acute Sequelae”:ab,ti OR “COVID19 Post-Acute Sequelae”:ab,ti OR “Post Acute Sequelae of SARS-CoV-2”:ab,ti OR “Post-Acute Sequelae of SARS-CoV-2”:ab,ti OR “Post Acute Sequelae of SARS CoV 2”:ab,ti OR “Post-Acute Sequelae of SARS CoV 2”:ab,ti OR “Post Acute COVID Syndrome”:ab,ti OR “Post Acute COVID Syndromes”:ab,ti OR “Post-Acute COVID Syndrome”:ab,ti OR “Post-Acute COVID Syndromes”:ab,ti OR “Post Acute COVID-19 Syndrome”:ab,ti OR “Post Acute COVID-19 Syndromes”:ab,ti OR “Post-Acute COVID-19 Syndrome”:ab,ti OR “Post-Acute COVID-19 Syndromes”:ab,ti OR “Post Acute COVID 19 Syndrome”:ab,ti OR “Post Acute COVID 19 Syndromes”:ab,ti OR “Post-Acute COVID 19 Syndrome”:ab,ti OR “Post-Acute COVID 19 Syndromes”:ab,ti OR “Post Acute COVID19 Syndrome”:ab,ti OR “Post Acute COVID19 Syndromes”:ab,ti OR “Post-Acute COVID19 Syndrome”:ab,ti OR “Post-Acute COVID19 Syndromes”:ab,ti OR |

(continued)

**S2 Table. Search strategy employed in the systematic searching of each database (continued).**

| **Database** | **Keywords** | **Search #** | **Search query** |
| --- | --- | --- | --- |
|  |  |  | “COVID Syndrome, Post Acute”:ab,ti OR “COVID Syndrome, Post-Acute”:ab,ti OR “COVID-19 Syndrome, Post Acute”:ab,ti OR “COVID-19 Syndrome, Post-Acute”:ab,ti OR “COVID 19 Syndrome, Post Acute”:ab,ti OR “COVID 19 Syndrome, Post-Acute”:ab,ti OR “COVID19 Syndrome, Post Acute”:ab,ti OR “COVID19 Syndrome, Post-Acute”:ab,ti OR “Post Acute COVID Fatigue”:ab,ti OR “Post-Acute COVID Fatigue”:ab,ti OR “Post Acute COVID-19 Fatigue”:ab,ti OR “Post-Acute COVID-19 Fatigue”:ab,ti OR “Post Acute COVID 19 Fatigue”:ab,ti OR “Post-Acute COVID 19 Fatigue”:ab,ti OR “Post Acute COVID19 Fatigue”:ab,ti OR “Post-Acute COVID19 Fatigue”:ab,ti OR “Post Acute COVID Neurological Syndrome”:ab,ti OR “Post-Acute COVID Neurological Syndrome”:ab,ti OR “Post Acute COVID-19 Neurological Syndrome”:ab,ti OR “Post-Acute COVID-19 Neurological Syndrome”:ab,ti OR “Post Acute COVID 19 Neurological Syndrome”:ab,ti OR “Post-Acute COVID 19 Neurological Syndrome”:ab,ti OR “Post Acute COVID19 Neurological Syndrome”:ab,ti OR “Post-Acute COVID19 Neurological Syndrome”:ab,ti OR “Long COVID”:ab,ti OR “Long COVID-19”:ab,ti OR “Long COVID 19”:ab,ti OR “Long COVID19”:ab,ti OR “Long Haul COVID”:ab,ti OR “Long-Haul COVID”:ab,ti OR “Long Haul COVID-19”:ab,ti OR “Long-Haul COVID-19”:ab,ti OR “Long Haul COVID 19”:ab,ti OR “Long-Haul COVID 19”:ab,ti OR “Long Haul COVID19”:ab,ti OR “Long-Haul COVID19”:ab,ti OR “COVID, Long Haul”:ab,ti OR “COVID, Long-Haul”:ab,ti OR “COVID-19, Long Haul”:ab,ti OR “COVID-19, Long-Haul”:ab,ti OR “COVID 19, Long Haul”:ab,ti OR “COVID 19, Long-Haul”:ab,ti OR “COVID19, Long Haul”:ab,ti OR “COVID19, Long-Haul”:ab,ti OR “Long Hauler COVID”:ab,ti OR “Long-Hauler COVID”:ab,ti OR “Long Hauler COVID-19”:ab,ti OR “Long-Hauler COVID-19”:ab,ti OR “Long Hauler COVID 19”:ab,ti OR “Long-Hauler COVID 19”:ab,ti OR “Long Hauler COVID19”:ab,ti OR “Long-Hauler COVID19”:ab,ti OR “COVID Long Hauler”:ab,ti OR “COVID Long-Hauler”:ab,ti OR “COVID-19 Long Hauler”:ab,ti OR “COVID-19 Long-Hauler”:ab,ti OR “COVID 19 Long Hauler”:ab,ti OR “COVID 19 Long-Hauler”:ab,ti OR “COVID19 Long Hauler”:ab,ti OR “COVID19 Long-Hauler”:ab,ti OR “Chronic COVID”:ab,ti OR “Chronic COVID-19”:ab,ti OR “Chronic COVID 19”:ab,ti OR “Chronic COVID19”:ab,ti OR “Chronic COVID Syndrome”:ab,ti OR “Chronic COVID-19 Syndrome”:ab,ti OR “Chronic COVID 19 Syndrome”:ab,ti OR “Chronic COVID19 Syndrome”:ab,ti) |
|  |  | 6 | #4 OR #5 |
|  | **HRQoL** | 7 | Limited to ‘Humans’: “Quality of Life”/exp |
|  |  | 8 | Limited to ‘Humans’: (“Quality of Life”:ab,ti OR “Life Quality”:ab,ti OR “Health Related Quality of Life”:ab,ti OR “Health-Related Quality of Life”:ab,ti OR “HRQL”:ab,ti OR “HRQoL”:ab,ti OR “Functional Status”:ab,ti OR “Functional Capacity”:ab,ti OR “Wellbeing”:ab,ti OR “Well-Being”:ab,ti OR “Well Being”:ab,ti) |
|  |  | 9 | #7 OR #8 |
|  |  | 10 | Limited to publication dates between 1^st^ January 2003 and 23^rd^ July 2024: (#3 OR #6) AND #9 |
| **MEDLINE** | **ME/CFS** | 1 | Limited to ‘Humans’: (MH “Fatigue Syndrome, Chronic”) |
|  |  | 2 | Limited to ‘Humans’: ((TI “Chronic Fatigue Syndrome” OR AB “Chronic Fatigue Syndrome”) OR (TI “Chronic Fatigue Syndromes” OR AB “Chronic Fatigue Syndromes”) OR (TI “Fatigue Syndrome, Chronic” OR AB “Fatigue Syndrome, Chronic”) OR (TI “Fatigue Syndromes, Chronic” OR AB “Fatigue Syndromes, Chronic”) OR (TI “Syndrome, Chronic Fatigue” |

(continued)

**S2 Table. Search strategy employed in the systematic searching of each database (continued).**

| **Database** | **Keywords** | **Search #** | **Search query** |
| --- | --- | --- | --- |
|  |  |  | OR AB “Syndrome, Chronic Fatigue”) OR (TI “Syndrome of Chronic Fatigue” OR AB “Syndrome of Chronic Fatigue”) OR (TI “Chronic Fatigue Disorder” OR AB “Chronic Fatigue Disorder”) OR (TI “Chronic Fatigue Disorders” OR AB “Chronic Fatigue Disorders”) OR (TI “Fatigue Disorder, Chronic” OR AB “Fatigue Disorder, Chronic”) OR (TI “Fatigue Disorders, Chronic” OR AB “Fatigue Disorders, Chronic”) OR (TI “Disorders, Chronic Fatigue” OR AB “Disorders, Chronic Fatigue”) OR (TI “Chronic Fatigue and Immune Dysfunction Syndrome” OR AB “Chronic Fatigue and Immune Dysfunction Syndrome”) OR (TI “Chronic Fatigue Fibromyalgia Syndrome” OR AB “Chronic Fatigue Fibromyalgia Syndrome”) OR (TI “Chronic Fatigue-Fibromyalgia Syndrome” OR AB “Chronic Fatigue-Fibromyalgia Syndrome”) OR (TI “Chronic Fatigue Fibromyalgia Syndromes” OR AB “Chronic Fatigue Fibromyalgia Syndromes”) OR (TI “Chronic Fatigue-Fibromyalgia Syndromes” OR AB “Chronic Fatigue-Fibromyalgia Syndromes”) OR (TI “Fatigue Fibromyalgia Syndrome, Chronic” OR AB “Fatigue Fibromyalgia Syndrome, Chronic”) OR (TI “Fatigue-Fibromyalgia Syndrome, Chronic” OR AB “Fatigue-Fibromyalgia Syndrome, Chronic”) OR (TI “Fatigue Fibromyalgia Syndromes, Chronic” OR AB “Fatigue Fibromyalgia Syndromes, Chronic”) OR (TI “Fatigue-Fibromyalgia Syndromes, Chronic” OR AB “Fatigue-Fibromyalgia Syndromes, Chronic”) OR (TI “Syndrome, Chronic Fatigue Fibromyalgia” OR AB “Syndrome, Chronic Fatigue Fibromyalgia”) OR (TI “Syndrome, Chronic Fatigue-Fibromyalgia” OR AB “Syndrome, Chronic Fatigue-Fibromyalgia”) OR (TI “Syndromes, Chronic Fatigue Fibromyalgia” OR AB “Syndromes, Chronic Fatigue Fibromyalgia”) OR (TI “Syndromes, Chronic Fatigue-Fibromyalgia” OR AB “Syndromes, Chronic Fatigue-Fibromyalgia”) OR (TI “Myalgic Encephalomyelitis” OR AB “Myalgic Encephalomyelitis”) OR (TI “Encephalomyelitis, Myalgic” OR AB “Encephalomyelitis, Myalgic”) OR (TI “Systemic Exertion Intolerance Disease” OR AB “Systemic Exertion Intolerance Disease”) OR (TI “Postviral Fatigue Syndrome” OR AB “Postviral Fatigue Syndrome”) OR (TI “Postviral Fatigue Syndromes” OR AB “Postviral Fatigue Syndromes”) OR (TI “Post Viral Fatigue Syndrome” OR AB “Post Viral Fatigue Syndrome”) OR (TI “Post Viral Fatigue Syndromes” OR AB “Post Viral Fatigue Syndromes”) OR (TI “Post-Viral Fatigue Syndrome” OR AB “Post-Viral Fatigue Syndrome”) OR (TI “Post-Viral Fatigue Syndromes” OR AB “Post-Viral Fatigue Syndromes”) OR (TI “Fatigue Syndrome, Postviral” OR AB “Fatigue Syndrome, Postviral”) OR (TI “Fatigue Syndromes, Postviral” OR AB “Fatigue Syndromes, Postviral”) OR (TI “Fatigue Syndrome, Post Viral” OR AB “Fatigue Syndrome, Post Viral”) OR (TI “Fatigue Syndromes, Post Viral” OR AB “Fatigue Syndromes, Post Viral”) OR (TI “Fatigue Syndrome, Post-Viral” OR AB “Fatigue Syndrome, Post-Viral”) OR (TI “Fatigue Syndromes, Post-Viral” OR AB “Fatigue Syndromes, Post-Viral”) OR (TI “Syndrome, Postviral Fatigue” OR AB “Syndrome, Postviral Fatigue”) OR (TI “Syndromes, Postviral Fatigue” OR AB “Syndromes, Postviral Fatigue”) OR (TI “Syndrome, Post Viral Fatigue” OR AB “Syndrome, Post Viral Fatigue”) OR (TI “Syndromes, Post Viral Fatigue” OR AB “Syndromes, Post Viral Fatigue”) OR (TI “Syndrome, Post-Viral Fatigue” OR AB “Syndrome, Post-Viral Fatigue”) OR (TI “Syndromes, Post-Viral Fatigue” OR AB “Syndromes, Post-Viral Fatigue”) OR (TI “Infectious Mononucleosis Like Syndrome, Chronic” OR AB “Infectious Mononucleosis Like Syndrome, Chronic”) OR (TI “Infectious Mononucleosis-Like Syndrome, Chronic” OR AB “Infectious Mononucleosis-Like Syndrome, Chronic”) OR (TI “Royal Free Disease” OR AB “Royal Free Disease”) OR (TI “Akureyri Disease” OR AB “Akureyri Disease”) OR (TI “Epidemic Neuromyasthenia” OR AB “Epidemic Neuromyasthenia”) OR (TI “Iceland Disease” OR AB “Iceland Disease”)) |

(continued)

**S2 Table. Search strategy employed in the systematic searching of each database (continued).**

| **Database** | **Keywords** | **Search #** | **Search query** |
| --- | --- | --- | --- |
|  |  | 3 | #1 OR #2 |
|  | **PCC** | 4 | Limited to ‘Humans’: (MH “Post-Acute COVID-19 Syndrome”) |
|  |  | 5 | Limited to ‘Humans’: ((TI “Post COVID Condition” OR AB “Post COVID Condition”) OR (TI “Post COVID Conditions” OR AB “Post COVID Conditions”) OR (TI “Post-COVID Condition” OR AB “Post-COVID Condition”) OR (TI “Post-COVID Conditions” OR AB “Post-COVID Conditions”) OR (TI “Post COVID-19 Condition” OR AB “Post COVID-19 Condition”) OR (TI “Post COVID-19 Conditions” OR AB “Post COVID-19 Conditions”) OR (TI “Post-COVID-19 Condition” OR AB “Post-COVID-19 Condition”) OR (TI “Post-COVID-19 Conditions” OR AB “Post-COVID-19 Conditions”) OR (TI “Post COVID 19 Condition” OR AB “Post COVID 19 Condition”) OR (TI “Post COVID 19 Conditions” OR AB “Post COVID 19 Conditions”) OR (TI “Post-COVID 19 Condition” OR AB “Post-COVID 19 Condition”) OR (TI “Post-COVID 19 Conditions” OR AB “Post-COVID 19 Conditions”) OR (TI “Post COVID19 Condition” OR AB “Post COVID19 Condition”) OR (TI “Post COVID19 Conditions” OR AB “Post COVID19 Conditions”) OR (TI “Post-COVID19 Condition” OR AB “Post-COVID19 Condition”) OR (TI “Post-COVID19 Conditions” OR AB “Post-COVID19 Conditions”) OR (TI “Post COVID Syndrome” OR AB “Post COVID Syndrome”) OR (TI “Post-COVID Syndrome” OR AB “Post-COVID Syndrome”) OR (TI “Post COVID-19 Syndrome” OR AB “Post COVID-19 Syndrome”) OR (TI “Post-COVID-19 Syndrome” OR AB “Post-COVID-19 Syndrome”) OR (TI “Post COVID 19 Syndrome” OR AB “Post COVID 19 Syndrome”) OR (TI “Post-COVID 19 Syndrome” OR AB “Post-COVID 19 Syndrome”) OR (TI “Post COVID19 Syndrome” OR AB “Post COVID19 Syndrome”) OR (TI “Post-COVID19 Syndrome” OR AB “Post-COVID19 Syndrome”) OR (TI “Post COVID Fatigue” OR AB “Post COVID Fatigue”) OR (TI “Post-COVID Fatigue” OR AB “Post-COVID Fatigue”) OR (TI “Post COVID-19 Fatigue” OR AB “Post COVID-19 Fatigue”) OR (TI “Post-COVID-19 Fatigue” OR AB “Post-COVID-19 Fatigue”) OR (TI “Post COVID 19 Fatigue” OR AB “Post COVID 19 Fatigue”) OR (TI “Post-COVID 19 Fatigue” OR AB “Post-COVID 19 Fatigue”) OR (TI “Post COVID19 Fatigue” OR AB “Post COVID19 Fatigue”) OR (TI “Post-COVID19 Fatigue” OR AB “Post-COVID19 Fatigue”) OR (TI “Post COVID Neurological Syndrome” OR AB “Post COVID Neurological Syndrome”) OR (TI “Post-COVID Neurological Syndrome” OR AB “Post-COVID Neurological Syndrome”) OR (TI “Post COVID-19 Neurological Syndrome” OR AB “Post COVID-19 Neurological Syndrome”) OR (TI “Post-COVID-19 Neurological Syndrome” OR AB “Post-COVID-19 Neurological Syndrome”) OR (TI “Post COVID 19 Neurological Syndrome” OR AB “Post COVID 19 Neurological Syndrome”) OR (TI “Post-COVID 19 Neurological Syndrome” OR AB “Post-COVID 19 Neurological Syndrome”) OR (TI “Post COVID19 Neurological Syndrome” OR AB “Post COVID19 Neurological Syndrome”) OR (TI “Post-COVID19 Neurological Syndrome” OR AB “Post-COVID19 Neurological Syndrome”) OR (TI “Post COVID Impairment” OR AB “Post COVID Impairment”) OR (TI “Post-COVID Impairment” OR AB “Post-COVID Impairment”) OR (TI “Post COVID-19 Impairment” OR AB “Post COVID-19 Impairment”) OR (TI “Post-COVID-19 Impairment” OR AB “Post-COVID-19 Impairment”) OR (TI “Post COVID 19 Impairment” OR AB “Post COVID 19 Impairment”) OR (TI “Post-COVID 19 Impairment” OR AB “Post-COVID 19 Impairment”) OR (TI “Post COVID19 Impairment” OR AB “Post COVID19 Impairment”) OR (TI “Post-COVID19 Impairment” OR AB “Post-COVID19 Impairment”) OR (TI “PASC” OR AB “PASC”) OR (TI “Post Acute Sequelae of |

(continued)

**S2 Table. Search strategy employed in the systematic searching of each database (continued).**

| **Database** | **Keywords** | **Search #** | **Search query** |
| --- | --- | --- | --- |
|  |  |  | COVID” OR AB “Post Acute Sequelae of COVID”) OR (TI “Post-Acute Sequelae of COVID” OR AB “Post-Acute Sequelae of COVID”) OR (TI “Post Acute Sequelae of COVID-19” OR AB “Post Acute Sequelae of COVID-19”) OR (TI “Post-Acute Sequelae of COVID-19” OR AB “Post-Acute Sequelae of COVID-19”) OR (TI “Post Acute Sequelae of COVID 19” OR AB “Post Acute Sequelae of COVID 19”) OR (TI “Post-Acute Sequelae of COVID 19” OR AB “Post-Acute Sequelae of COVID 19”) OR (TI “Post Acute Sequelae of COVID19” OR AB “Post Acute Sequelae of COVID19”) OR (TI “Post-Acute Sequelae of COVID19” OR AB “Post-Acute Sequelae of COVID19”) OR (TI “COVID Post Acute Sequelae” OR AB “COVID Post Acute Sequelae”) OR (TI “COVID Post-Acute Sequelae” OR AB “COVID Post-Acute Sequelae”) OR (TI “COVID-19 Post Acute Sequelae” OR AB “COVID-19 Post Acute Sequelae”) OR (TI “COVID-19 Post-Acute Sequelae” OR AB “COVID-19 Post-Acute Sequelae”) OR (TI “COVID 19 Post Acute Sequelae” OR AB “COVID 19 Post Acute Sequelae”) OR (TI “COVID 19 Post-Acute Sequelae” OR AB “COVID 19 Post-Acute Sequelae”) OR (TI “COVID19 Post Acute Sequelae” OR AB “COVID19 Post Acute Sequelae”) OR (TI “COVID19 Post-Acute Sequelae” OR AB “COVID19 Post-Acute Sequelae”) OR (TI “Post Acute Sequelae of SARS-CoV-2” OR AB “Post Acute Sequelae of SARS-CoV-2”) OR (TI “Post-Acute Sequelae of SARS-CoV-2” OR AB “Post-Acute Sequelae of SARS-CoV-2”) OR (TI “Post Acute Sequelae of SARS CoV 2” OR AB “Post Acute Sequelae of SARS CoV 2”) OR (TI “Post-Acute Sequelae of SARS CoV 2” OR AB “Post-Acute Sequelae of SARS CoV 2”) OR (TI “Post Acute COVID Syndrome” OR AB “Post Acute COVID Syndrome”) OR (TI “Post Acute COVID Syndromes” OR AB “Post Acute COVID Syndromes”) OR (TI “Post-Acute COVID Syndrome” OR AB “Post-Acute COVID Syndrome”) OR (TI “Post-Acute COVID Syndromes” OR AB “Post-Acute COVID Syndromes”) OR (TI “Post Acute COVID-19 Syndrome” OR AB “Post Acute COVID-19 Syndrome”) OR (TI “Post Acute COVID-19 Syndromes” OR AB “Post Acute COVID-19 Syndromes”) OR (TI “Post-Acute COVID-19 Syndrome” OR AB “Post-Acute COVID-19 Syndrome”) OR (TI “Post-Acute COVID-19 Syndromes” OR AB “Post-Acute COVID-19 Syndromes”) OR (TI “Post Acute COVID 19 Syndrome” OR AB “Post Acute COVID 19 Syndrome”) OR (TI “Post Acute COVID 19 Syndromes” OR AB “Post Acute COVID 19 Syndromes”) OR (TI “Post-Acute COVID 19 Syndrome” OR AB “Post-Acute COVID 19 Syndrome”) OR (TI “Post-Acute COVID 19 Syndromes” OR AB “Post-Acute COVID 19 Syndromes”) OR (TI “Post Acute COVID19 Syndrome” OR AB “Post Acute COVID19 Syndrome”) OR (TI “Post Acute COVID19 Syndromes” OR AB “Post Acute COVID19 Syndromes”) OR (TI “Post-Acute COVID19 Syndrome” OR AB “Post-Acute COVID19 Syndrome”) OR (TI “Post-Acute COVID19 Syndromes” OR AB “Post-Acute COVID19 Syndromes”) OR (TI “COVID Syndrome, Post Acute” OR AB “COVID Syndrome, Post Acute”) OR (TI “COVID Syndrome, Post-Acute” OR AB “COVID Syndrome, Post-Acute”) OR (TI “COVID-19 Syndrome, Post Acute” OR AB “COVID-19 Syndrome, Post Acute”) OR (TI “COVID-19 Syndrome, Post-Acute” OR AB “COVID-19 Syndrome, Post-Acute”) OR (TI “COVID 19 Syndrome, Post Acute” OR AB “COVID 19 Syndrome, Post Acute”) OR (TI “COVID 19 Syndrome, Post-Acute” OR AB “COVID 19 Syndrome, Post-Acute”) OR (TI “COVID19 Syndrome, Post Acute” OR AB “COVID19 Syndrome, Post Acute”) OR (TI “COVID19 Syndrome, Post-Acute” OR AB “COVID19 Syndrome, Post-Acute”) OR (TI “Post Acute COVID Fatigue” OR AB “Post Acute COVID Fatigue”) OR (TI “Post-Acute COVID Fatigue” OR AB “Post-Acute COVID Fatigue”) OR (TI “Post Acute COVID-19 Fatigue” OR AB “Post Acute COVID-19 Fatigue”) OR (TI |

(continued)

**S2 Table. Search strategy employed in the systematic searching of each database (continued).**

| **Database** | **Keywords** | **Search #** | **Search query** |
| --- | --- | --- | --- |
|  |  |  | “Post-Acute COVID-19 Fatigue” OR AB “Post-Acute COVID-19 Fatigue”) OR (TI “Post Acute COVID 19 Fatigue” OR AB “Post Acute COVID 19 Fatigue”) OR (TI “Post-Acute COVID 19 Fatigue” OR AB “Post-Acute COVID 19 Fatigue”) OR (TI “Post Acute COVID19 Fatigue” OR AB “Post Acute COVID19 Fatigue”) OR (TI “Post-Acute COVID19 Fatigue” OR AB “Post-Acute COVID19 Fatigue”) OR (TI “Post Acute COVID Neurological Syndrome” OR AB “Post Acute COVID Neurological Syndrome”) OR (TI “Post-Acute COVID Neurological Syndrome” OR AB “Post-Acute COVID Neurological Syndrome”) OR (TI “Post Acute COVID-19 Neurological Syndrome” OR AB “Post Acute COVID-19 Neurological Syndrome”) OR (TI “Post-Acute COVID-19 Neurological Syndrome” OR AB “Post-Acute COVID-19 Neurological Syndrome”) OR (TI “Post Acute COVID 19 Neurological Syndrome” OR AB “Post Acute COVID 19 Neurological Syndrome”) OR (TI “Post-Acute COVID 19 Neurological Syndrome” OR AB “Post-Acute COVID 19 Neurological Syndrome”) OR (TI “Post Acute COVID19 Neurological Syndrome” OR AB “Post Acute COVID19 Neurological Syndrome”) OR (TI “Post-Acute COVID19 Neurological Syndrome” OR AB “Post-Acute COVID19 Neurological Syndrome”) OR (TI “Long COVID” OR AB “Long COVID”) OR (TI “Long COVID-19” OR AB “Long COVID-19”) OR (TI “Long COVID 19” OR AB “Long COVID 19”) OR (TI “Long COVID19” OR AB “Long COVID19”) OR (TI “Long Haul COVID” OR AB “Long Haul COVID”) OR (TI “Long-Haul COVID” OR AB “Long-Haul COVID”) OR (TI “Long Haul COVID-19” OR AB “Long Haul COVID-19”) OR (TI “Long-Haul COVID-19” OR AB “Long-Haul COVID-19”) OR (TI “Long Haul COVID 19” OR AB “Long Haul COVID 19”) OR (TI “Long-Haul COVID 19” OR AB “Long-Haul COVID 19”) OR (TI “Long Haul COVID19” OR AB “Long Haul COVID19”) OR (TI “Long-Haul COVID19” OR AB “Long-Haul COVID19”) OR (TI “COVID, Long Haul” OR AB “COVID, Long Haul”) OR (TI “COVID, Long-Haul” OR AB “COVID, Long-Haul”) OR (TI “COVID-19, Long Haul” OR AB “COVID-19, Long Haul”) OR (TI “COVID-19, Long-Haul” OR AB “COVID-19, Long-Haul”) OR (TI “COVID 19, Long Haul” OR AB “COVID 19, Long Haul”) OR (TI “COVID 19, Long-Haul” OR AB “COVID 19, Long-Haul”) OR (TI “COVID19, Long Haul” OR AB “COVID19, Long Haul”) OR (TI “COVID19, Long-Haul” OR AB “COVID19, Long-Haul”) OR (TI “Long Hauler COVID” OR AB “Long Hauler COVID”) OR (TI “Long-Hauler COVID” OR AB “Long-Hauler COVID”) OR (TI “Long Hauler COVID-19” OR AB “Long Hauler COVID-19”) OR (TI “Long-Hauler COVID-19” OR AB “Long-Hauler COVID-19”) OR (TI “Long Hauler COVID 19” OR AB “Long Hauler COVID 19”) OR (TI “Long-Hauler COVID 19” OR AB “Long-Hauler COVID 19”) OR (TI “Long Hauler COVID19” OR AB “Long Hauler COVID19”) OR (TI “Long-Hauler COVID19” OR AB “Long-Hauler COVID19”) OR (TI “COVID Long Hauler” OR AB “COVID Long Hauler”) OR (TI “COVID Long-Hauler” OR AB “COVID Long-Hauler”) OR (TI “COVID-19 Long Hauler” OR AB “COVID-19 Long Hauler”) OR (TI “COVID-19 Long-Hauler” OR AB “COVID-19 Long-Hauler”) OR (TI “COVID 19 Long Hauler” OR AB “COVID 19 Long Hauler”) OR (TI “COVID 19 Long-Hauler” OR AB “COVID 19 Long-Hauler”) OR (TI “COVID19 Long Hauler” OR AB “COVID19 Long Hauler”) OR (TI “COVID19 Long-Hauler” OR AB “COVID19 Long-Hauler”) OR (TI “Chronic COVID” OR AB “Chronic COVID”) OR (TI “Chronic COVID-19” OR AB “Chronic COVID-19”) OR (TI “Chronic COVID 19” OR AB “Chronic COVID 19”) OR (TI “Chronic COVID19” OR AB “Chronic COVID19”) OR (TI “Chronic COVID Syndrome” OR AB “Chronic COVID Syndrome”) OR (TI “Chronic COVID-19 Syndrome” OR AB “Chronic COVID-19 Syndrome”) OR (TI |

(continued)

**S2 Table. Search strategy employed in the systematic searching of each database (continued).**

| **Database** | **Keywords** | | **Search #** | **Search query** |
| --- | --- | --- | --- | --- |
|  |  | |  | “Chronic COVID 19 Syndrome” OR AB “Chronic COVID 19 Syndrome”) OR (TI “Chronic COVID19 Syndrome” OR AB “Chronic COVID19 Syndrome”)) |
|  |  | 6 | | #4 OR #5 |
|  | **HRQoL** | 7 | | Limited to ‘Humans’: (MH “Quality of Life”) |
|  |  | 8 | | Limited to ‘Humans’: ((TI “Quality of Life” OR AB “Quality of Life”) OR (TI “Life Quality” OR AB “Life Quality”) OR (TI “Health Related Quality of Life” OR AB “Health Related Quality of Life”) OR (TI “Health-Related Quality of Life” OR AB “Health-Related Quality of Life”) OR (TI “HRQL” OR AB “HRQL”) OR (TI “HRQoL” OR AB “HRQoL”) OR (TI “Functional Status” OR AB “Functional Status”) OR (TI “Functional Capacity” OR AB “Functional Capacity”) OR (TI “Wellbeing” OR AB “Wellbeing”) OR (TI “Well Being” OR AB “Well Being”) OR (TI “Well-Being” OR AB “Well-Being”)) |
|  |  | 9 | | #7 OR #8 |
|  | **Combined** | 10 | | Limited to publication dates between 1^st^ January 2003 and 23^rd^ July 2024: (#3 OR #6) AND #9 |
| **PubMed** | **ME/CFS** | | 1 | Limited to ‘Humans’: “Fatigue Syndrome, Chronic”[MeSH] |
|  |  | | 2 | Limited to ‘Humans’: Limited to ‘Humans’: (“Chronic Fatigue Syndrome”[Title/Abstract] OR “Chronic Fatigue Syndromes”[Title/Abstract] OR “Fatigue Syndrome, Chronic”[Title/Abstract] OR “Fatigue Syndromes, Chronic”[Title/Abstract] OR “Syndrome, Chronic Fatigue”[Title/Abstract] OR “Syndrome of Chronic Fatigue”[Title/Abstract] OR “Chronic Fatigue Disorder”[Title/Abstract] OR “Chronic Fatigue Disorders”[Title/Abstract] OR “Fatigue Disorder, Chronic”[Title/Abstract] OR “Fatigue Disorders, Chronic”[Title/Abstract] OR “Disorders, Chronic Fatigue”[Title/Abstract] OR “Chronic Fatigue and Immune Dysfunction Syndrome”[Title/Abstract] OR “Chronic Fatigue Fibromyalgia Syndrome”[Title/Abstract] OR “Chronic Fatigue-Fibromyalgia Syndrome”[Title/Abstract] OR “Chronic Fatigue Fibromyalgia Syndromes”[Title/Abstract] OR “Chronic Fatigue-Fibromyalgia Syndromes”[Title/Abstract] OR “Fatigue Fibromyalgia Syndrome, Chronic”[Title/Abstract] OR “Fatigue-Fibromyalgia Syndrome, Chronic”[Title/Abstract] OR “Fatigue Fibromyalgia Syndromes, Chronic”[Title/Abstract] OR “Fatigue-Fibromyalgia Syndromes, Chronic”[Title/Abstract] OR “Syndrome, Chronic Fatigue Fibromyalgia”[Title/Abstract] OR “Syndrome, Chronic Fatigue-Fibromyalgia”[Title/Abstract] OR “Syndromes, Chronic Fatigue Fibromyalgia”[Title/Abstract] OR “Syndromes, Chronic Fatigue-Fibromyalgia”[Title/Abstract] OR “Myalgic Encephalomyelitis”[Title/Abstract] OR “Encephalomyelitis, Myalgic”[Title/Abstract] OR “Systemic Exertion Intolerance Disease”[Title/Abstract] OR “Postviral Fatigue Syndrome”[Title/Abstract] OR “Postviral Fatigue Syndromes”[Title/Abstract] OR “Post Viral Fatigue Syndrome”[Title/Abstract] OR “Post Viral Fatigue Syndromes”[Title/Abstract] OR “Post-Viral Fatigue Syndrome”[Title/Abstract] OR “Post-Viral Fatigue Syndromes”[Title/Abstract] OR “Fatigue Syndrome, Postviral”[Title/Abstract] OR “Fatigue Syndromes, Postviral”[Title/Abstract] OR “Fatigue Syndrome, Post Viral”[Title/Abstract] OR “Fatigue Syndromes, Post Viral”[Title/Abstract] OR “Fatigue Syndrome, Post-Viral”[Title/Abstract] OR “Fatigue Syndromes, Post-Viral”[Title/Abstract] OR “Syndrome, Postviral Fatigue”[Title/Abstract] OR “Syndromes, Postviral Fatigue”[Title/Abstract] OR “Syndrome, Post Viral Fatigue”[Title/Abstract] OR “Syndromes, Post Viral Fatigue”[Title/Abstract] OR “Syndrome, Post-Viral Fatigue”[Title/Abstract] OR “Syndromes, Post-Viral |

(continued)

**S2 Table. Search strategy employed in the systematic searching of each database (continued).**

| **Database** | **Keywords** | **Search #** | **Search query** |
| --- | --- | --- | --- |
|  |  |  | Fatigue”[Title/Abstract] OR “Infectious Mononucleosis Like Syndrome, Chronic”[Title/Abstract] OR “Infectious Mononucleosis-Like Syndrome, Chronic”[Title/Abstract] OR “Royal Free Disease”[Title/Abstract] OR “Akureyri Disease”[Title/Abstract] OR “Epidemic Neuromyasthenia”[Title/Abstract] OR “Iceland Disease”[Title/Abstract]) |
|  |  | 3 | #1 OR #2 |
|  | **PCC** | 4 | Limited to ‘Humans’: “Post-Acute COVID-19 Syndrome”[MeSH] |
|  |  |  | Limited to ‘Humans’: (“Post COVID Condition”[Title/Abstract] OR “Post COVID Conditions”[Title/Abstract] OR “Post-COVID Condition”[Title/Abstract] OR “Post-COVID Conditions”[Title/Abstract] OR “Post COVID-19 |
|  |  | 5 | Condition”[Title/Abstract] OR “Post COVID-19 Conditions”[Title/Abstract] OR “Post-COVID-19 Condition”[Title/Abstract] OR “Post-COVID-19 Conditions”[Title/Abstract] OR “Post COVID 19 Condition”[Title/Abstract] OR “Post COVID 19 Conditions”[Title/Abstract] OR “Post-COVID 19 Condition”[Title/Abstract] OR “Post-COVID 19 Conditions”[Title/Abstract] OR “Post COVID19 Condition”[Title/Abstract] OR “Post COVID19 Conditions”[Title/Abstract] OR “Post-COVID19 Condition”[Title/Abstract] OR “Post-COVID19 Conditions”[Title/Abstract] OR “Post COVID Syndrome”[Title/Abstract] OR “Post-COVID Syndrome”[Title/Abstract] OR “Post COVID-19 Syndrome”[Title/Abstract] OR “Post-COVID-19 Syndrome”[Title/Abstract] OR “Post COVID 19 Syndrome”[Title/Abstract] OR “Post-COVID 19 Syndrome”[Title/Abstract] OR “Post COVID19 Syndrome”[Title/Abstract] OR “Post-COVID19 Syndrome”[Title/Abstract] OR “Post COVID Fatigue”[Title/Abstract] OR “Post-COVID Fatigue”[Title/Abstract] OR “Post COVID-19 Fatigue”[Title/Abstract] OR “Post-COVID-19 Fatigue”[Title/Abstract] OR “Post COVID 19 Fatigue”[Title/Abstract] OR “Post-COVID 19 Fatigue”[Title/Abstract] OR “Post COVID19 Fatigue”[Title/Abstract] OR “Post-COVID19 Fatigue”[Title/Abstract] OR “Post COVID Neurological Syndrome”[Title/Abstract] OR “Post-COVID Neurological Syndrome”[Title/Abstract] OR “Post COVID-19 Neurological Syndrome”[Title/Abstract] OR “Post-COVID-19 Neurological Syndrome”[Title/Abstract] OR “Post COVID 19 Neurological Syndrome”[Title/Abstract] OR “Post-COVID 19 Neurological Syndrome”[Title/Abstract] OR “Post COVID19 Neurological Syndrome”[Title/Abstract] OR “Post-COVID19 Neurological Syndrome”[Title/Abstract] OR “Post COVID Impairment”[Title/Abstract] OR “Post-COVID Impairment”[Title/Abstract] OR “Post COVID-19 Impairment”[Title/Abstract] OR “Post-COVID-19 Impairment”[Title/Abstract] OR “Post COVID 19 Impairment”[Title/Abstract] OR “Post-COVID 19 Impairment”[Title/Abstract] OR “Post COVID19 Impairment”[Title/Abstract] OR “Post-COVID19 Impairment”[Title/Abstract] OR “PASC”[Title/Abstract] OR “Post Acute Sequelae of COVID”[Title/Abstract] OR “Post-Acute Sequelae of COVID”[Title/Abstract] OR “Post Acute Sequelae of COVID-19”[Title/Abstract] OR “Post-Acute Sequelae of COVID-19”[Title/Abstract] OR “Post Acute Sequelae of COVID 19”[Title/Abstract] OR “Post-Acute Sequelae of COVID 19”[Title/Abstract] OR “Post Acute Sequelae of COVID19”[Title/Abstract] OR “Post-Acute Sequelae of COVID19”[Title/Abstract] OR “COVID Post Acute Sequelae”[Title/Abstract] OR “COVID Post-Acute Sequelae”[Title/Abstract] OR “COVID-19 Post Acute Sequelae”[Title/Abstract] OR “COVID-19 Post-Acute Sequelae”[Title/Abstract] OR “COVID 19 Post Acute Sequelae”[Title/Abstract] OR “COVID 19 Post-Acute Sequelae”[Title/Abstract] OR “COVID19 Post Acute Sequelae”[Title/Abstract] OR “COVID19 Post-Acute |

(continued)

**S2 Table. Search strategy employed in the systematic searching of each database (continued).**

| **Database** | **Keywords** | **Search #** | **Search query** |
| --- | --- | --- | --- |
|  |  |  | Sequelae”[Title/Abstract] OR “Post Acute Sequelae of SARS-CoV-2”[Title/Abstract] OR “Post-Acute Sequelae of SARS-CoV-2”[Title/Abstract] OR “Post Acute Sequelae of SARS CoV 2”[Title/Abstract] OR “Post-Acute Sequelae of SARS CoV 2”[Title/Abstract] OR “Post Acute COVID Syndrome”[Title/Abstract] OR “Post Acute COVID Syndromes”[Title/Abstract] OR “Post-Acute COVID Syndrome”[Title/Abstract] OR “Post-Acute COVID Syndromes”[Title/Abstract] OR “Post Acute COVID-19 Syndrome”[Title/Abstract] OR “Post Acute COVID-19 Syndromes”[Title/Abstract] OR “Post-Acute COVID-19 Syndrome”[Title/Abstract] OR “Post-Acute COVID-19 Syndromes”[Title/Abstract] OR “Post Acute COVID 19 Syndrome”[Title/Abstract] OR “Post Acute COVID 19 Syndromes”[Title/Abstract] OR “Post-Acute COVID 19 Syndrome”[Title/Abstract] OR “Post-Acute COVID 19 Syndromes”[Title/Abstract] OR “Post Acute COVID19 Syndrome”[Title/Abstract] OR “Post Acute COVID19 Syndromes”[Title/Abstract] OR “Post-Acute COVID19 Syndrome”[Title/Abstract] OR “Post-Acute COVID19 Syndromes”[Title/Abstract] OR “COVID Syndrome, Post Acute”[Title/Abstract] OR “COVID Syndrome, Post-Acute”[Title/Abstract] OR “COVID-19 Syndrome, Post Acute”[Title/Abstract] OR “COVID-19 Syndrome, Post-Acute”[Title/Abstract] OR “COVID 19 Syndrome, Post Acute”[Title/Abstract] OR “COVID 19 Syndrome, Post-Acute”[Title/Abstract] OR “COVID19 Syndrome, Post Acute”[Title/Abstract] OR “COVID19 Syndrome, Post-Acute”[Title/Abstract] OR “Post Acute COVID Fatigue”[Title/Abstract] OR “Post-Acute COVID Fatigue”[Title/Abstract] OR “Post Acute COVID-19 Fatigue”[Title/Abstract] OR “Post-Acute COVID-19 Fatigue”[Title/Abstract] OR “Post Acute COVID 19 Fatigue”[Title/Abstract] OR “Post-Acute COVID 19 Fatigue”[Title/Abstract] OR “Post Acute COVID19 Fatigue”[Title/Abstract] OR “Post-Acute COVID19 Fatigue”[Title/Abstract] OR “Post Acute COVID Neurological Syndrome”[Title/Abstract] OR “Post-Acute COVID Neurological Syndrome”[Title/Abstract] OR “Post Acute COVID-19 Neurological Syndrome”[Title/Abstract] OR “Post-Acute COVID-19 Neurological Syndrome”[Title/Abstract] OR “Post Acute COVID 19 Neurological Syndrome”[Title/Abstract] OR “Post-Acute COVID 19 Neurological Syndrome”[Title/Abstract] OR “Post Acute COVID19 Neurological Syndrome”[Title/Abstract] OR “Post-Acute COVID19 Neurological Syndrome”[Title/Abstract] OR “Long COVID”[Title/Abstract] OR “Long COVID-19”[Title/Abstract] OR “Long COVID 19”[Title/Abstract] OR “Long COVID19”[Title/Abstract] OR “Long Haul COVID”[Title/Abstract] OR “Long-Haul COVID”[Title/Abstract] OR “Long Haul COVID-19”[Title/Abstract] OR “Long-Haul COVID-19”[Title/Abstract] OR “Long Haul COVID 19”[Title/Abstract] OR “Long-Haul COVID 19”[Title/Abstract] OR “Long Haul COVID19”[Title/Abstract] OR “Long-Haul COVID19”[Title/Abstract] OR “COVID, Long Haul”[Title/Abstract] OR “COVID, Long-Haul”[Title/Abstract] OR “COVID-19, Long Haul”[Title/Abstract] OR “COVID-19, Long-Haul”[Title/Abstract] OR “COVID 19, Long Haul”[Title/Abstract] OR “COVID 19, Long-Haul”[Title/Abstract] OR “COVID19, Long Haul”[Title/Abstract] OR “COVID19, Long-Haul”[Title/Abstract] OR “Long Hauler COVID”[Title/Abstract] OR “Long-Hauler COVID”[Title/Abstract] OR “Long Hauler COVID-19”[Title/Abstract] OR “Long-Hauler COVID-19”[Title/Abstract] OR “Long Hauler COVID 19”[Title/Abstract] OR “Long-Hauler COVID 19”[Title/Abstract] OR “Long Hauler COVID19”[Title/Abstract] OR “Long-Hauler COVID19”[Title/Abstract] OR “COVID Long Hauler”[Title/Abstract] OR “COVID Long-Hauler”[Title/Abstract] OR “COVID-19 Long |

(continued)

**S2 Table. Search strategy employed in the systematic searching of each database (continued).**

| **Database** | **Keywords** | **Search #** | **Search query** |
| --- | --- | --- | --- |
|  |  |  | Hauler”[Title/Abstract] OR “COVID-19 Long-Hauler”[Title/Abstract] OR “COVID 19 Long Hauler”[Title/Abstract] OR “COVID 19 Long-Hauler”[Title/Abstract] OR “COVID19 Long Hauler”[Title/Abstract] OR “COVID19 Long-Hauler”[Title/Abstract] OR “Chronic COVID”[Title/Abstract] OR “Chronic COVID-19”[Title/Abstract] OR “Chronic COVID 19”[Title/Abstract] OR “Chronic COVID19”[Title/Abstract] OR “Chronic COVID Syndrome”[Title/Abstract] OR “Chronic COVID-19 Syndrome”[Title/Abstract] OR “Chronic COVID 19 Syndrome”[Title/Abstract] OR “Chronic COVID19 Syndrome”[Title/Abstract]) |
|  |  | 6 | #4 OR #5 |
|  |  | 7 | Limited to ‘Humans’: “Quality of Life”[MeSH] |
|  |  | 8 | Limited to ‘Humans’: (“Quality of Life”[Title/Abstract] OR “Life Quality”[Title/Abstract] OR “Health Related Quality of Life”[Title/Abstract] OR “Health-Related Quality of Life”[Title/Abstract] OR “HRQL”[Title/Abstract] OR “HRQoL”[Title/Abstract] OR “Functional Status”[Title/Abstract] OR “Functional Capacity”[Title/Abstract] OR “Wellbeing”[Title/Abstract] OR “Well-Being”[Title/Abstract] OR “Well Being”[Title/Abstract]) |
|  |  | 9 | #7 OR #8 |
|  |  | 10 | Limited to publication dates between 1^st^ January 2003 and 23^rd^ July 2024: (#3 OR #6) AND #9 |
| **PsycINFO** | **ME/CFS** | 1 | Limited to ‘Humans’: “Chronic Fatigue Syndrome”/ |
|  |  | 2 | Limited to ‘Humans’: (“Chronic Fatigue Syndrome”.ab,ti OR “Chronic Fatigue Syndromes”.ab,ti OR “Fatigue Syndrome, Chronic”.ab,ti OR “Fatigue Syndromes, Chronic”.ab,ti OR “Syndrome, Chronic Fatigue”.ab,ti OR “Syndrome of Chronic Fatigue”.ab,ti OR “Chronic Fatigue Disorder”.ab,ti OR “Chronic Fatigue Disorders”.ab,ti OR “Fatigue Disorder, Chronic”.ab,ti OR “Fatigue Disorders, Chronic”.ab,ti OR “Disorders, Chronic Fatigue”.ab,ti OR “Chronic Fatigue and Immune Dysfunction Syndrome”.ab,ti OR “Chronic Fatigue Fibromyalgia Syndrome”.ab,ti OR “Chronic Fatigue-Fibromyalgia Syndrome”.ab,ti OR “Chronic Fatigue Fibromyalgia Syndromes”.ab,ti OR “Chronic Fatigue-Fibromyalgia Syndromes”.ab,ti OR “Fatigue Fibromyalgia Syndrome, Chronic”.ab,ti OR “Fatigue-Fibromyalgia Syndrome, Chronic”.ab,ti OR “Fatigue Fibromyalgia Syndromes, Chronic”.ab,ti OR “Fatigue-Fibromyalgia Syndromes, Chronic”.ab,ti OR “Syndrome, Chronic Fatigue Fibromyalgia”.ab,ti OR “Syndrome, Chronic Fatigue-Fibromyalgia”.ab,ti OR “Syndromes, Chronic Fatigue Fibromyalgia”.ab,ti OR “Syndromes, Chronic Fatigue-Fibromyalgia”.ab,ti OR “Myalgic Encephalomyelitis”.ab,ti OR “Encephalomyelitis, Myalgic”.ab,ti OR “Systemic Exertion Intolerance Disease”.ab,ti OR “Postviral Fatigue Syndrome”.ab,ti OR “Postviral Fatigue Syndromes”.ab,ti OR “Post Viral Fatigue Syndrome”.ab,ti OR “Post Viral Fatigue Syndromes”.ab,ti OR “Post-Viral Fatigue Syndrome”.ab,ti OR “Post-Viral Fatigue Syndromes”.ab,ti OR “Fatigue Syndrome, Postviral”.ab,ti OR “Fatigue Syndromes, Postviral”.ab,ti OR “Fatigue Syndrome, Post Viral”.ab,ti OR “Fatigue Syndromes, Post Viral”.ab,ti OR “Fatigue Syndrome, Post-Viral”.ab,ti OR “Fatigue Syndromes, Post-Viral”.ab,ti OR “Syndrome, Postviral Fatigue”.ab,ti OR “Syndromes, Postviral Fatigue”.ab,ti OR “Syndrome, Post Viral Fatigue”.ab,ti OR “Syndromes, Post Viral Fatigue”.ab,ti OR “Syndrome, Post-Viral Fatigue”.ab,ti OR “Syndromes, Post-Viral Fatigue”.ab,ti OR “Infectious Mononucleosis Like Syndrome, Chronic”.ab,ti OR “Infectious Mononucleosis-Like Syndrome, Chronic”.ab,ti OR “Royal Free Disease”.ab,ti OR “Akureyri Disease”.ab,ti OR |

(continued)

**S2 Table. Search strategy employed in the systematic searching of each database (continued).**

| **Database** | **Keywords** | **Search #** | **Search query** |
| --- | --- | --- | --- |
|  |  |  | “Epidemic Neuromyasthenia”.ab,ti OR “Iceland Disease”.ab,ti) |
|  |  | 3 | #1 OR #2 |
|  | **PCC** | 4 | Limited to ‘Humans’: “Post-COVID-19 Conditions”/ |
|  |  | 5 | Limited to ‘Humans’: (“Post COVID Condition”.ab,ti OR “Post COVID Conditions”.ab,ti OR “Post-COVID Condition”.ab,ti OR “Post-COVID Conditions”.ab,ti OR “Post COVID-19 Condition”.ab,ti OR “Post COVID-19 Conditions”.ab,ti OR “Post-COVID-19 Condition”.ab,ti OR “Post-COVID-19 Conditions”.ab,ti OR “Post COVID 19 Condition”.ab,ti OR “Post COVID 19 Conditions”.ab,ti OR “Post-COVID 19 Condition”.ab,ti OR “Post-COVID 19 Conditions”.ab,ti OR “Post COVID19 Condition”.ab,ti OR “Post COVID19 Conditions”.ab,ti OR “Post-COVID19 Condition”.ab,ti OR “Post-COVID19 Conditions”.ab,ti OR “Post COVID Syndrome”.ab,ti OR “Post-COVID Syndrome”.ab,ti OR “Post COVID-19 Syndrome”.ab,ti OR “Post-COVID-19 Syndrome”.ab,ti OR “Post COVID 19 Syndrome”.ab,ti OR “Post-COVID 19 Syndrome”.ab,ti OR “Post COVID19 Syndrome”.ab,ti OR “Post-COVID19 Syndrome”.ab,ti OR “Post COVID Fatigue”.ab,ti OR “Post-COVID Fatigue”.ab,ti OR “Post COVID-19 Fatigue”.ab,ti OR “Post-COVID-19 Fatigue”.ab,ti OR “Post COVID 19 Fatigue”.ab,ti OR “Post-COVID 19 Fatigue”.ab,ti OR “Post COVID19 Fatigue”.ab,ti OR “Post-COVID19 Fatigue”.ab,ti OR “Post COVID Neurological Syndrome”.ab,ti OR “Post-COVID Neurological Syndrome”.ab,ti OR “Post COVID-19 Neurological Syndrome”.ab,ti OR “Post-COVID-19 Neurological Syndrome”.ab,ti OR “Post COVID 19 Neurological Syndrome”.ab,ti OR “Post-COVID 19 Neurological Syndrome”.ab,ti OR “Post COVID19 Neurological Syndrome”.ab,ti OR “Post-COVID19 Neurological Syndrome”.ab,ti OR “Post COVID Impairment”.ab,ti OR “Post-COVID Impairment”.ab,ti OR “Post COVID-19 Impairment”.ab,ti OR “Post-COVID-19 Impairment”.ab,ti OR “Post COVID 19 Impairment”.ab,ti OR “Post-COVID 19 Impairment”.ab,ti OR “Post COVID19 Impairment”.ab,ti OR “Post-COVID19 Impairment”.ab,ti OR “PASC”.ab,ti OR “Post Acute Sequelae of COVID”.ab,ti OR “Post-Acute Sequelae of COVID”.ab,ti OR “Post Acute Sequelae of COVID-19”.ab,ti OR “Post-Acute Sequelae of COVID-19”.ab,ti OR “Post Acute Sequelae of COVID 19”.ab,ti OR “Post-Acute Sequelae of COVID 19”.ab,ti OR “Post Acute Sequelae of COVID19”.ab,ti OR “Post-Acute Sequelae of COVID19”.ab,ti OR “COVID Post Acute Sequelae”.ab,ti OR “COVID Post-Acute Sequelae”.ab,ti OR “COVID-19 Post Acute Sequelae”.ab,ti OR “COVID-19 Post-Acute Sequelae”.ab,ti OR “COVID 19 Post Acute Sequelae”.ab,ti OR “COVID 19 Post-Acute Sequelae”.ab,ti OR “COVID19 Post Acute Sequelae”.ab,ti OR “COVID19 Post-Acute Sequelae”.ab,ti OR “Post Acute Sequelae of SARS-CoV-2”.ab,ti OR “Post-Acute Sequelae of SARS-CoV-2”.ab,ti OR “Post Acute Sequelae of SARS CoV 2”.ab,ti OR “Post-Acute Sequelae of SARS CoV 2”.ab,ti OR “Post Acute COVID Syndrome”.ab,ti OR “Post Acute COVID Syndromes”.ab,ti OR “Post-Acute COVID Syndrome”.ab,ti OR “Post-Acute COVID Syndromes”.ab,ti OR “Post Acute COVID-19 Syndrome”.ab,ti OR “Post Acute COVID-19 Syndromes”.ab,ti OR “Post-Acute COVID-19 Syndrome”.ab,ti OR “Post-Acute COVID-19 Syndromes”.ab,ti OR “Post Acute COVID 19 Syndrome”.ab,ti OR “Post Acute COVID 19 Syndromes”.ab,ti OR “Post-Acute COVID 19 Syndrome”.ab,ti OR “Post-Acute COVID 19 Syndromes”.ab,ti OR “Post Acute COVID19 Syndrome”.ab,ti OR “Post Acute COVID19 Syndromes”.ab,ti OR “Post-Acute COVID19 Syndrome”.ab,ti OR “Post-Acute COVID19 Syndromes”.ab,ti OR “COVID Syndrome, Post Acute”.ab,ti OR “COVID Syndrome, Post-Acute”.ab,ti OR “COVID-19 Syndrome, Post Acute”.ab,ti |

(continued)

**S2 Table. Search strategy employed in the systematic searching of each database (continued).**

| **Database** | **Keywords** | **Search #** | **Search query** |
| --- | --- | --- | --- |
|  |  |  | OR “COVID-19 Syndrome, Post-Acute”.ab,ti OR “COVID 19 Syndrome, Post Acute”.ab,ti OR “COVID 19 Syndrome, Post-Acute”.ab,ti OR “COVID19 Syndrome, Post Acute”.ab,ti OR “COVID19 Syndrome, Post-Acute”.ab,ti OR “Post Acute COVID Fatigue”.ab,ti OR “Post-Acute COVID Fatigue”.ab,ti OR “Post Acute COVID-19 Fatigue”.ab,ti OR “Post-Acute COVID-19 Fatigue”.ab,ti OR “Post Acute COVID 19 Fatigue”.ab,ti OR “Post-Acute COVID 19 Fatigue”.ab,ti OR “Post Acute COVID19 Fatigue”.ab,ti OR “Post-Acute COVID19 Fatigue”.ab,ti OR “Post Acute COVID Neurological Syndrome”.ab,ti OR “Post-Acute COVID Neurological Syndrome”.ab,ti OR “Post Acute COVID-19 Neurological Syndrome”.ab,ti OR “Post-Acute COVID-19 Neurological Syndrome”.ab,ti OR “Post Acute COVID 19 Neurological Syndrome”.ab,ti OR “Post-Acute COVID 19 Neurological Syndrome”.ab,ti OR “Post Acute COVID19 Neurological Syndrome”.ab,ti OR “Post-Acute COVID19 Neurological Syndrome”.ab,ti OR “Long COVID”.ab,ti OR “Long COVID-19”.ab,ti OR “Long COVID 19”.ab,ti OR “Long COVID19”.ab,ti OR “Long Haul COVID”.ab,ti OR “Long-Haul COVID”.ab,ti OR “Long Haul COVID-19”.ab,ti OR “Long-Haul COVID-19”.ab,ti OR “Long Haul COVID 19”.ab,ti OR “Long-Haul COVID 19”.ab,ti OR “Long Haul COVID19”.ab,ti OR “Long-Haul COVID19”.ab,ti OR “COVID, Long Haul”.ab,ti OR “COVID, Long-Haul”.ab,ti OR “COVID-19, Long Haul”.ab,ti OR “COVID-19, Long-Haul”.ab,ti OR “COVID 19, Long Haul”.ab,ti OR “COVID 19, Long-Haul”.ab,ti OR “COVID19, Long Haul”.ab,ti OR “COVID19, Long-Haul”.ab,ti OR “Long Hauler COVID”.ab,ti OR “Long-Hauler COVID”.ab,ti OR “Long Hauler COVID-19”.ab,ti OR “Long-Hauler COVID-19”.ab,ti OR “Long Hauler COVID 19”.ab,ti OR “Long-Hauler COVID 19”.ab,ti OR “Long Hauler COVID19”.ab,ti OR “Long-Hauler COVID19”.ab,ti OR “COVID Long Hauler”.ab,ti OR “COVID Long-Hauler”.ab,ti OR “COVID-19 Long Hauler”.ab,ti OR “COVID-19 Long-Hauler”.ab,ti OR “COVID 19 Long Hauler”.ab,ti OR “COVID 19 Long-Hauler”.ab,ti OR “COVID19 Long Hauler”.ab,ti OR “COVID19 Long-Hauler”.ab,ti OR “Chronic COVID”.ab,ti OR “Chronic COVID-19”.ab,ti OR “Chronic COVID 19”.ab,ti OR “Chronic COVID19”.ab,ti OR “Chronic COVID Syndrome”.ab,ti OR “Chronic COVID-19 Syndrome”.ab,ti OR “Chronic COVID 19 Syndrome”.ab,ti OR “Chronic COVID19 Syndrome”.ab,ti) |
|  |  | 6 | #4 OR #5 |
|  | **HRQoL** | 7 | Limited to ‘Humans’: “Quality of Life”/ |
|  |  | 8 | Limited to ‘Humans’: (“Quality of Life”.ab,ti OR “Life Quality”.ab,ti OR “Health Related Quality of Life”.ab,ti OR “Health-Related Quality of Life”.ab,ti OR “HRQL”.ab,ti OR “HRQoL”.ab,ti OR “Functional Status”.ab,ti OR “Functional Capacity”.ab,ti OR “Wellbeing”.ab,ti OR “Well-Being”.ab,ti OR “Well Being”.ab,ti) |
|  |  | 9 | #7 OR #8 |
|  |  | 10 | Limited to publication dates between 1^st^ January 2003 and 23^rd^ July 2024: (#3 OR #6) AND #9 |
| **Web of Science** | **ME/CFS** | 1 | ((TI=“Chronic Fatigue Syndrome” OR AB=“Chronic Fatigue Syndrome”) OR (TI=“Chronic Fatigue Syndromes” OR AB=“Chronic Fatigue Syndromes”) OR (TI=“Fatigue Syndrome, Chronic” OR AB=“Fatigue Syndrome, Chronic”) OR (TI=“Fatigue Syndromes, Chronic” OR AB=“Fatigue Syndromes, Chronic”) OR (TI=“Syndrome, Chronic Fatigue” OR AB=“Syndrome, Chronic Fatigue”) OR (TI=“Syndrome of Chronic Fatigue” OR AB=“Syndrome of Chronic Fatigue”) OR (TI=“Chronic Fatigue Disorder” OR AB=“Chronic Fatigue Disorder”) OR (TI=“Chronic Fatigue Disorders” OR AB=“Chronic |

(continued)

**S2 Table. Search strategy employed in the systematic searching of each database (continued).**

| **Database** | **Keywords** | **Search #** | **Search query** |
| --- | --- | --- | --- |
|  |  |  | Fatigue Disorders”) OR (TI=“Fatigue Disorder, Chronic” OR AB=“Fatigue Disorder, Chronic”) OR (TI=“Fatigue Disorders, Chronic” OR AB=“Fatigue Disorders, Chronic”) OR (TI=“Disorders, Chronic Fatigue” OR AB=“Disorders, Chronic Fatigue”) OR (TI=“Chronic Fatigue and Immune Dysfunction Syndrome” OR AB=“Chronic Fatigue and Immune Dysfunction Syndrome”) OR (TI=“Chronic Fatigue Fibromyalgia Syndrome” OR AB=“Chronic Fatigue Fibromyalgia Syndrome”) OR (TI=“Chronic Fatigue-Fibromyalgia Syndrome” OR AB=“Chronic Fatigue-Fibromyalgia Syndrome”) OR (TI=“Chronic Fatigue Fibromyalgia Syndromes” OR AB=“Chronic Fatigue Fibromyalgia Syndromes”) OR (TI=“Chronic Fatigue-Fibromyalgia Syndromes” OR AB=“Chronic Fatigue-Fibromyalgia Syndromes”) OR (TI=“Fatigue Fibromyalgia Syndrome, Chronic” OR AB=“Fatigue Fibromyalgia Syndrome, Chronic”) OR (TI=“Fatigue-Fibromyalgia Syndrome, Chronic” OR AB=“Fatigue-Fibromyalgia Syndrome, Chronic”) OR (TI=“Fatigue Fibromyalgia Syndromes, Chronic” OR AB=“Fatigue Fibromyalgia Syndromes, Chronic”) OR (TI=“Fatigue-Fibromyalgia Syndromes, Chronic” OR AB=“Fatigue-Fibromyalgia Syndromes, Chronic”) OR (TI=“Syndrome, Chronic Fatigue Fibromyalgia” OR AB=“Syndrome, Chronic Fatigue Fibromyalgia”) OR (TI=“Syndrome, Chronic Fatigue-Fibromyalgia” OR AB=“Syndrome, Chronic Fatigue-Fibromyalgia”) OR (TI=“Syndromes, Chronic Fatigue Fibromyalgia” OR AB=“Syndromes, Chronic Fatigue Fibromyalgia”) OR (TI=“Syndromes, Chronic Fatigue-Fibromyalgia” OR AB=“Syndromes, Chronic Fatigue-Fibromyalgia”) OR (TI=“Myalgic Encephalomyelitis” OR AB=“Myalgic Encephalomyelitis”) OR (TI=“Encephalomyelitis, Myalgic” OR AB=“Encephalomyelitis, Myalgic”) OR (TI=“Systemic Exertion Intolerance Disease” OR AB=“Systemic Exertion Intolerance Disease”) OR (TI=“Postviral Fatigue Syndrome” OR AB=“Postviral Fatigue Syndrome”) OR (TI=“Postviral Fatigue Syndromes” OR AB=“Postviral Fatigue Syndromes”) OR (TI=“Post Viral Fatigue Syndrome” OR AB=“Post Viral Fatigue Syndrome”) OR (TI=“Post Viral Fatigue Syndromes” OR AB=“Post Viral Fatigue Syndromes”) OR (TI=“Post-Viral Fatigue Syndrome” OR AB=“Post-Viral Fatigue Syndrome”) OR (TI=“Post-Viral Fatigue Syndromes” OR AB=“Post-Viral Fatigue Syndromes”) OR (TI=“Fatigue Syndrome, Postviral” OR AB=“Fatigue Syndrome, Postviral”) OR (TI=“Fatigue Syndromes, Postviral” OR AB=“Fatigue Syndromes, Postviral”) OR (TI=“Fatigue Syndrome, Post Viral” OR AB=“Fatigue Syndrome, Post Viral”) OR (TI=“Fatigue Syndromes, Post Viral” OR AB=“Fatigue Syndromes, Post Viral”) OR (TI=“Fatigue Syndrome, Post-Viral” OR AB=“Fatigue Syndrome, Post-Viral”) OR (TI=“Fatigue Syndromes, Post-Viral” OR AB=“Fatigue Syndromes, Post-Viral”) OR (TI=“Syndrome, Postviral Fatigue” OR AB=“Syndrome, Postviral Fatigue”) OR (TI=“Syndromes, Postviral Fatigue” OR AB=“Syndromes, Postviral Fatigue”) OR (TI=“Syndrome, Post Viral Fatigue” OR AB=“Syndrome, Post Viral Fatigue”) OR (TI=“Syndromes, Post Viral Fatigue” OR AB=“Syndromes, Post Viral Fatigue”) OR (TI=“Syndrome, Post-Viral Fatigue” OR AB=“Syndrome, Post-Viral Fatigue”) OR (TI=“Syndromes, Post-Viral Fatigue” OR AB=“Syndromes, Post-Viral Fatigue”) OR (TI=“Infectious Mononucleosis Like Syndrome, Chronic” OR AB=“Infectious Mononucleosis Like Syndrome, Chronic”) OR (TI=“Infectious Mononucleosis-Like Syndrome, Chronic” OR AB=“Infectious Mononucleosis-Like Syndrome, Chronic”) OR (TI=“Royal Free Disease” OR AB=“Royal Free Disease”) OR (TI=“Akureyri Disease” OR AB=“Akureyri Disease”) OR (TI=“Epidemic Neuromyasthenia” OR AB=“Epidemic Neuromyasthenia”) OR (TI=“Iceland Disease” OR AB=“Iceland Disease”) |
|  | **PCC** | 2 | (TI=“Post COVID Condition” OR AB=“Post COVID Condition”) OR (TI=“Post COVID Conditions” OR AB=“Post COVID |

(continued)

**S2 Table. Search strategy employed in the systematic searching of each database (continued).**

| **Database** | **Keywords** | **Search #** | **Search query** |
| --- | --- | --- | --- |
|  |  |  | Conditions”) OR (TI=“Post-COVID Condition” OR AB=“Post-COVID Condition”) OR (TI=“Post-COVID Conditions” OR AB=“Post-COVID Conditions”) OR (TI=“Post COVID-19 Condition” OR AB=“Post COVID-19 Condition”) OR (TI=“Post COVID-19 Conditions” OR AB=“Post COVID-19 Conditions”) OR (TI=“Post-COVID-19 Condition” OR AB=“Post-COVID-19 Condition”) OR (TI=“Post-COVID-19 Conditions” OR AB=“Post-COVID-19 Conditions”) OR (TI=“Post COVID 19 Condition” OR AB=“Post COVID 19 Condition”) OR (TI=“Post COVID 19 Conditions” OR AB=“Post COVID 19 Conditions”) OR (TI=“Post-COVID 19 Condition” OR AB=“Post-COVID 19 Condition”) OR (TI=“Post-COVID 19 Conditions” OR AB=“Post-COVID 19 Conditions”) OR (TI=“Post COVID19 Condition” OR AB=“Post COVID19 Condition”) OR (TI=“Post COVID19 Conditions” OR AB=“Post COVID19 Conditions”) OR (TI=“Post-COVID19 Condition” OR AB=“Post-COVID19 Condition”) OR (TI=“Post-COVID19 Conditions” OR AB=“Post-COVID19 Conditions”) OR (TI=“Post COVID Syndrome” OR AB=“Post COVID Syndrome”) OR (TI=“Post-COVID Syndrome” OR AB=“Post-COVID Syndrome”) OR (TI=“Post COVID-19 Syndrome” OR AB=“Post COVID-19 Syndrome”) OR (TI=“Post-COVID-19 Syndrome” OR AB=“Post-COVID-19 Syndrome”) OR (TI=“Post COVID 19 Syndrome” OR AB=“Post COVID 19 Syndrome”) OR (TI=“Post-COVID 19 Syndrome” OR AB=“Post-COVID 19 Syndrome”) OR (TI=“Post COVID19 Syndrome” OR AB=“Post COVID19 Syndrome”) OR (TI=“Post-COVID19 Syndrome” OR AB=“Post-COVID19 Syndrome”) OR (TI=“Post COVID Fatigue” OR AB=“Post COVID Fatigue”) OR (TI=“Post-COVID Fatigue” OR AB=“Post-COVID Fatigue”) OR (TI=“Post COVID-19 Fatigue” OR AB=“Post COVID-19 Fatigue”) OR (TI=“Post-COVID-19 Fatigue” OR AB=“Post-COVID-19 Fatigue”) OR (TI=“Post COVID 19 Fatigue” OR AB=“Post COVID 19 Fatigue”) OR (TI=“Post-COVID 19 Fatigue” OR AB=“Post-COVID 19 Fatigue”) OR (TI=“Post COVID19 Fatigue” OR AB=“Post COVID19 Fatigue”) OR (TI=“Post-COVID19 Fatigue” OR AB=“Post-COVID19 Fatigue”) OR (TI=“Post COVID Neurological Syndrome” OR AB=“Post COVID Neurological Syndrome”) OR (TI=“Post-COVID Neurological Syndrome” OR AB=“Post-COVID Neurological Syndrome”) OR (TI=“Post COVID-19 Neurological Syndrome” OR AB=“Post COVID-19 Neurological Syndrome”) OR (TI=“Post-COVID-19 Neurological Syndrome” OR AB=“Post-COVID-19 Neurological Syndrome”) OR (TI=“Post COVID 19 Neurological Syndrome” OR AB=“Post COVID 19 Neurological Syndrome”) OR (TI=“Post-COVID 19 Neurological Syndrome” OR AB=“Post-COVID 19 Neurological Syndrome”) OR (TI=“Post COVID19 Neurological Syndrome” OR AB=“Post COVID19 Neurological Syndrome”) OR (TI=“Post-COVID19 Neurological Syndrome” OR AB=“Post-COVID19 Neurological Syndrome”) OR (TI=“Post COVID Impairment” OR AB=“Post COVID Impairment”) OR (TI=“Post-COVID Impairment” OR AB=“Post-COVID Impairment”) OR (TI=“Post COVID-19 Impairment” OR AB=“Post COVID-19 Impairment”) OR (TI=“Post-COVID-19 Impairment” OR AB=“Post-COVID-19 Impairment”) OR (TI=“Post COVID 19 Impairment” OR AB=“Post COVID 19 Impairment”) OR (TI=“Post-COVID 19 Impairment” OR AB=“Post-COVID 19 Impairment”) OR (TI=“Post COVID19 Impairment” OR AB=“Post COVID19 Impairment”) OR (TI=“Post-COVID19 Impairment” OR AB=“Post-COVID19 Impairment”) OR (TI=“PASC” OR AB=“PASC”) OR (TI=“Post Acute Sequelae of COVID” OR AB=“Post Acute Sequelae of COVID”) OR (TI=“Post-Acute Sequelae of COVID” OR AB=“Post-Acute Sequelae of COVID”) OR (TI=“Post Acute Sequelae of COVID-19” OR AB=“Post Acute Sequelae of COVID-19”) OR (TI=“Post-Acute Sequelae of |

(continued)

**S2 Table. Search strategy employed in the systematic searching of each database (continued).**

| **Database** | **Keywords** | **Search #** | **Search query** |
| --- | --- | --- | --- |
|  |  |  | COVID-19” OR AB=“Post-Acute Sequelae of COVID-19”) OR (TI=“Post Acute Sequelae of COVID 19” OR AB=“Post Acute Sequelae of COVID 19”) OR (TI=“Post-Acute Sequelae of COVID 19” OR AB=“Post-Acute Sequelae of COVID 19”) OR (TI=“Post Acute Sequelae of COVID19” OR AB=“Post Acute Sequelae of COVID19”) OR (TI=“Post-Acute Sequelae of COVID19” OR AB=“Post-Acute Sequelae of COVID19”) OR (TI=“COVID Post Acute Sequelae” OR AB=“COVID Post Acute Sequelae”) OR (TI=“COVID Post-Acute Sequelae” OR AB=“COVID Post-Acute Sequelae”) OR (TI=“COVID-19 Post Acute Sequelae” OR AB=“COVID-19 Post Acute Sequelae”) OR (TI=“COVID-19 Post-Acute Sequelae” OR AB=“COVID-19 Post-Acute Sequelae”) OR (TI=“COVID 19 Post Acute Sequelae” OR AB=“COVID 19 Post Acute Sequelae”) OR (TI=“COVID 19 Post-Acute Sequelae” OR AB=“COVID 19 Post-Acute Sequelae”) OR (TI=“COVID19 Post Acute Sequelae” OR AB=“COVID19 Post Acute Sequelae”) OR (TI=“COVID19 Post-Acute Sequelae” OR AB=“COVID19 Post-Acute Sequelae”) OR (TI=“Post Acute Sequelae of SARS-CoV-2” OR AB=“Post Acute Sequelae of SARS-CoV-2”) OR (TI=“Post-Acute Sequelae of SARS-CoV-2” OR AB=“Post-Acute Sequelae of SARS-CoV-2”) OR (TI=“Post Acute Sequelae of SARS CoV 2” OR AB=“Post Acute Sequelae of SARS CoV 2”) OR (TI=“Post-Acute Sequelae of SARS CoV 2” OR AB=“Post-Acute Sequelae of SARS CoV 2”) OR (TI=“Post Acute COVID Syndrome” OR AB=“Post Acute COVID Syndrome”) OR (TI=“Post Acute COVID Syndromes” OR AB=“Post Acute COVID Syndromes”) OR (TI=“Post-Acute COVID Syndrome” OR AB=“Post-Acute COVID Syndrome”) OR (TI=“Post-Acute COVID Syndromes” OR AB=“Post-Acute COVID Syndromes”) OR (TI=“Post Acute COVID-19 Syndrome” OR AB=“Post Acute COVID-19 Syndrome”) OR (TI=“Post Acute COVID-19 Syndromes” OR AB=“Post Acute COVID-19 Syndromes”) OR (TI=“Post-Acute COVID-19 Syndrome” OR AB=“Post-Acute COVID-19 Syndrome”) OR (TI=“Post-Acute COVID-19 Syndromes” OR AB=“Post-Acute COVID-19 Syndromes”) OR (TI=“Post Acute COVID 19 Syndrome” OR AB=“Post Acute COVID 19 Syndrome”) OR (TI=“Post Acute COVID 19 Syndromes” OR AB=“Post Acute COVID 19 Syndromes”) OR (TI=“Post-Acute COVID 19 Syndrome” OR AB=“Post-Acute COVID 19 Syndrome”) OR (TI=“Post-Acute COVID 19 Syndromes” OR AB=“Post-Acute COVID 19 Syndromes”) OR (TI=“Post Acute COVID19 Syndrome” OR AB=“Post Acute COVID19 Syndrome”) OR (TI=“Post Acute COVID19 Syndromes” OR AB=“Post Acute COVID19 Syndromes”) OR (TI=“Post-Acute COVID19 Syndrome” OR AB=“Post-Acute COVID19 Syndrome”) OR (TI=“Post-Acute COVID19 Syndromes” OR AB=“Post-Acute COVID19 Syndromes”) OR (TI=“COVID Syndrome, Post Acute” OR AB=“COVID Syndrome, Post Acute”) OR (TI=“COVID Syndrome, Post-Acute” OR AB=“COVID Syndrome, Post-Acute”) OR (TI=“COVID-19 Syndrome, Post Acute” OR AB=“COVID-19 Syndrome, Post Acute”) OR (TI=“COVID-19 Syndrome, Post-Acute” OR AB=“COVID-19 Syndrome, Post-Acute”) OR (TI=“COVID 19 Syndrome, Post Acute” OR AB=“COVID 19 Syndrome, Post Acute”) OR (TI=“COVID 19 Syndrome, Post-Acute” OR AB=“COVID 19 Syndrome, Post-Acute”) OR (TI=“COVID19 Syndrome, Post Acute” OR AB=“COVID19 Syndrome, Post Acute”) OR (TI=“COVID19 Syndrome, Post-Acute” OR AB=“COVID19 Syndrome, Post-Acute”) OR (TI=“Post Acute COVID Fatigue” OR AB=“Post Acute COVID Fatigue”) OR (TI=“Post-Acute COVID Fatigue” OR AB=“Post-Acute COVID Fatigue”) OR (TI=“Post Acute COVID-19 Fatigue” OR AB=“Post Acute COVID-19 Fatigue”) OR (TI=“Post-Acute COVID-19 Fatigue” OR AB=“Post-Acute COVID-19 Fatigue”) OR (TI=“Post Acute COVID 19 Fatigue” OR AB=“Post Acute COVID 19 |

(continued)

**S2 Table. Search strategy employed in the systematic searching of each database (continued).**

| **Database** | **Keywords** | **Search #** | **Search query** |
| --- | --- | --- | --- |
|  |  |  | Fatigue”) OR (TI=“Post-Acute COVID 19 Fatigue” OR AB=“Post-Acute COVID 19 Fatigue”) OR (TI=“Post Acute COVID19 Fatigue” OR AB=“Post Acute COVID19 Fatigue”) OR (TI=“Post-Acute COVID19 Fatigue” OR AB=“Post-Acute COVID19 Fatigue”) OR (TI=“Post Acute COVID Neurological Syndrome” OR AB=“Post Acute COVID Neurological Syndrome”) OR (TI=“Post-Acute COVID Neurological Syndrome” OR AB=“Post-Acute COVID Neurological Syndrome”) OR (TI=“Post Acute COVID-19 Neurological Syndrome” OR AB=“Post Acute COVID-19 Neurological Syndrome”) OR (TI=“Post-Acute COVID-19 Neurological Syndrome” OR AB=“Post-Acute COVID-19 Neurological Syndrome”) OR (TI=“Post Acute COVID 19 Neurological Syndrome” OR AB=“Post Acute COVID 19 Neurological Syndrome”) OR (TI=“Post-Acute COVID 19 Neurological Syndrome” OR AB=“Post-Acute COVID 19 Neurological Syndrome”) OR (TI=“Post Acute COVID19 Neurological Syndrome” OR AB=“Post Acute COVID19 Neurological Syndrome”) OR (TI=“Post-Acute COVID19 Neurological Syndrome” OR AB=“Post-Acute COVID19 Neurological Syndrome”) OR (TI=“Long COVID” OR AB=“Long COVID”) OR (TI=“Long COVID-19” OR AB=“Long COVID-19”) OR (TI=“Long COVID 19” OR AB=“Long COVID 19”) OR (TI=“Long COVID19” OR AB=“Long COVID19”) OR (TI=“Long Haul COVID” OR AB=“Long Haul COVID”) OR (TI=“Long-Haul COVID” OR AB=“Long-Haul COVID”) OR (TI=“Long Haul COVID-19” OR AB=“Long Haul COVID-19”) OR (TI=“Long-Haul COVID-19” OR AB=“Long-Haul COVID-19”) OR (TI=“Long Haul COVID 19” OR AB=“Long Haul COVID 19”) OR (TI=“Long-Haul COVID 19” OR AB=“Long-Haul COVID 19”) OR (TI=“Long Haul COVID19” OR AB=“Long Haul COVID19”) OR (TI=“Long-Haul COVID19” OR AB=“Long-Haul COVID19”) OR (TI=“COVID, Long Haul” OR AB=“COVID, Long Haul”) OR (TI=“COVID, Long-Haul” OR AB=“COVID, Long-Haul”) OR (TI=“COVID-19, Long Haul” OR AB=“COVID-19, Long Haul”) OR (TI=“COVID-19, Long-Haul” OR AB=“COVID-19, Long-Haul”) OR (TI=“COVID 19, Long Haul” OR AB=“COVID 19, Long Haul”) OR (TI=“COVID 19, Long-Haul” OR AB=“COVID 19, Long-Haul”) OR (TI=“COVID19, Long Haul” OR AB=“COVID19, Long Haul”) OR (TI=“COVID19, Long-Haul” OR AB=“COVID19, Long-Haul”) OR (TI=“Long Hauler COVID” OR AB=“Long Hauler COVID”) OR (TI=“Long-Hauler COVID” OR AB=“Long-Hauler COVID”) OR (TI=“Long Hauler COVID-19” OR AB=“Long Hauler COVID-19”) OR (TI=“Long-Hauler COVID-19” OR AB=“Long-Hauler COVID-19”) OR (TI=“Long Hauler COVID 19” OR AB=“Long Hauler COVID 19”) OR (TI=“Long-Hauler COVID 19” OR AB=“Long-Hauler COVID 19”) OR (TI=“Long Hauler COVID19” OR AB=“Long Hauler COVID19”) OR (TI=“Long-Hauler COVID19” OR AB=“Long-Hauler COVID19”) OR (TI=“COVID Long Hauler” OR AB=“COVID Long Hauler”) OR (TI=“COVID Long-Hauler” OR AB=“COVID Long-Hauler”) OR (TI=“COVID-19 Long Hauler” OR AB=“COVID-19 Long Hauler”) OR (TI=“COVID-19 Long-Hauler” OR AB=“COVID-19 Long-Hauler”) OR (TI=“COVID 19 Long Hauler” OR AB=“COVID 19 Long Hauler”) OR (TI=“COVID 19 Long-Hauler” OR AB=“COVID 19 Long-Hauler”) OR (TI=“COVID19 Long Hauler” OR AB=“COVID19 Long Hauler”) OR (TI=“COVID19 Long-Hauler” OR AB=“COVID19 Long-Hauler”) OR (TI=“Chronic COVID” OR AB=“Chronic COVID”) OR (TI=“Chronic COVID-19” OR AB=“Chronic COVID-19”) OR (TI=“Chronic COVID 19” OR AB=“Chronic COVID 19”) OR (TI=“Chronic COVID19” OR AB=“Chronic COVID19”) OR (TI=“Chronic COVID Syndrome” OR AB=“Chronic COVID Syndrome”) OR (TI=“Chronic COVID-19 Syndrome” OR AB=“Chronic COVID-19 Syndrome”) OR (TI=“Chronic COVID 19 Syndrome” OR AB=“Chronic |

(continued)

**S2 Table. Search strategy employed in the systematic searching of each database (continued).**

| **Database** | **Keywords** | | **Search #** | **Search query** |
| --- | --- | --- | --- | --- |
|  |  | |  | COVID 19 Syndrome”) OR (TI=“Chronic COVID19 Syndrome” OR AB=“Chronic COVID19 Syndrome”) |
|  | **HRQoL** | 3 | | (TI=“Quality of Life” OR AB=“Quality of Life”) OR (TI=“Life Quality” OR AB=“Life Quality”) OR (TI=“Health Related Quality of Life” OR AB=“Health Related Quality of Life”) OR (TI=“Health-Related Quality of Life” OR AB=“Health-Related Quality of Life”) OR (TI=“HRQL” OR AB=“HRQL”) OR (TI=“HRQoL” OR AB=“HRQoL”) OR (TI=“Functional Status” OR AB=“Functional Status”) OR (TI=“Functional Capacity” OR AB=“Functional Capacity”) OR (TI=“Wellbeing” OR AB=“Wellbeing”) OR (TI=“Well Being” OR AB=“Well Being”) OR (TI=“Well-Being” OR AB=“Well-Being”) |
|  | **Combined** | 4 | | Limited to publication dates between 1^st^ January 2003 and 23^rd^ July 2024: (#1 OR #2) AND #3 |

Abbreviations: *CINAHL* Cumulative Index to Nursing and Allied Health Literature; *HRQL* Health-related quality of life; *HRQoL* Health-related quality of life; *ME/CFS* Myalgic Encephalomyelitis/Chronic Fatigue Syndrome; *PCC* Post COVID-19 Condition; *PASC* Post-Acute Sequelae of COVID(-19).

## S11 Table. Summary of quality assessment results using the JBI quality assessment tools for cross-sectional studies [44].

| **Reference** | **Year** | **Criterion number** | | | | | | | |
| --- | --- | --- | --- | --- | --- | --- | --- | --- | --- |
|  |  | **1** | **2** | **3** | **4** | **5** | **6** | **7** | **8** |
| **Ariza *et al.*** [53] | 2024 | Y | Y | Y | Y | Y | Y | Y | U |
| **Cai *et al.*** [54] | 2023 | Y | U | Y | Y | N | N | Y | Y |
| **Calvache-Mateo *et al.*** [55] | 2023 | Y | Y | Y | Y | Y | Y | Y | Y |
| **Cambras *et al.*** [47] | 2018 | U | Y | Y | Y | Y | Y | Y | U |
| **Chang *et al.*** [48] | 2021 | Y | Y | Y | Y | N | N | Y | U |
| **De Sousa *et al.*** [56] | 2022 | Y | Y | Y | Y | N | N | Y | Y |
| **De Vega *et al.*** [49] | 2017 | U | U | Y | Y | N | N | Y | U |
| **Espinar-Herranz *et al.*** [57] | 2023 | Y | U | Y | Y | N | N | Y | Y |
| **Johnston *et al.*** [12] | 2014 | Y | Y | Y | Y | N | N | Y | U |
| **Maroti *et al.*** [50] | 2018 | U | Y | Y | Y | Y | Y | Y | U |
| **Naviaux *et al*.** [46] | 2016 | U | Y | Y | Y | Y | Y | Y | U |
| **Nehme *et al.*** [58] | 2022 | U | Y | Y | Y | Y | Y | Y | U |
| **Ryabkova *et al.*** [51] | 2023 | U | Y | Y | Y | N | N | Y | U |
| **Seeley *et al.*** [59] | 2023 | Y | Y | Y | Y | N | N | Y | Y |
| **Strand *et al.*** [52] | 2019 | Y | U | Y | Y | Y | N | Y | U |
| **Weigel *et al.*** [13] | 2024 | U | Y | Y | Y | Y | Y | Y | Y |

Abbreviations: *JBI* Joanna Briggs Institute; *N* No; *U* Unclear; *Y* Yes

## S12 Table. Justification for each JBI checklist item for cross-sectional studies [44].

| **Reference** | **Year** | **Criterion number** | **Justification** |
| --- | --- | --- | --- |
| **Ariza *et al.*** [53] | 2024 | 1 | Yes. Study participants included a convenience sample of men and women aged between 18 and 65 years who spoke Spanish. Neurological, psychiatric, neurodevelopmental and systemic comorbidities, as well as impairments in motor or sensory function, were considered exclusionary for both pwPCC and HCs. PwPCC receiving care at hospitals in Spain and Andorra were recruited. HCs were recruited via word of mouth from pwPCC participating in the study. Additional exclusion criteria for HCs included having a known history of symptomatic SARS-CoV-2 infection. The study cohort captured participants who were current smokers and those with comorbid conditions, including heart disease, respiratory disease, hypertension, dyslipidaemia, diabetes mellitus, obesity and chronic liver disease. |
|  |  | 2 | Yes. Participants’ age, sex, illness duration, smoking status, education status, change in employment status and household income were reported. |
|  |  | 3 | Yes. The exposure of interest was measured in a valid and reliable way with the WHO case definition for PCC. |
|  |  | 4 | Yes. The exposure of interest was measured in a valid and reliable way with the WHO case definition for PCC. |
|  |  | 5 | Yes. Age, sex, education status, change in employment status, household income and comorbid conditions (including heart disease, respiratory disease, hypertension, dyslipidaemia, diabetes mellitus, obesity and chronic liver disease) were identified as potential confounders. |
|  |  | 6 | Yes. One-way ANOVA models comparing HRQoL outcomes between the four comparator groups were adjusted for age, sex, education status, change in employment status and household income to mitigate these variables as potential confounders. |
|  |  | 7 | Yes. The Spanish version of the EQ-5D-3L and EQ-VAS, the Spanish version of the WHOQOL-BREF and the WHODAS 2.0, were employed to measure the HRQoL outcomes of interest in a valid and reliable way. |
|  |  | 8 | Unclear. Categorical sociodemographic variables were compared using Chi-square tests. Continuous sociodemographic variables and HRQoL domain scores were compared using one-way ANOVA models. These ANOVA models were adjusted for age, sex, education status, change in employment status and household income. Both *F*-statistics and *p*-values were provided. However, confirmation of the variables’ fulfillment of ANOVA assumptions (including normality and homoscedasticity) were not reported. The α-level was 0.05 and *p*-values were adjusted for multiple comparisons using the Bonferroni correction. |
| **Cai *et al.*** [54] | 2023 | 1 | Yes. Study participants were males and females aged at least 18 years. All cases who had received inpatient care for acute COVID-19 illness at Huashan Hospital in Shanghai, China were recruited with PCC status confirmed at six-months follow-up. Additional exclusion criteria for pwPCC included a history of COVID-19 reinfection during the follow-up period. HCs were defined as those with no known history of symptomatic SARS-CoV-2 infection. The study cohort captured people with comorbidity, including hypertension, diabetes mellitus, chronic pulmonary disease, cardio-cerebral vascular disease, chronic liver disease, chronic kidney disease, chronic neurologic disease, rheumatic disease and cancer. |
|  |  | 2 | Unclear. Participants’ age and sex were reported. Comorbidities were identified among the study cohort; however, their prevalence among pwPCC when compared with HCs was not specified. |
|  |  | 3 | Yes. The exposure of interest was measured in a valid and reliable way with the WHO case definition for PCC. |
|  |  | 4 | Yes. The exposure of interest was measured in a valid and reliable way with the WHO case definition for PCC. |
|  |  | 5 | No. The authors identified comorbidities among the study cohort but did not compare prevalence between pwPCC and HCs. Other potential confounders pertinent to the analyses of interest to the present review were not identified. |
|  |  | 6 | No. Approaches to mitigating potential confounding effects in the analyses pertinent to the present review were not stated. |
|  |  | 7 | Yes. The EQ-5D-5L and EQ-VAS were employed to measure the HRQoL outcomes of interest in a reliable way. |
|  |  | 8 | Yes. The α-level was 0.05 and categorical sociodemographic and HRQoL variables were compared using Chi-square tests. However, the authors did not state whether *p*-values resulting from comparisons of HRQoL outcomes among pwPCC and HCs were adjusted for multiple comparisons. Continuous sociodemographic variables were compared with Mann-Whitney *U* tests; however, the means of confirming the non-parametric nature of the continuous sociodemographic variables were not specified. *P*-values but not test statistics were provided. |

(continued)

**S12 Table. Justification for each JBI checklist item for cross-sectional studies [44] (continued).**

| **Reference** | **Year** | **Criterion number** | **Justification** |
| --- | --- | --- | --- |
| **Calvache-Mateo *et al.*** [55] | 2023 | 1 | Yes. Study participants included a convenience sample of males and females aged at least 18 years. Neurological and orthopaedic comorbidities and cognitive impairments were considered exclusionary for both pwPCC and HCs. Additional exclusion criteria for pwPCC included requiring hospitalisation for acute COVID-19 illness, SARS-CoV-2 reinfection and pre-existing chronic pain. The study cohort captured participants who were current smokers and those with comorbid conditions. |
|  |  | 2 | Yes. Participants’ age, sex, illness duration, BMI and smoking status, as well as the routine pain medications taken by the study participants throughout the study, were reported. The proportion of the comparator groups with comorbidities was reported but specific diagnoses are not provided. |
|  |  | 3 | Yes. The exposure of interest was measured in a valid and reliable way with the WHO case definition for PCC. |
|  |  | 4 | Yes. The exposure of interest was measured in a valid and reliable way with the WHO case definition for PCC. |
|  |  | 5 | Yes. Age and sex were identified as potential confounders. |
|  |  | 6 | Yes. PwPCC and HCs were age- and sex-matched with no significant differences in these characteristics between the cohorts. |
|  |  | 7 | Yes. The EQ-5D-5L and EQ-VAS were employed to measure the HRQoL outcomes of interest in a reliable way. |
|  |  | 8 | Yes. Categorical sociodemographic variables were compared using Chi-square tests. Continuous sociodemographic variables and HRQoL domain scores were assessed for normality using the Kolmogorov-Smirnov test for pwPCC and HCs (both *n* ≥ 50). One-way ANOVA models were generated to compare the normally distributed sociodemographic variables between the three comparator groups. However, confirmation of the variables’ fulfillment of ANOVA assumptions (including homoscedasticity) were not reported. All HRQoL domains were non-normally distributed and were compared among the three comparator groups using Kruskal-Wallis *H* tests. The α-level was 0.05 and both test statistics and *p*-values were provided. However, the authors did not state whether *p*-values resulting from comparisons of HRQoL outcomes among pwPCC and HCs had been adjusted for multiple comparisons. |
| **Cambras et al.** [47] | 2018 | 1 | Unclear. Comorbidity, being pregnant and smoking were considered exclusionary for both pwME/CFS and HCs. Additional exclusion criteria for HCs including taking medication that may cause symptoms of fatigue or autonomic dysfunction. However, eligibility based on age, sex or gender was not provided. |
|  |  | 2 | Yes. Participants’ age, sex, illness duration, BMI, employment status and ethnicity, as well as the routine medications taken by the study participants throughout the study, were reported. |
|  |  | 3 | Yes. The exposure of interest was measured in a valid and reliable way with the CCC case definition for ME/CFS. |
|  |  | 4 | Yes. The exposure of interest was measured in a valid and reliable way with the CCC case definition for ME/CFS. |
|  |  | 5 | Yes. Age, sex and BMI were identified as potential confounders. |
|  |  | 6 | Yes. PwME/CFS and HCs were age-, sex- and BMI-matched, with no significant differences in these characteristics between the cohorts. |
|  |  | 7 | Yes. The Spanish version of the SF-36 was employed to measure the HRQoL outcomes of interest in a reliable way. |
|  |  | 8 | Unclear. The authors state that *t*-tests and Mann-Whitney *U* tests were used for normally and non-normally distributed variables, respectively, and the α-level was 0.05. However, the means of determining normality were not provided. As the SF-36 domain scores were non-normally distributed, Mann-Whitney *U* tests were employed to determine if significant differences existed between pwME/CFS and HCs at the two timepoints, with the *p*-values subsequently adjusted for multiple comparisons using the Bonferroni correction. *P*-values but not *U*-statistics were provided. Longitudinal changes in SF-36 scores between the two cohorts were investigated using the Scheirer-Ray-Hare test. Future studies may verify these findings with paired analyses. |
| **Chang *et al*.** [49] | 2021 | 1 | Yes. Study participants included a convenience sample of males and females aged 18 to 70 years. Pregnancy was considered exclusionary for both pwME/CFS and HCs. Additional exclusion criteria for pwME/CFS included spending less than 14 hours per day being sedentary and in a reclined position and returning a score of 70 or more in the Physical Functioning domain of the SF-36. HCs were required to be sedentary for less than 14 hours per day and return a Physical Functioning score of at least 70. |
|  |  | 2 | Yes. Participants’ age, sex, illness duration and BMI were reported. |
|  |  | 3 | Yes. The exposure of interest was measured in a valid and reliable way with the ICC case definition for ME/CFS. |
|  |  | 4 | Yes. The exposure of interest was measured in a valid and reliable way with the ICC case definition for ME/CFS. |
|  |  | 5 | No. Potential confounders pertinent to the analyses of interest to the present review were identified. |

(continued)

**S12 Table. Justification for each JBI checklist item for cross-sectional studies [44] (continued).**

| **Reference** | **Year** | **Criterion number** | **Justification** |
| --- | --- | --- | --- |
|  |  | 6 | No. As no potential confounders were identified for the analyses pertinent to the present review, approaches to mitigating potential confounding effects were not stated. |
|  |  | 7 | Yes. The SF-36 and Karnofsky Performance Status Index were employed to measure the HRQoL outcomes of interest in a reliable way. |
|  |  | 8 | Unclear. Sociodemographic and HRQoL variables were compared among pwPCC and HCs with Wilcoxon signed-rank tests. Future studies may verify these findings with unpaired analyses. *P*-values but not test statistics were provided. The α-level was not provided and the authors did not state whether *p*-values resulting from comparisons of HRQoL outcomes among pwPCC and HCs had been adjusted for multiple comparisons. |
| **De Sousa *et al.*** [54] | 2022 | 1 | Yes. Study participants included a convenience sample of males and females over the age of 18 years who were based in the state of Paraíba, Brazil and reported an absence of neurological or musculoskeletal comorbidity. All pwPCC were required to have a laboratory-confirmed history of SARS-CoV-2 infection not requiring hospital admission. |
|  |  | 2 | Yes. Participants’ age, sex, illness duration and BMI were reported. |
|  |  | 3 | Yes. The exposure of interest was measured in a valid and reliable way, as the study’s eligibility criteria required participants with PCC to present with persistent COVID-19-related symptoms for at least 12 weeks post-infection, consistent with the WHO case definition. |
|  |  | 4 | Yes. Objective, standard criteria were employed to determine the presence of PCC, as the study’s eligibility criteria required participants with PCC to present with persistent COVID-19-related symptoms for at least 12 weeks post-infection, consistent with the WHO case definition. |
|  |  | 5 | No. The authors identified that all participants were unvaccinated for SARS-CoV-2. Other potential confounders pertinent to the analyses of interest to the present review were not identified. |
|  |  | 6 | No. Approaches to mitigating potential confounding effects in the analyses pertinent to the present review were not stated. |
|  |  | 7 | Yes. The Brazilian Portuguese version of the SF-36 was employed to measure the HRQoL outcomes of interest in a valid and reliable way. |
|  |  | 8 | Yes. Categorical sociodemographic variables were compared using Chi-square tests. Continuous sociodemographic variables and HRQoL domain scores were assessed for normality using the Shapiro-Wilk test for pwPCC and HCs (both *n* < 50). Normally distributed sociodemographic variables were compared with independent *t*-tests; however, the means of determining homoscedasticity were not provided. All HRQoL domains were non-normally distributed and were compared using Mann-Whitney *U* tests. *P*-values but not *U*-statistics were provided. The α-level was 0.05; however, the authors did not state whether *p*-values resulting from comparisons of HRQoL outcomes among pwPCC and HCs had been adjusted for multiple comparisons. |
| **De Vega *et al*.** [50] | 2017 | 1 | Unclear. Study participants included a convenience sample of females with a BMI of less than 30. Active HIV, AIDS or Hepatitis C infection was considered exclusionary for both pwME/CFS and HCs. However, eligibility based on age was not provided. |
|  |  | 2 | Unclear. Participants’ age, sex, BMI and ethnicity were reported. |
|  |  | 3 | Yes. The exposure of interest was measured in a valid and reliable way with the CCC case definition for ME/CFS. |
|  |  | 4 | Yes. The exposure of interest was measured in a valid and reliable way with the CCC case definition for ME/CFS. |
|  |  | 5 | No. The authors identified that all participants were female. Other potential confounders pertinent to the analyses of interest to the present review were not identified. |
|  |  | 6 | No. Approaches to mitigating potential confounding effects in the analyses pertinent to the present review were not stated. |
|  |  | 7 | Yes. The RAND-36 version 1.0 was employed to measure the HRQoL outcomes of interest in a reliable way. |
|  |  | 8 | Unclear. Continuous sociodemographic and HRQoL variables were compared among pwME/CFS and HCs with *t*-tests. However, the authors do not state whether the *t*-tests were independent and the means of confirming the parametric nature of these variables were not specified. Level of significance (if *p* < 0.05) but not *p*-values or *t*-statistics were provided. The α-level was 0.05; however, the authors did not state whether *p*-values resulting from comparisons of HRQoL outcomes among pwPCC and HCs had been adjusted for multiple comparisons. |
| **Espinar-Herranz *et al*.** [58] | 2023 | 1 | Yes. Study participants included a convenience sample of men and women aged 18 to 66 years who reported an absence of autoimmune or psychiatric comorbidity, dementia, other chronic disease or visual, auditory or language impairments. Additional exclusion criteria for HCs included having a known history of symptomatic SARS-CoV-2 infection. |
|  |  | 2 | Unclear. Participants’ age, gender, education status and employment status were reported. |

(continued)

**S12 Table. Justification for each JBI checklist item for cross-sectional studies [44] (continued).**

| **Reference** | **Year** | **Criterion number** | **Justification** |
| --- | --- | --- | --- |
|  |  | 3 | Yes. The exposure of interest was measured in a valid and reliable way with the WHO case definition for PCC. |
|  |  | 4 | Yes. The exposure of interest was measured in a valid and reliable way with the WHO case definition for PCC. |
|  |  | 5 | No. Potential confounders pertinent to the analyses of interest to the present review were not identified. |
|  |  | 6 | No. Approaches to mitigating potential confounding effects in the analyses pertinent to the present review were not stated. |
|  |  | 7 | Yes. The Spanish version of the SF-36 was employed to measure the HRQoL outcomes of interest in a reliable way. |
|  |  | 8 | Yes. Continuous sociodemographic variables and HRQoL domain scores were assessed for normality using the Kolmogorov-Smirnov test for pwPCC (*n* ≥ 50) and the Shapiro-Wilk test for HCs (*n* < 50). All variables were non-normally distributed and were compared using Mann-Whitney *U* tests. Both the *U*-statistics and *p*-values were provided. The α-level was 0.05; however, the authors did not state whether *p*-values resulting from comparisons of HRQoL outcomes among pwPCC and HCs had been adjusted for multiple comparisons. |
| **Johnston *et al*.** [12] | 2014 | 1 | Yes. Study participants included a convenience sample of men and women aged 18 to 64 years who reported an absence of comorbidity, not receiving hormonal therapy and residing in South East Queensland, Australia. |
|  |  | 2 | Yes. Participants’ age, gender and illness duration were reported. |
|  |  | 3 | Yes. The exposure of interest was measured in a valid and reliable way with the ICC case definition for ME/CFS. |
|  |  | 4 | Yes. The exposure of interest was measured in a valid and reliable way with the ICC case definition for ME/CFS. |
|  |  | 5 | No. Potential confounders pertinent to the analyses of interest to the present review were not identified. |
|  |  | 6 | No. Approaches to mitigating potential confounding effects in the analyses pertinent to the present review were not stated. |
|  |  | 7 | Yes. The SF-36 and WHODAS 2.0 were employed to measure the HRQoL outcomes of interest in a reliable way. |
|  |  | 8 | Unclear. Categorical sociodemographic variables were compared using Chi-square tests. Continuous sociodemographic variables and HRQoL domain scores were compared between pwME/CFS and HCs with independent *t*-tests. However, the means of confirming the parametric nature of these variables were not specified. Level of significance (if *p* < 0.05) but not *p*-values or *t*-statistics were provided. The α-level was 0.05; however, the authors did not state whether *p*-values resulting from comparisons of HRQoL outcomes among pwME/CFS and HCs had been adjusted for multiple comparisons. |
| **Maroti *et al*.** [51] | 2018 | 1 | Unclear. Study participants included a convenience sample of males and females who reported an absence of untreated comorbidity that explained their symptoms. PwME/CFS receiving care at the Danderyd University Hospital in Danderyd, Sweden were recruited. HCs were recruited via word of mouth from pwME/CFS participating in the study. The study cohort captured people with medical and psychiatric comorbidities that were currently controlled and, for pwME/CFS, were not responsible for the participant’s symptoms. However, eligibility based on age was not provided. |
|  |  | 2 | Yes. Participants’ age, gender, illness duration, education status and receipt of disability pension were reported. |
|  |  | 3 | Yes. The exposure of interest was measured in a valid and reliable way with the CCC case definition for ME/CFS. |
|  |  | 4 | Yes. The exposure of interest was measured in a valid and reliable way with the CCC case definition for ME/CFS. |
|  |  | 5 | Yes. Illness status, gender and illness duration, as well as anxiety and depression scores determined by the Hospital Anxiety and Depression Scale, were identified as potential confounders. |
|  |  | 6 | Yes. MANCOVA models were generated to determine the effect of the potential confounders. Depression was significantly associated with Physical Functioning, General Health, Vitality, Social Functioning, Role Emotion and Mental Component Summary scores. |
|  |  | 7 | Yes. The Swedish version of the SF-36 was employed to measure the HRQoL outcomes of interest in a reliable way. |
|  |  | 8 | Unclear. Categorical sociodemographic variables were compared using Chi-square tests. Continuous sociodemographic variables were compared between the comparator groups using independent *t*-tests and ANOVA models. Additionally, ANOVA models were generated to compare the HRQoL domain scores among the three comparator groups. However, confirmation of the variables’ fulfillment of the assumptions for independent *t*-tests and ANOVA (including normality and homoscedasticity) were not reported. Level of significance (if *p* < 0.05) but not *p*-values or *t*-statistics were provided. The α-level was 0.05; however, the authors did not state whether *p*-values resulting from comparisons of HRQoL outcomes among pwME/CFS and HCs had been adjusted for multiple comparisons. |
| **Naviaux *et al*.** [47] | 2016 | 1 | Unclear. Study participants included a convenience sample of males and females. However, eligibility based on age was not provided. |

(continued)

**S12 Table. Justification for each JBI checklist item for cross-sectional studies [44] (continued).**

| **Reference** | **Year** | **Criterion number** | **Justification** |
| --- | --- | --- | --- |
|  |  | 2 | Yes. Participants’ age, sex, illness duration, BMI, education, ethnicity and number of routine medications were reported. |
|  |  | 3 | Yes. The exposure of interest was measured in a valid and reliable way with the CCC case definition for ME/CFS. |
|  |  | 4 | Yes. The exposure of interest was measured in a valid and reliable way with the CCC case definition for ME/CFS. |
|  |  | 5 | Yes. Age and sex were identified as potential confounders. |
|  |  | 6 | Yes. PwME/CFS and HCs were age- and sex-matched with no significant differences in these characteristics between the cohorts. |
|  |  | 7 | Yes. The Karnofsky Performance Status Index was employed to measure the HRQoL outcomes of interest in a reliable way. |
|  |  | 8 | Unclear. Neither the statistical methods employed to compare HRQoL outcomes between pwME/CFS and HCs nor the α-level were stated. *P*-values but not test statistics were provided. The authors did not state whether *p*-values resulting from comparisons of HRQoL outcomes among pwME/CFS and HCs had been adjusted for multiple comparisons. |
| **Nehme *et al*.** [59] | 2022 | 1 | Unclear. All cases who were tested for SARS-CoV-2 at the outpatient testing centre at the Geneva University Hospitals in Geneva, Switzerland were recruited with PCC status confirmed at seven- and 15-months follow-up. Additional exclusion criteria for pwPCC included a history of COVID-19 reinfection during the follow-up period. HCs were defined as those with no known history of symptomatic SARS-CoV-2 infection. The study cohort captured participants who were current smokers and those with comorbid conditions, including obesity, hypertension, diabetes, respiratory disease, cardiovascular disease, headache disorders, cognitive disorders, sleep disorders, depression, anxiety, hypothyroidism, rheumatologic disease, anaemia, thromboembolic disease, dysmenorrhea, fibromyalgia or chronic pain, CFS, rheumatologic disorders and irritable bowel syndrome. However, eligibility based on age was not provided. |
|  |  | 2 | Yes. Participants’ age, sex, BMI, education status, employment status, smoking status and comorbidities were provided. The illness duration for pwPCC was either seven or 15 months, depending on the persistence of symptoms at follow-up. |
|  |  | 3 | Yes. The exposure of interest was measured in a valid and reliable way, as the study’s eligibility criteria required participants with PCC to present with persistent COVID-19-related symptoms for seven or 15 months post-infection. Hence, all participants with PCC had an illness presentation consistent with the WHO case definition. |
|  |  | 4 | Yes. Objective, standard criteria were employed to determine the presence of PCC, as the study’s eligibility criteria required participants with PCC to present with persistent COVID-19-related symptoms for seven or 15 months post-infection. Hence, all participants with PCC had an illness presentation consistent with the WHO case definition. |
|  |  | 5 | Yes. Age, sex, physical activity, smoking status, vaccination status, hospitalisation for COVID-19, self-rated health prior to testing, symptoms at testing and comorbidities were identified as potential confounders. |
|  |  | 6 | Yes. Comparisons between the comparator groups were adjusted for age, sex, physical activity, smoking status, vaccination status, hospitalisation for COVID-19, self-rated health prior to testing, symptoms at testing and comorbidities to mitigate these variables as potential confounders. |
|  |  | 7 | Yes. The SF-12 and Sheehan Disability Scale were employed to measure the HRQoL outcomes of interest in a valid and reliable way. |
|  |  | 8 | Unclear. Categorical sociodemographic and HRQoL variables were compared between pwPCC and HCs using Chi-square tests and the α-level was 0.05. The authors state that the estimated proportions of impairment for each HRQoL domain had been adjusted for the potential confounders of interest. However, the methods employed to estimate these proportions are not reported. Omnibus *P*-values but not test statistics were provided. Post-hoc tests were not reported. |
| **Ryabkova *et al*.** [52] | 2023 | 1 | Unclear. Study participants included a convenience sample of males and females who reported an absence of autoimmune disease and acute illness within the three months prior to participation. However, eligibility based on age was not provided. |
|  |  | 2 | Yes. Participants’ age, gender, illness duration and BMI were provided. |
|  |  | 3 | Yes. The exposure of interest was measured in a valid and reliable way with the CCC case definition for ME/CFS. |
|  |  | 4 | Yes. The exposure of interest was measured in a valid and reliable way with the CCC case definition for ME/CFS. |
|  |  | 5 | No. Potential confounders pertinent to the analyses of interest to the present review were not identified. |
|  |  | 6 | No. Approaches to mitigating potential confounding effects in the analyses pertinent to the present review were not stated. |
|  |  | 7 | Yes. The SF-36 was employed to measure the HRQoL outcomes of interest in a reliable way. |

(continued)

**S12 Table. Justification for each JBI checklist item for cross-sectional studies [44] (continued).**

| **Reference** | **Year** | **Criterion number** | **Justification** |
| --- | --- | --- | --- |
|  |  | 8 | Unclear. Categorical sociodemographic variables were compared using Chi-square tests. Continuous sociodemographic variables and HRQoL scores were compared between pwME/CFS and HCs with Mann-Whitney *U*-tests. However, the means of confirming the non-parametric nature of these variables were not specified. *P*-values but not test statistics were provided. The α-level was 0.05; however, the authors did not state whether *p*-values resulting from comparisons of HRQoL outcomes among pwME/CFS and HCs had been adjusted for multiple comparisons. |
| **Seeley *et al*.** [60] | 2023 | 1 | Yes. Study participants included a convenience sample of males and females aged at least 18 years. Additional exclusion criteria for HCs included neurological, cardiac, endocrine or immune comorbidity, alcohol or drug dependence, taking daily medications and a history of SARS-CoV-2 infection within the three months prior to participation. The study cohort captured participants with comorbid conditions, including migraines, allergic rhinitis, endometriosis, asthma and generalised joint hypermobility. |
|  |  | 2 | Yes. Participants’ age, sex, illness duration, BMI, education status and ethnicity were provided. |
|  |  | 3 | Yes. The exposure of interest was measured in a valid and reliable way with the WHO case definition for PCC. |
|  |  | 4 | Yes. The exposure of interest was measured in a valid and reliable way with the WHO case definition for PCC. |
|  |  | 5 | No. The authors identified comorbidities and smoking status among the study cohort. Other potential confounders pertinent to the analyses of interest to the preset review were not identified. |
|  |  | 6 | No. Approaches to mitigating potential confounding effects in the analyses pertinent to the present review were not stated. |
|  |  | 7 | Yes. The EQ-5D-5L and EQ-VAS were employed to measure the HRQoL outcomes of interest in a reliable way. |
|  |  | 8 | Yes. Categorical sociodemographic and HRQoL variables were compared between the three comparator groups with Chi-square tests. Kruskal-Wallis *H*-tests were employed to compare non-parametric continuous sociodemographic variables among the comparator groups. The α-level was 0.05 and post-hoc analyses of Kruskal-Wallis *H*-test results were adjusted for multiple comparisons using the Bonferroni correction. However, the means of confirming the non-parametric nature of these variables were not specified. Additionally, the authors did not state whether *p*-values resulting from comparisons of categorical HRQoL outcomes among pwPCC and HCs had been adjusted for multiple comparisons. *P*-values but not test statistics were provided. |
| **Strand *et al*.** [53] | 2019 | 1 | Yes. Study participants included a convenience sample of males and females aged 18 to 65 years who spoke Norwegian. PwME/CFS receiving care at Oslo University Hospital in Oslo, Norway were recruited. HCs were blood donors at the same hospital. |
|  |  | 2 | Unclear. Participants’ age, gender and education status were provided. |
|  |  | 3 | Yes. The exposure of interest was measured in a valid and reliable way with the CCC case definition for ME/CFS. |
|  |  | 4 | Yes. The exposure of interest was measured in a valid and reliable way with the CCC case definition for ME/CFS. |
|  |  | 5 | Yes. Age was identified as a potential confounder. |
|  |  | 6 | No. Approaches to mitigate potential confounders in the analyses pertinent to the present review were not stated. |
|  |  | 7 | Yes. The SF-36 was employed to measure the HRQoL outcomes of interest in a reliable way. |
|  |  | 8 | Unclear. The statistical methods used to compare categorical sociodemographic variables between pwME/CFS and HCs were not stated. Continuous sociodemographic and HRQoL variables were compared between the two cohorts with independent *t*-tests. However, the means of confirming the parametric nature of these variables were not specified. Level of significance (if *p* < 0.05) but not *p*-values or *t*-statistics were provided. The α-level was 0.05; however, the authors did not state whether *p*-values resulting from comparisons of HRQoL outcomes among pwME/CFS and HCs had been adjusted for multiple comparisons. |
| **Weigel *et al*.** [13] | 2024 | 1 | Unclear. Study participants included males and females who reported an absence of comorbidity. However, eligibility based on age was not provided. |
|  |  | 2 | Yes. Participants’ age, sex, illness duration, BMI, employment status and education status were reported. |
|  |  | 3 | Yes. The exposure of interest was measured in a valid and reliable way with the CCC and ICC case definitions for ME/CFS and the WHO case definition for PCC. |
|  |  | 4 | Yes. The exposure of interest was measured in a valid and reliable way with the CCC and ICC case definitions for ME/CFS and the WHO case definition for PCC. |
|  |  | 5 | Yes. Age, sex and illness duration were identified as potential confounders. |

(continued)

**S12 Table. Justification for each JBI checklist item for cross-sectional studies [44] (continued).**

| **Reference** | **Year** | **Criterion number** | **Justification** |
| --- | --- | --- | --- |
|  |  | 6 | Yes. Partial rank correlations comparing HRQoL outcomes between the three comparator groups were adjusted for age, sex and illness duration to mitigate these variables as potential confounders. |
|  |  | 7 | Yes. The SF-36 version 2, WHODAS 2.0 and Dr Bell’s CFIDS Disability Scale were employed to measure the HRQoL outcomes of interest in a reliable way. |
|  |  | 8 | Yes. Categorical sociodemographic variables were compared using Fisher-Freeman-Halton tests. Continuous sociodemographic variables were assessed for normality using the Kolmogorov-Smirnov test for pwME/CFS and HCs (*n* ≥ 50) and the Shapiro-Wilk test for pwPCC (*n* < 50). All continuous sociodemographic variables were non-normally distributed and were compared with Kruskal-Wallis *H* tests. Both test statistics and *p*-values were provided. Partial rank correlations were generated for each of the HRQoL domains (all of which were non-normally distributed) to control for age, sex and illness duration. The α-level was 0.05 and *p*-values were adjusted for multiple comparisons using the Benjamini-Hochberg correction. |

Abbreviations: *ANOVA* Analysis of variance; *BMI* Body mass index; *CCC* Canadian Consensus Criteria; *CFIDS* Chronic Fatigue and Immune Dysfunction Syndrome; *EQ-5D-3L* EuroQol 5-Dimension 3-Level questionnaire; *EQ-5D-5L* EuroQol 5-Dimension 5-Level questionnaire; *EQ-VAS* EuroQol Visual Analogue Scale; *HC* Healthy control; *HRQoL* Health-related quality of life; *ICC* International Consensus Criteria; *JBI* Joanna Briggs Institute; *MANCOVA* Multivariate analysis of covariance; *ME/CFS* Myalgic Encephalomyelitis/Chronic Fatigue Syndrome; *NA* Not applicable; *PCC* Post COVID-19 Condition; *PROM* Patient-reported outcome measure; *PwME/CFS* People with Myalgic Encephalomyelitis/Chronic Fatigue Syndrome; *PwPCC* People with Post COVID-19 Condition; *SF-36* 36-Item Short-Form Health Survey; *WHO* World Health Organization; *WHODAS 2.0* World Health Organization Disability Assessment Schedule version 2.
